# ASFV pA151R negatively regulates type I IFN production via degrading E3 ligase TRAF6

**DOI:** 10.3389/fimmu.2024.1339510

**Published:** 2024-02-21

**Authors:** You Li, Li Huang, Hui Li, Yingqi Zhu, Zilong Yu, Xiaojie Zheng, Changjiang Weng, Wen-hai Feng

**Affiliations:** ^1^State Key Laboratory of Animal Biotech Breeding, China Agricultural University, Beijing, China; ^2^Frontiers Science Center for Molecular Design Breeding, College of Biological Sciences, China Agricultural University, Beijing, China; ^3^Ministry of Agriculture Key Laboratory of Soil Microbiology, College of Biological Sciences, China Agricultural University, Beijing, China; ^4^Department of Microbiology and Immunology, College of Biological Sciences, China Agricultural University, Beijing, China; ^5^State Key Laboratory of Veterinary Biotechnology, Harbin Veterinary Research Institute of Chinese Academy of Agricultural Sciences, Harbin, China

**Keywords:** ASFV, type I IFN, pA151R, TBK1, TRAF6

## Abstract

African swine fever (ASF) caused by African swine fever virus (ASFV) is a highly mortal and hemorrhagic infectious disease in pigs. Previous studies have indicated that ASFV modulates interferon (IFN) production. In this study, we demonstrated that ASFV pA151R negatively regulated type I IFN production. Ectopic expression of pA151R dramatically inhibited K63-linked polyubiquitination and Ser172 phosphorylation of TANK-binding kinase 1 (TBK1). Mechanically, we demonstrated that E3 ligase TNF receptor–associated factor 6 (TRAF6) participated in the ubiquitination of TBK1 in cGAS-STING signaling pathway. We showed that pA151R interacted with TRAF6 and degraded it through apoptosis pathway, leading to the disruption of TBK1 and TRAF6 interaction. Moreover, we clarified that the amino acids H102, C109, C132, and C135 in pA151R were crucial for pA151R to inhibit type I interferon production. In addition, we verified that overexpression of pA151R facilitated DNA virus Herpes simplex virus 1 (HSV-1) replication by inhibiting IFN-β production. Importantly, knockdown of pA151R inhibited ASFV replication and enhanced IFN-β production in porcine alveolar macrophages (PAMs). Our findings will help understand how ASFV escapes host antiviral immune responses and develop effective ASFV vaccines.

## 1 Introduction

African swine fever (ASF) initially reported in Kenya in 1921 is an acute, violent, and high contagious disease with a mortality rate of approximately 100% (1, 2). Recently, a new wave of African swine fever has broken out first in Georgia in 2007, and then quickly spread to many countries in Europe and Asia, including China in 2018 (3, 4). ASF is caused by African swine fever virus (ASFV), a large double-stranded DNA virus and the only member in the *Asfarviridae* family. The main target cells for ASFV are monocytes, macrophages and dendritic cells (5, 6). The virus genome varies between 170 and 190 kb and encodes 150 to 170 proteins that contribute not only to virus lifecycle but also to the evasion of host defenses (7–10). ASF outbreak is a disaster for swine industries in the world. Unfortunately, there are still no safe and effective vaccines for ASF. Thus, it is of great significance to study the pathogenesis of ASFV and dissect how ASFV evades host immune response.

The innate immune system is the host’s first line of defense against invading microbial pathogens. Host pattern recognition receptors (PRRs) recognize pathogen-associated molecular patterns (PAMPs) to activate host immune response (11). As a general cytosolic DNA sensor, cGAS recognizes viral dsDNA upon DNA virus infection and uses ATP and GTP to synthesize the second messenger cGAMPs (12). cGAMPs bind to the adaptor protein stimulator of interferon genes (STING) on the endoplasmic reticulum (ER), which becomes the platform to recruit TANK binding kinase1 (TBK1) (13). Subsequently, TBK1 phosphorylates STING and IFN regulatory factor 3 (IRF3) (14). Besides, STING also activates nuclear factor κB (NF-κB) (15, 16). The activated IRF3 and NF-κB translocate to the cell nucleus where they initiate the transcription of type I *IFNs* and other antiviral effector genes (16, 17). TBK1 is a crucial kinase to regulate innate immune signal transduction and its activity is tightly controlled. The activation of TBK1 is regulated in a variety of ways, including protein ubiquitination and phosphorylation. It has been reported that E3 ligases MIB1, MIB2 (18), RNF-128 (19), and TRAFs (20) promote the K63-linked polyubiquitination of TBK1 to enhance its functions (21). TNF receptor–associated factor 6 (TRAF6) as an adaptor protein participates in the regulation of cellular immunity in a variety of signaling pathways (22, 23). It has also been reported that E3 ligase TRAF6 heightens TBK1 activity in RIG-MAVS signaling pathway (20) and enhances NF-κB-mediated cytokine production (17, 23).

Previous studies have indicated that the ASFV Armenian/07 strain dramatically suppresses type I IFN production by modulating the cGAS-STING pathway (24). It is reported that the ASFV protein DP96R inhibits cGAS/STING/TBK1 signaling pathway by inhibiting TBK1 function (25). ASFV pMGF-505-7R (26) and pE120R (27) are reported to inhibit cGAS-STING pathway by targeting STING and IRF3, respectively. In addition, ASFV pI215R is shown to recruit E3 ubiquitin ligase RNF138 to degrade RNF128, thus negatively regulating the activation of TBK1 in an indirect way (28). However, there are still many proteins left with unknown functions. Thus, we want to explore whether other proteins help ASFV escape from host immunity (29).

ASFV pA151R, a non-structural protein, is identified as a thioredoxin and essential for the virus replication and morphogenesis (30–32). Knockdown of *A151R* by using siRNA interference significantly impaired ASFV replication (32). Recently, there is a report showing that deletion of A151R in ASFV causes a decreased replication rate and a drastic reduction in virus virulence in pigs (33). We also screened 36 ASFV genes and found that *A151R* played a role in the inhibition of IFN-β promoter activation (data not shown). Therefore, we hypothesize that pA151R might affects ASFV replication by down-regulating host antiviral response.

In this study, we found that ASFV pA151R strongly inhibited IFN-β production induced by cGAS-STING signaling pathway. We showed that pA151R suppressed TBK1 K63-linked polyubiquitination and phosphorylation via degrading E3 ligase TRAF6. In addition, we verified that the amino acids at sites H102, C109, C132 and C135 in pA151R were crucial for its inhibitory activity on type I IFN production. Importantly, we demonstrated that knockdown of pA151R by siRNA increased type I IFN production and down-regulated ASFV replication in porcine alveolar macrophages (PAMs). These findings might help us explain how ASFV escapes host antiviral immune responses.

## 2 Results

### 2.1 ASFV pA151R suppresses type I IFN production by inhibiting cGAS-STING signaling pathway

**Figure 1:**
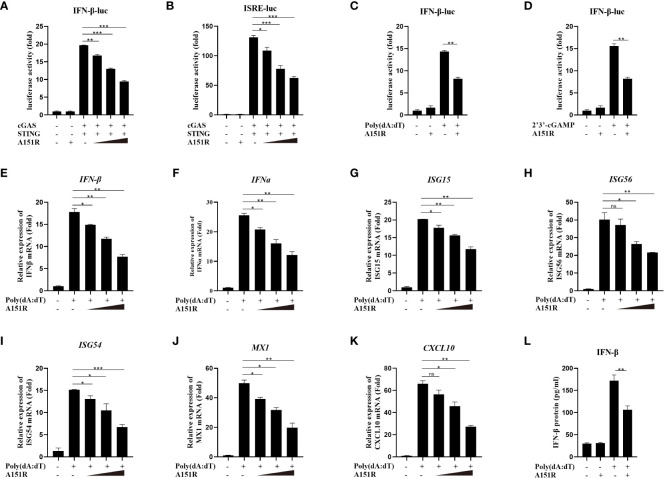
ASFV pA151R negatively regulates type I IFN production induced by cGAS-STING. **(A, B)** HEK-293T cells cultured in 24-well plates were transfected with IFNβ (40 ng) **(A)** or ISRE (40 ng) **(B)** luciferase (Luc) reporter and pRL-TK (4 ng), Flag-cGAS (20 ng) and Flag-STING (80 ng), and different dose HA-A151R (0, 100, 200, 400 ng) for 24 h followed by Dual-Luciferase assay. **(C, D)** CRL-2843 cells cultured in 24-well plates were transfected with IFNβ-Luc (40 ng), pRL-TK (4 ng), and empty vector or HA-A151R (400 ng). At 24 hours post transfection (hpt), cells were treated with poly(dA:dT) (1 μg/ml), or 2’3’-cGAMP (1 μg/ml) for 12 (h) The promoter activation of IFN-β was evaluated using the Dual-Luciferase assay. **(E-K)** CRL-2843 cells cultured in 24-well plates were transfected with different doses of HA-A151R (0, 100, 200, 400 ng). At 24 hpt, cells were treated with poly(dA:dT) (1 μg/ml) for 12 h, and the mRNA levels of *IFN-β*, *IFNα*, *ISG15*, *ISG56*, *ISG54*, *CXCL10*, and *MX1* were detected by qRT-PCR assay. **(L)** Transfection experiments were performed as described in panel **(E)** The supernatants were collected, and the amount of secreted IFN-β protein was measured by using an ELISA kit. Error bars denote standard errors of the means. All the data were analyzed using Student’s *t* test: *, *P* < 0.05; **, *P* < 0.01; ***, *P* < 0.001.

To investigate whether ASFV pA151R modulates type I interferon (IFN) production, we first explored its effects on cGAS-STING-mediated signaling pathway. HEK-293T cells were transfected with IFN-β-luciferase (IFN-β-Luc) reporter or ISRE-Luc reporter and pRL-TK, Flag-cGAS and Flag-STING, and HA-A151R at different dose. At 24 hours post transfection (hpt), the IFN-β and ISRE promoter activities were determined, respectively. As shown in **Figures 1A, B**, ectopic expression of pA151R significantly inhibited cGAS and STING-mediated IFN-β and ISRE promoter activities in a dose-dependent manner. Furthermore, we also analyzed whether pA151R had effects on IL-6-Luc. The results showed that pA151R did not affect IL-6 promoter activities (**Supplementary Figure 1A**). To further confirm its inhibitory effect on IFN-β promoter, pA151R was co-transfected into CRL-2843 cells (a porcine alveolar macrophages cell line) with IFN-β Luc and pRL-TK. At 24 hpt, the cells were treated with the synthetic double-stranded DNA (dsDNA)-mimetic poly(dA:dT) or STING specific agonist 2’3’-cGAMP for 12 h. Cells were then harvested to analyze IFN-β promoter activation. As shown in **Figures 1C, D**, pA151R inhibited IFN-β promoter activation induced by poly(dA:dT) and 2’3’-cGAMP, respectively. To investigate whether pA151R affects the expression of antiviral genes in cells induced by cGAS-STING signaling pathway, CRL-2843 cells were transfected with HA-vector or different dose of HA-A151R. At 24 h later, we stimulated cells with poly(dA:dT) for 12 h and then analyzed *IFN* and *ISGs* expressions by qRT-PCR. The results showed that pA151R significantly inhibited poly(dA:dT)-induced *IFNβ*, *IFNα*, *ISG15*, *ISG56*, *ISG54*, *CXCL10*, and *MX1* expressions in a dose-dependent manner (**Figures 1E–K**), but had no effects on *TBK1* expression (an unrelated control) (**Supplementary Figure 1B**). Meanwhile, we also analyzed IFNβ protein level in supernatants using ELISA. As shown in **Figure 1L**, pA151R also significantly inhibited the secretion of IFN-β. Taken together, these results suggest that ASFV pA151R inhibits cGAS-STING-mediated IFN-β production.

### 2.2 ASFV pA151R targets TBK1 to suppress type I IFN production

**Figure 2:**
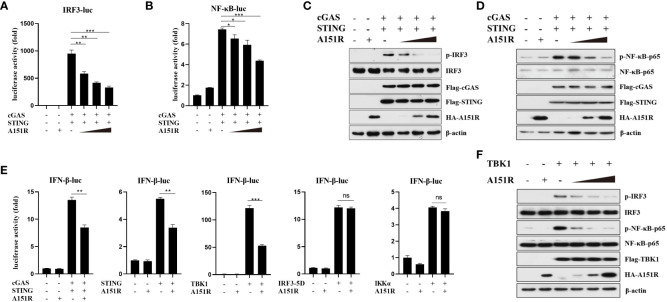
ASFV pA151R targets TBK1 to suppress type I IFN production. **(A, B)** HEK-293T cells cultured in 24-well plates were transfected with IRF3-luc (40 ng) **(A)** or NF-κB-luc (40 ng) **(B)** and pRL-TK (4 ng), Flag-cGAS (20 ng) and Flag-STING (80 ng), and different dose of HA-A151R (0, 100, 200, 400 ng) for 24 h followed by Dual-Luciferase assay. **(C, D)** HEK-293T cells cultured in 6-well plates were transfected with Flag-cGAS (200 ng) and Flag-STING (400 ng), and different dose of HA-A151R (0, 0.5, 1.0, 2.0 μg) for 24 h and the IRF3 **(C)** and NF-κB **(D)** phosphorylation levels were detected by western blot. **(E)** HEK-293T cells were transfected with IFN-β-Luc (40 ng) and pRL-TK (4 ng), a plasmid expressing cGAS (20 ng), STING (80 ng), TBK1 (20 ng), IRF3 (80 ng), IRF3-5D (20 ng), or IKKα (20 ng), and empty vector or HA-A151R (200 ng). At 24 hpt, the promoter activation of IFN-β was evaluated through the Dual-Luciferase assay. **(F)** The transfection experiments and western blot assay were performed as described in panels C and D except that Flag-TBK1 expression plasmid was used instead of Flag-cGAS and Flag STING expression plasmids. Error bars denote standard errors of the means. All the data were analyzed using Student’s *t* test: *, *P* < 0.05; **, *P* < 0.01; ***, *P* < 0.001.

To elucidate the specific mechanisms by which pA151R regulates IFN-β production, we co-transfected IFN-β promoter containing the transcription factor IRF3 or NF-κB binding site and pRL-TK into HEK-293T cells with Flag-cGAS, Flag-STING and different dose of HA-A151R. At 24 hpt, cells were harvested for the luciferase activity analysis. As showed in **Figures 2A, B**, pA151R significantly inhibited the activation of the promoter either containing IRF3 or NF-κB binding site. Next, we analyzed the effects of pA151R on the activation of IRF3 and NF-κB. HEK-293T cells were co-transfected with Flag-cGAS, Flag-STING, and HA-Vector or different dose of HA-A151R for 24 h. And then, we analyzed the phosphorylation of IRF3 and NF-κB by western blot. The results showed that pA151R reduced the phosphorylation levels of IRF3 and NF-κB induced by cGAS and STING in a dose-dependent manner (**Figures 2C, D**). These results indicate that pA151R interferers with both STING-TBK1-IRF3 axis and STING-TBK1-NF-κB axis.

To find the target of ASFV pA151R in cGAS-STING signaling pathway, we assessed the effect of pA151R on IFN-β promoter activation induced by the crucial signal components. IFN-β-Luc reporter and pRL-TK were co-transfected into HEK-293T cells with cGAS, STING, TBK1, IKKα, or IRF3-5D (constitutively active IRF3), and HA-vector or HA-A151R. At 24 hpt, cells were harvested for luciferase activity analysis. As shown in **Figure 2E**, pA151R significantly impeded IFN-β promoter activities induced by cGAS, STING, or TBK1, but not IKKα or IRF3-5D, implying that pA151R might target TBK1. To confirm this observation, HEK-293T cells were co-transfected with Flag-TBK1 and different dose of HA-A151R. At 24 h later, cells were harvested to analyze IRF3 and NF-κB phosphorylation. As shown in **Figure 2F**, pA151R inhibited TBK1-mediated IRF3 and NF-κB phosphorylation in a dose-dependent manner. Taken together, our results suggest that ASFV pA151R suppresses IFN-β production by interfering with TBK1.

### 2.3 ASFV pA151R inhibits TBK1 K63-linked polyubiquitination and phosphorylation

**Figure 3:**
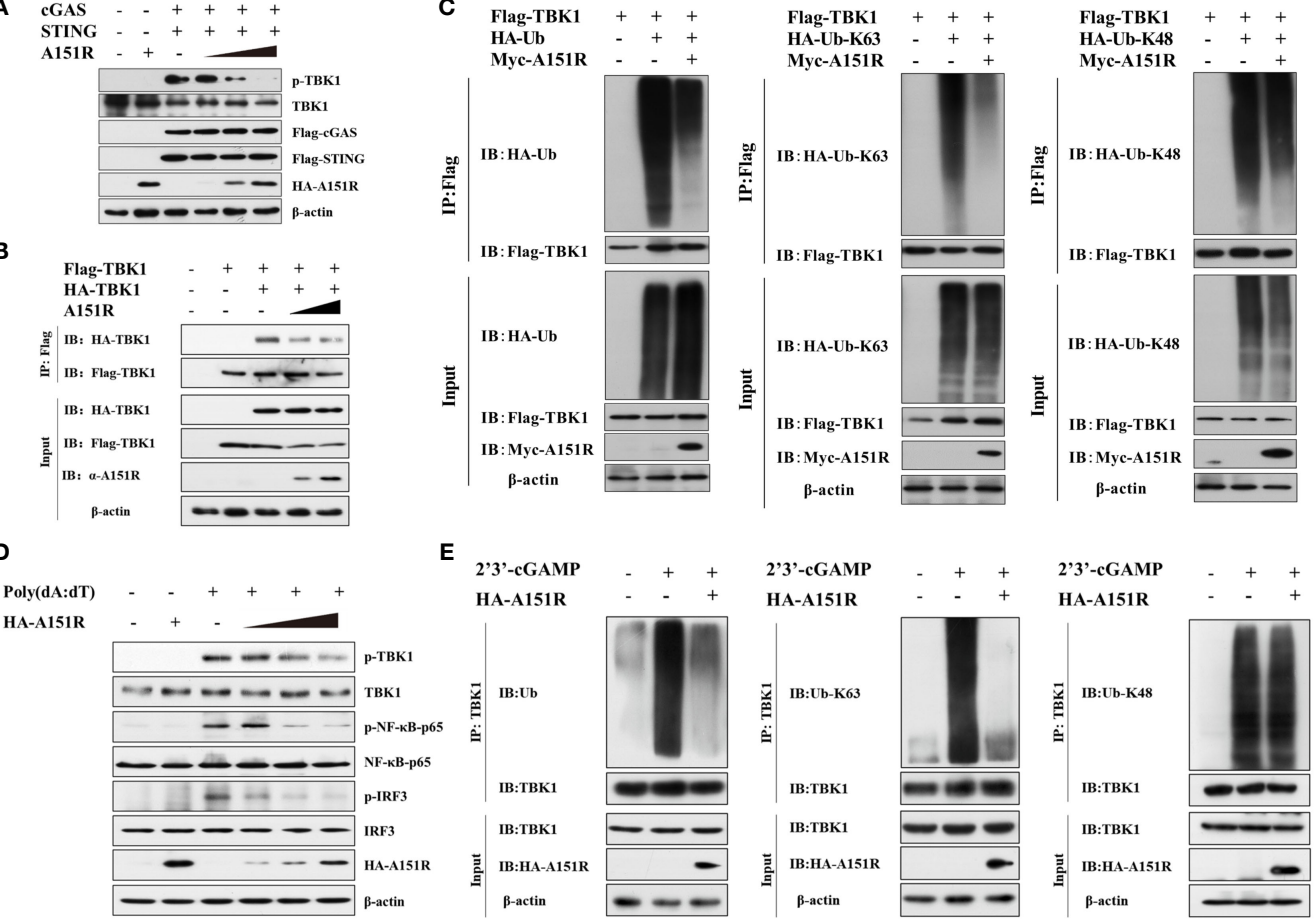
ASFV pA151R inhibits TBK1 K63-linked polyubiquitination and phosphorylation. **(A)** HEK-293T cells cultured in 6-well plates were transfected with Flag-cGAS (200 ng) and Flag-STING (400 ng), and different doses of HA-A151R (0, 0.5, 1.0, 2.0 μg) for 24 h and TBK1 phosphorylation was analyzed by western blot. **(B)** HEK-293T cells cultured in 6-well plates were transfected with Flag-TBK1 and HA-TBK1, and empty vector or increased dose of HA-A151R. At 24 hpt, the cell lysates were immunoprecipitated with anti-Flag (M2) beads and TBK1 dimerization was analyzed using the indicated antibodies. **(C)** Flag-TBK1, and HA-ubiquitin (Ub), HA-Ub-K63, or HA-Ub-K48 were co-transfected with or without Myc-A151R into HEK-293T cells cultured in 6-well plates for 24 (h) The cell lysates were immunoprecipitated with anti-Flag (M2) beads for the detection of TBK1 ubiquitination by using the indicated antibodies. **(D)** CRL-2843 cells cultured in 6-well plates were transfected with different dose of HA-A151R (0, 0.5, 1.0, 2.0 μg) for 24 h and then the cells were treated with poly(dA:dT) (1 μg/ml). At 12 h later, the phosphorylation levels of TBK1, IRF3 and NF-κB were analyzed by western blot using the indicated antibodies. **(E)** Transfection experiments were performed as described in panel D except that 2’3’-cGAMP was used instead of poly(dA:dT). The cell lysates were immunoprecipitated with anti-TBK1 beads for ubiquitination assay by the indicated antibodies.

TBK1 plays a central role in innate immunity. To explore the specific impact of pA151R on the activation of TBK1, we investigated whether pA151R interfered with the post-translational modification of TBK1 in cGAS-STING signaling pathway. HEK-293T cells were transfected with different dose of HA-A151R, Flag-cGAS, and Flag-STING. At 24 hpt, cells were harvested for the analysis of TBK1 phosphorylation. As shown in **Figure 3A**, pA151R inhibited cGAS-STING-mediated TBK1 phosphorylation in a dose-dependent manner. Since TBK1 dimerization is essential for its activation (34), we then tested whether pA151R affected TBK1 dimerization. HEK-293T were co-transfected with Flag-TBK1, HA-TBK1, and different dose of HA-A151R. At 24 hpt, cell lysates were incubated with Flag (M2) beads, and the dimerization of TBK1 was then detected using antibody against HA. As shown in **Figure 3B**, pA151R significantly inhibited TBK1 aggregation. As previously described, K63-linked polyubiquitination is required for TBK1 activation (21, 34), we next investigated whether pA151R modulated TBK1 ubiquitination. HEK-293T cells were transfected with HA-ubiquitin, Flag-TBK1 and Myc-A151R. At 24 hpt, cells were harvested and lysed. Then, TBK1 was pulled down using anti-Flag (M2) beads and its ubiquitin level was analyzed by western blot using antibody against HA. As shown in **Figure 3C**, pA151R dramatically reduced the total ubiquitination of TBK1. In addition, to clarify whether pA151R specifically affects has effect TBK1 ubiquitination, we analyzed Flag-STING ubiquitination in the same condition as Flag-TBK1. Our results showed that pA151R had no obvious effect on STING ubiquitination (**Supplementary Figure 2A**). To further determine which type of TBK1 ubiquitination is impacted by pA151R, HEK-293T cells were co-transfected with HA-K63-linked-ubiquitin or HA-K48-linked-ubiquitin, together with Flag-TBK1 and Myc-A151R. At 24 hpt, cells were harvested and incubated with Flag (M2) beads, and its ubiquitin level was analyzed by using antibody against HA. Our results showed that pA151R only influenced K63-linked polyubiquitination of TBK1 but not K48-linked polyubiquitination of TBK1 (**Figure 3C**). To further confirm the results in porcine cells, we used CRL-2843 cells to repeat the experiment. CRL-2843 cells were transfected with different doses of HA-A151R for 24 h, and then treated with poly(dA:dT). At 12 h after treatment, cells were harvested for western blot analysis. As shown in **Figure 3D**, pA151R obviously inhibited the phosphorylation of TBK1, IRF3 and NF-κB in a dose-dependent manner. To further confirm the effects of pA151R on TBK1 ubiquitination, we transfected CRL-2843 cells with HA-A151R for 24 h, and then added 2’3’-cGAMP to the cells. At 12 h later, cell lysates were incubated with anti-TBK1 beads, and then the washed beads were assessed for ubiquitin analysis. Consistent with the previous results, pA151R significantly inhibited TBK1 K63-linked polyubiquitination (**Figure 3E**, middle), but did not impact TBK1 K48-linked polyubiquitination (**Figure 3E**, right). Taken together, these data suggest pA151R inhibits type I IFN production by interfering with TBK1 K63-linked polyubiquitination and phosphorylation.

### 2.4 ASFV pA151R interferes with TBK1 activation in cGAS-STING signaling pathway via interacting with TRAF6

**Figure 4:**
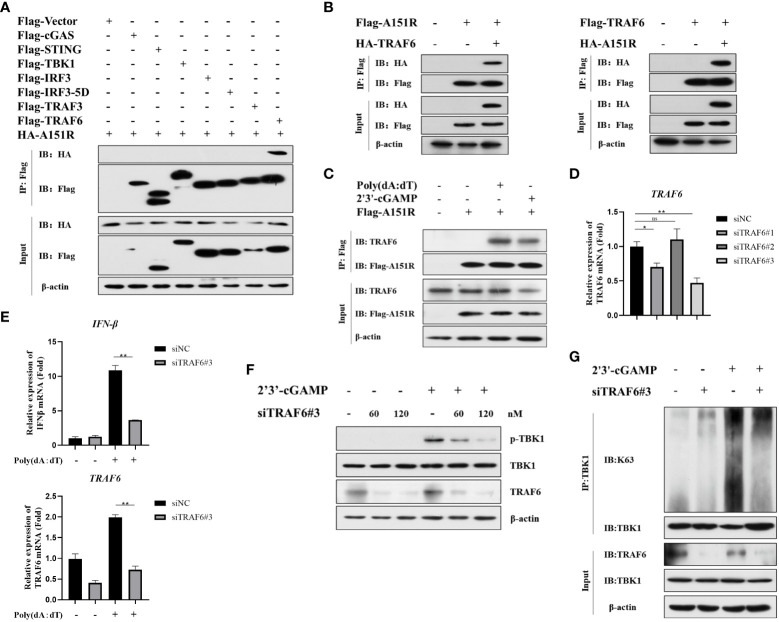
ASFV pA151R interferes with TBK1 activation in cGAS-STING signaling pathway via interacting with TRAF6. **(A)** HEK-293T cells were co-transfected with a Flag-tag plasmid expressing cGAS, STING, TBK1, IRF3, IRF3-5D, TRAF3, or TRAF6, and HA-A151R. At 24 hpt, the cells lysates were immunoprecipitated with anti-Flag (M2) beads and examined by western blot using the indicated antibodies. **(B)** HEK-293T cells were transfected with Flag-TRAF6 and HA-A151R, or Flag-A151R and HA-TRAF6 for 24 **(h)** The cell lysates incubated with anti-Flag (M2) beads and then the interaction of A151R and TRAF6 was detected using the indicated antibodies. **(C)** CRL-2843 cells were transfected with empty vector or Flag-A151R. At 24 hpt, the cells were treated with poly(dA:dT) (lane 3) or 2’3’-cGAMP (lane 4) for 12 (h) The interaction of A151R and endogenous TRAF6 were analyzed by co-immunoprecipitation and immunoblotting. **(D)** CRL-2843 cells were transfected with control siRNA (NC) or each of the three siTRAF6s for 24 h and then the mRNA level of *TRAF6* expression was analyzed by qRT-PCR. **(E-G)** CRL-2843 cells were transfected with control siRNA (NC) or siTRAF6-#3. At 24 hpt, the cells were treated with poly(dA:dT) or 2’3’-cGAMP for 12 h and then cells were harvested and lysed. The mRNA level of IFNβ were analyzed by qRT-PCR **(E)**, TBK1 phosphorylation analysis was examined by western blot **(F)**, and TBK1 ubiquitination was detected by co-immunoprecipitation and immunoblotting **(G)**.

It has been reported that E3 ligase (TRAF3 and TRAF6) is crucial for TBK1 activation in different signal pathways (15, 20, 21, 35). To further ascertain the target signaling molecules in cGAS-STING signaling pathway, we co-transfected HA-A151R into HEK-293T cells with Flag-tagged- cGAS, STING, TBK1, IRF3, IRF3-5D, TRAF3, or TRAF6. At 24 h later, cell lysates were incubated with anti-Flag (M2) beads, and then co-immunoprecipitation (co-IP) analysis was performed by using antibody against HA. As shown in **Figure 4A**, the co-IP results showed that pA151R interacted with TRAF6 but not TBK1 or other molecules in cGAS-STING signal pathway. To further confirm the interaction of pA151R and TRAF6, we did co-IP again. As shown in **Figure 4B**, our data indicated that pA151R interacted with TRAF6 whenever we used pA151R to pull down TRAF6 or used TRAF6 to pull down pA151R. Then we investigated whether pA151R interacted with endogenic TRAF6. CRL-2843 cells were transfected with Flag-Vector or Flag-A151R for 24 h, and then cells were treated with poly(dA:dT) or 2’3’-cGAMP. At 12 h after treatment, cell lysates were incubated with Flag (M2) beads to accumulate Flag-A151R, and then the endogenous TRAF6 was examined by western blot. Our results showed that pA151R interacted with TRAF6 in cells during STING signaling pathway activation, but not in resting state cells (**Figure 4C**).

It has been reported that TRAF6 is an important immune modulatory factor in diverse signal pathways and available for TBK1 activation (22, 36). Mitochondrial antiviral-signaling protein (MAVS) transmits signals from RIG-I-like receptors after RNA virus infections and then recruits TRAF6 to synergistically induce host antiviral innate immune responses (20). Besides, TRAF6 is also recruited by the STING complex to activate NF-κB (15). However, whether TRAF6 facilitates TBK1phosphorylation and ubiquitylation in cGAS-STING signaling pathway is unclear. To explore whether TRAF6 functions in cGAS-STING pathway, we first synthesized 3 siRNAs targeting porcine TRAF6 and found that siTRAF6#3 was the most efficient one to knockdown TRAF6 (**Figure 4D**; **Table 1**). CRL-2843 cells were transfected with siTRAF6#3 for 24 h and then treated with poly(dA:dT). At 12 h later, cells were harvested for the analysis of *IFNβ* expression using qRT-PCR. The results showed that knockdown of TRAF6 significantly inhibited *IFNβ* expression (**Figure 4E**). To further clarify the role of TRAF6 in STING complex, CRL-2843 cells were transfected with different dose of siTRAF6#3 for 24 h and then treated with 2’3’-cGAMP. At 12 h later, cells were harvested and lysed for the analysis of TBK1 phosphorylation and ubiquitination. As shown in **Figures 4F, G**, knockdown of TRAF6 significantly inhibited TBK1 phosphorylation and K63-linked polyubiquitination activation. Taken together, our data demonstrate that E3 ligase TRAF6 is crucial for cGAS-STING-mediated IFN-β production via facilitating K63-linked ubiquitination of TBK1.

**Table 1:** siRNAs targeting ASFV A151R and Swine TRAF6 used in this study.

siRNA	Sense (5’→3’)	Antisense (5’→3’)
siNC	UUCUCCGAACGUGUCACGUTT	ACGUGACACGUUCGGAGAATT
siA151R-1	CGGUCCUCAUAUCUUUAAUTT	AUUAAAGAUAUGAGGACCGTT
siA151R-2	GCCGCGUACUCAAAUUUAUTT	AUAAAUUUGAGUACGCGGCTT
siA151R-3	GAAGAUCUUAAAGGAGCAATT	UUGCUCCUUUAAGAUCUUCTT
siA151R-4	GUCGCCCAAUAUAUUCCAATT	UUGGAAUAUAUUGGGCGACTT
siTRAF6-1	GCAUCUUGAGGAUCAUCAATT	UUGAUGAUCCUCAAGAUGCTT
siTRAF6-2	GCAGUUAGAAGGUCGCCUUTT	AAGGCGACCUUCUAACUGCTT
siTRAF6-3	GGAACGAUACGCCUUACAATT	UUGUAAGGCGUAUCGUUCCTT

### 2.5 ASVF pA151R interferes with the interaction between TRAF6 and TBK1

**Figure 5:**
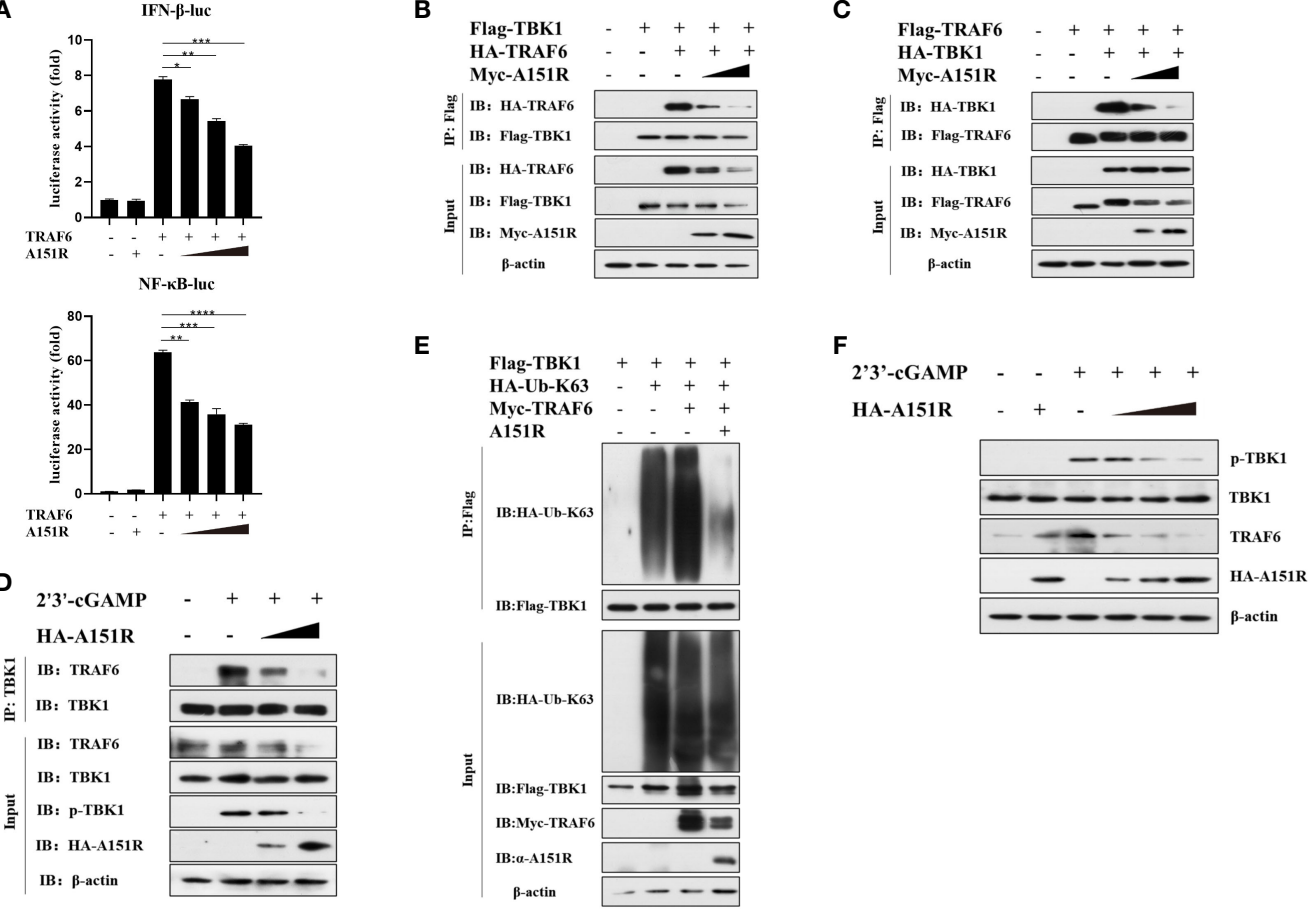
ASVF pA151R interferes with the interaction between TRAF6 and TBK1. **(A)** HEK-293T cells cultured in 24-well plates were co-transfected with IFNβ-Luc (40 ng) or NF-κB-luc (40 ng), and pRL-TK (4 ng), together with Flag-TRAF6 (100 ng) and different dose of HA-A151R (0, 100, 200, 400 ng) for 24 h followed by Dual-Luciferase assay. **(B, C)** HEK-293T cells cultured in 6-well plates were co-transfected with Flag-TBK1 and HA-TRAF6, or Flag-TRAF6 and HA-TBK1, and empty vector or increased doses of HA-A151R for 24 h. The cell lysates were immunoprecipitated with anti-Flag (M2) beads and the interaction of TBK1 and TRAF6 was detected using the indicated antibodies. **(D)** CRL-2843 cells cultured in 6-well plates were transfected with empty vector or increased doses of HA-A151R for 24 h and then treated with 2’3’-cGAMP. At 12 later, the cell lysates were immunoprecipitated with anti-TBK1 beads and the interaction of TBK1 and TRAF6 was detected by using the indicated antibodies. **(E)** HEK-293T cells cultured in 6-well plates were co-transfected with Flag-TBK1, HA-Ub-K63 and Myc-TRAF6, together with a plasmid expressing A151R. At 24 hpt, cell lysates were immunoprecipitated with anti-Flag (M2) beads for TBK1 ubiquitination assay by using the indicated antibodies. **(F)** Transfection experiments were performed as described in panel D. The cells were collected and lysed for the detections of TRAF6 expression in protein level and TBK1 phosphorylation by western blot using the indicated antibodies. Error bars denote standard errors of the means. All the data were analyzed using Student’s *t* test: *, *P* < 0.05; **, *P* < 0.01; ***, *P* < 0.001; ****, *P* < 0.0001.

To further investigate how pA151R interferes with the function of TRAF6, we transfected HEK-293T cells with IFN-β-Luc or NF-κB-Luc and pRL-TK, Flag-TRAF6, and different dose of HA-A151R. At 24 hpt, cells were harvested for the analysis of luciferase activities. The luciferase assays showed that pA151R significantly inhibited TRAF6-induced IFN-β and NF-κB promoter activities in a doses-dependent manner (**Figure 5A**). It is well known that the C-terminal tail of STING is the crucial domain for STING to recruit TBK1 and TRAF6 (15, 37, 38). Thus, we next examined whether pA151R disrupted TBK1 and TRAF6 interaction in the STING complex. Flag-TBK1 and HA-TRAF6 or Flag-TRAF6 and HA-TBK1 were co-transfected with increased doses of Myc-A151R into HEK-293T cells. At 24 hpt, cells were harvested and lysed for co-IP analysis. As shown in **Figures 5B, C**, pA151R impeded the interaction between TRAF6 and TBK1. To verify that pA151R disrupts TRAF6 and TBK1 interaction, we ectopically expressed pA151R in CRL-2843 cells for 24 h and then treated cells with 2’3’-cGAMP. At 12 h later, cell lysates were immunoprecipitated with anti-TBK1 beads and analyzed by western blot using antibody against TRAF6. Our results showed that TBK1 interacted with TRAF6 during STING activation. However, when pA151R was present, the interaction of TBK1-TRAF6 was dramatically inhibited (**Figure 5D**). To further clarify the specific relationship among TBK1, TRAF6 and pA151R, HEK-293T were co-transfected Flag-TBK1, Myc-TRAF6, HA-K63-ubiquitin, and A151R. At 24 hpt, we examined the K63-linked polyubiquitylation of TBK1. As shown in **Figure 5E**, TRAF6 facilitated TBK1 ubiquitylation, but this phenomenon no longer existed when pA151R was present. Interestingly, we also observed that pA151R reduced TRAF6 expression in protein level when cells were activated (**Figures 5B–E**). To confirm this observation, we transfected CRL-2843 cells with different dose of HA-A151R for 24 h, and then treated the cells with 2’3’-cGAMP. At 12 h after treatment, cells were harvested for western blot analysis. Our results showed that pA151R not only inhibited TBK1 phosphorylation but also degraded TRAF6 in a doses-dependent manner (**Figure 5F**). Taken together, our data suggest that pA151R interferes with TBK1-TRAF6 interaction by down-regulating TRAF6.

### 2.6 ASFV pA151R degrades TRAF6 through apoptosis pathway

**Figure 6:**
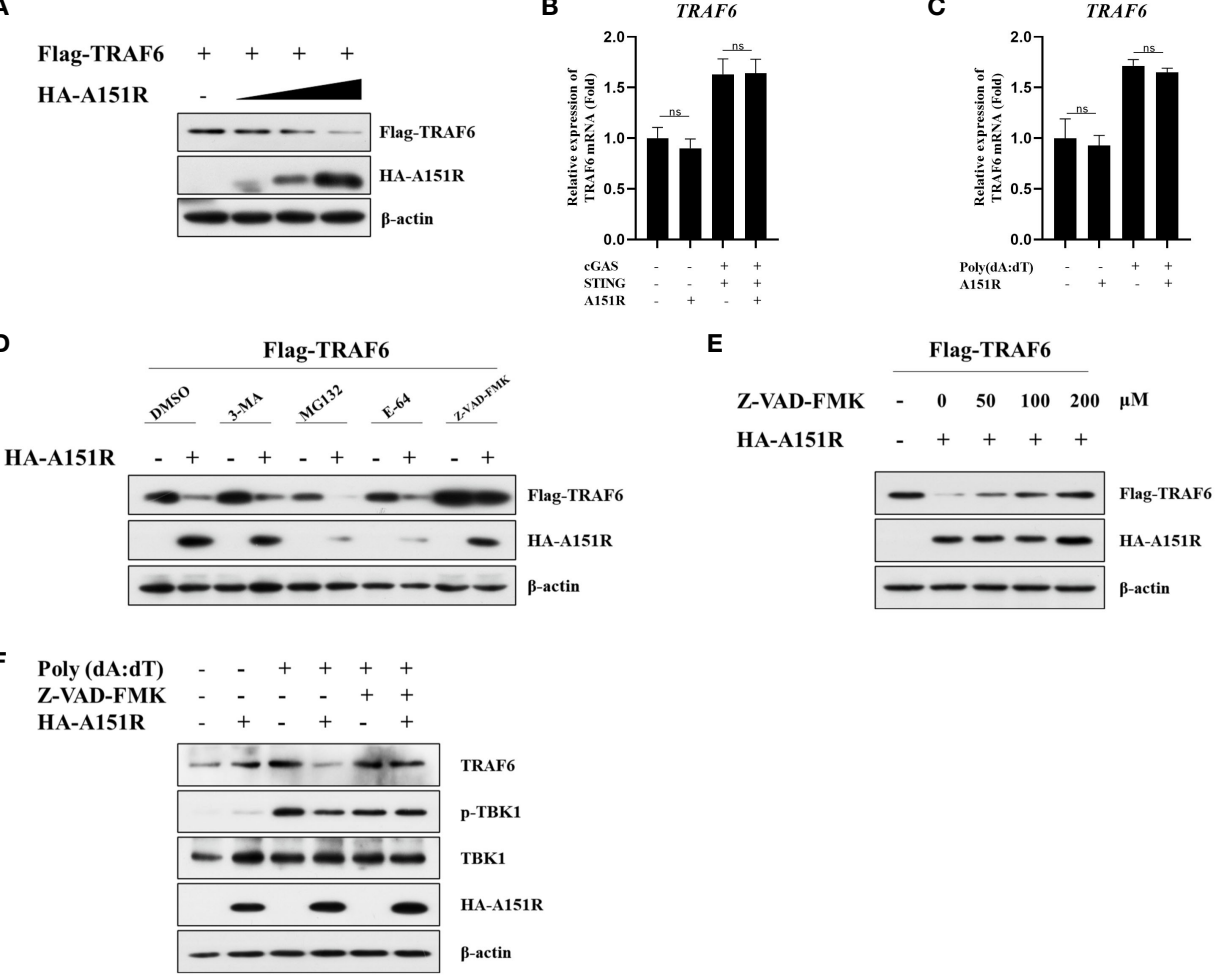
ASFV pA151R degrades TRAF6 through apoptosis pathway. **(A)** HEK-293T cells cultured in 6-well plates were co-transfected with Flag-TRAF6 (500 ng) and different dose of HA-A151R (0, 0.5, 1.0, 2.0 μg) for 24 (h) TRAF6 expression was analyzed by western blot. **(B)** HEK-293T cells cultured in 24-well plates were transfected with Flag-cGAS (20 ng) and Flag-STING (80 ng), and empty vector or HA-A151R (200 ng) for 24 h and the mRNA level of *TRAF6* was quantified by qRT-PCR assay. **(C)** CRL-2843 cells cultured in 24-well plates were transfected empty vector or HA-A151R (200 ng) for 24 h and then were treated with poly(dA:dT) for another 12 (h) The cells were detected for the *TRAF6* expression in mRNA level. **(D)** HEK-293T cells cultured in 6-well plates were co-transfected with Flag-TRAF6 (500 ng) and empty vector or HA-A151R (2 μg). At 18 hpt, the cells were treated with 3-MA (5 mM), MG132 (20 μM), E-64 (100 mM), or Z-VAD-FMK (100 μM) for 8 (h) The cell lysates were subjected to TRAF6 and A151R expression by western blot. **(E)** Transfection experiments were performed as described in panel **(C)** At 18 hpt, the cells were treated with different doses (0, 50, 100, 200 μM) of Z-VAD-FMK for 8 (h) The expression of TRAF6 and A151R were analyzed by western blot. **(F)** CRL-2843 cells cultured in 6-well plates were transfected empty vector or HA-A151R (2 μg) for 24 h and then were treated with poly(dA:dT) (1 μg/ml) and Z-VAD-FMK (200 μM) for another 12 (h) The cells were harvested to analyze TRAF6 expression and TBK1 phosphorylation by western blot. Error bars denote standard errors of the means. All the data were analyzed using Student’s *t* test: *, *P* < 0.05; **, *P* < 0.01; ***, *P* < 0.001.

As we observed that pA151R down-regulated TRAF6, we then investigated the effects of pA151R on the transcription and translation of TRAF6. HEK-293T cells were transfected with Flag-TRAF6 and different dose of HA-A151R. At 24 hpt, cells were harvested to analyze TRAF6 expression. Western blot analysis showed that overexpression of pA151R significantly reduced TRAF6 (**Figure 6A**). However, pA151R had no effects on *TRAF6* expression at mRNA level in both HEK-293T cells (**Figure 6B**) and CRL-2843 cells (**Figure 6C**). To illustrate how pA151R induces TRAF6 degradation, we transfected HEK-293T cells with Flag-TRAF6 and HA-A151R for 24 h, and then treated the cells with the autophagosome inhibitor 3-MA, the proteasome inhibitor MG-132, the lysosomal inhibitors E-64, and the apoptotic inhibitor Z-VAD –FMK, respectively. At 12 h after treatment, cells were harvested for western blot analysis. The results displayed that apoptotic inhibitor Z-VAD -FMK inhibited pA151R-mediated TRAF6 degradation (**Figure 6C**) in a doses-dependent manner (**Figure 6D**). These results were also confirmed in porcine CRL-2843 cells (**Figure 6F**). Taken together, our results indicate that pA151R degrades TRAF6 via the apoptosis pathway.

### 2.7 H102, C109, C132, C135 are the essential amino acids for pA151R to maintain its stability and preform its function

**Figure 7:**
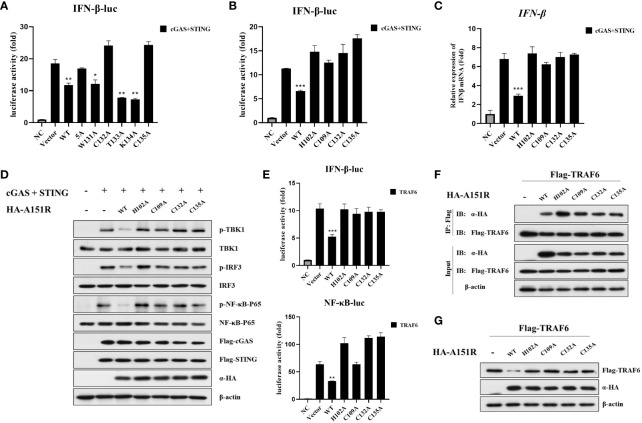
H102, C109, C132, C135 are the essential amino acids for pA151R to maintain its stability and preform its function**. (A, B)** HEK-293T cells cultured in 24-well plates were co-transfected with IFNβ-Luc (40 ng), pRL-TK (4 ng), Flag-cGAS (20 ng) and Flag-STING (80 ng), and HA-A151R or HA-A151R mutants for 24 h followed by Dual-Luciferase assay. **(C, D)** HEK-293T cells were co-transfected with Flag-cGAS and Flag-STING, together with HA-A151R or HA-A151R mutants. At 24 hpt, the cells were collected and lysed. *IFN-β* expression were analyzed by qRT-PCR **(C)**, and the phosphorylation of TBK1, IRF3 and NF-κB were detected by western blot using the indicated antibodies **(D)**. **(E–G)** HEK-293T cells were transfected with Flag-TRAF6, and HA-A151R or each of the HA-A151R mutants for 24 (h) Effects of HA-A151R or its mutants on TRAF6-triggered IFN-β and NF-κB promoter activation were examined by Dual-Luciferase assay **(E)**. The interaction of TRAF6 and HA-A151R or its mutants were detected by co-immunoprecipitation and immunoblotting **(F)**. The influence of HA-A151R or its mutants on TRAF6 expression in a protein level were detected by western blot. Error bars denote standard errors of the means. All the data were analyzed using Student’s *t* test: *, *P* < 0.05; **, *P* < 0.01; ***, *P* < 0.001.

It has been reported that pA151R has a thioredoxin active site feature, the WCTKC motif (39). Based on these data, we constructed five pA151R single-site mutants, including W131A, C132A, T133A, K134A, and C135A, and a five-site mutant (WCTKC to AAAAA). To explore whether these mutants have effects on cGAS-STING signaling, we transfected HEK-293T cells with IFN-β- Luc reporter and pRL-TK, Flag-cGAS and Flag-STING, and HA-A151R or each of the six mutant HA-A151R. At 24 hpt, cells were collected for luciferase assay analysis. As shown in **Figure 7A**, the mutation of amino acid 132 or 135 (but not the others) in pA151R abrogated its inhibitory activity on IFN-β promoter activation. According to the structure of pA151R, the three cysteines on 109, 132 and 135 sites together with the histidine on 102 could coordinate a Zn^2+^ ion to form a Zn-binding motif, and these four amino acids are indispensable for the stabilization of this partial construction (39). Then, we constructed another two pA151R mutants (H102A, C109A). To test if these two mutants affect the ability of pA151R to inhibit cGAS-STING signaling, we transfected each of these two mutants into HEK-293T cells with IFN-β- Luc reporter and pRL-TK, Flag-cGAS, and Flag-STING. The luciferase assay showed that the two mutants (H102A and C109A) lost the ability to inhibit the activation of IFN-β promoter mediated by cGAS and STING (**Figure 7B**). To further test whether the four mutants (H102A, C109A, C132A, C135A) affect *IFN-β* expression, HEK-293T cells were co-transfected with Flag-cGAS, Flag-STING and wild type pA151R or each of the four pA151R mutants. At 24 hpt, cells were collected for qRT-PCR analysis. As shown in **Figure 7C**, the four mutants (H102A, C109A, C132A, C135A) lost the ability to suppress cGAS-STING-meditated *IFNβ* expression (**Figure 7C**). To further explore the effects of the four pA151R mutants on cGAS-STING signaling, we transfected HEK-293T cells with wild type HA-A151R or each of the four HA-A151R mutants (H102A, C109A, C132A, C135A), Flag-cGAS, and Flag-STING. At 24 hpt, cells were harvested to analyze the phosphorylation of TBK1, IRF3, and NF-κB. As shown in **Figure 7D**, the four pA151R mutants (H102A, C109A, C132A, C135A) no longer inhibited the phosphorylation of TBK1, IRF3, and NF-κB. To investigate whether these mutations have effects on TRAF6, we transfected HEK-293T cells with IFN-β-Luc reporter or NF-κB-Luc reporter and pRL-TK, Flag-TRAF6, and wild type HA-A151R or each of the four HA-A151R mutants. At 24 h later, cells were harvested for luciferase assay analysis. As shown in **Figure 7E**, the four pA151R mutants no longer inhibited the activation of IFN-β and NF-κB promoter induced by TRAF6. To further explore the relationship between TRAF6 and each of the four pA151R mutants, we then investigated the interaction between them. HEK-293T cells were transfected with Flag-TRAF6 and each of the four pA151R mutants. At 24 hpt, cells were harvested for co-IP and immunoblotting. Interestingly, co-IP experiments indicated that all the four pA151R mutants still interacted with TRAF6 (**Figure 7F**). However, they lost the ability to degrade TRAF6 (**Figure 7G**). Taken together, H102, C109, C132, C135 are the essential amino acids for pA151R to inhibit the cGAS-STING mediated signaling pathway.

### 2.8 Knockdown of pA151R attenuates ASFV replication by up-regulating type I IFN production

**Figure 8:**
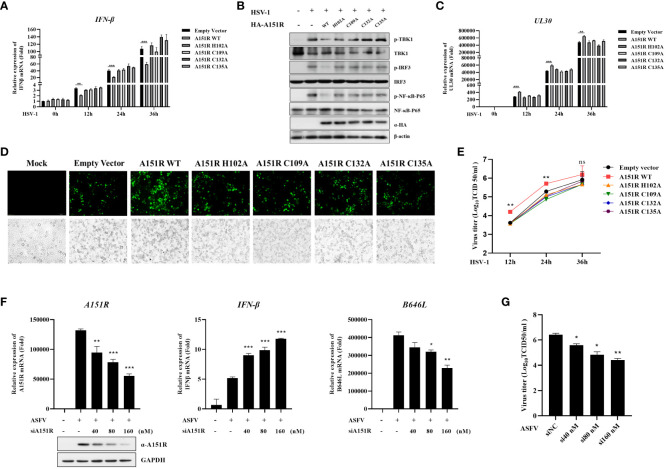
Knock-down of pA151R attenuates ASFV replication by up-regulating type I IFN production. **(A, C, E)** HeLa cells were transfected with HA-A151R (400 ng) or each of its mutants for 24 h and then infected with HSV-1 (MOI = 0.05). The cells were harvested at 12, 24, and 36 hours to analyze the mRNA levels of *IFN-β* **(A)** and *UL30* **(C)** by qRT-PCR assay. The viral titer in the supernatant from HSV-1 infected cells was analyzed by TCID_50_ assay **(E)**. **(B)** HeLa cells were transfected HA-A151R (2 μg) or each of its mutants for 24 h followed by infection with HSV-1 (MOI = 0.05). At 36 hpt, cells were lysed for western blot using the indicated antibodies. **(D)** Transfection and infection experiments were performed as described in panel B and then the virus replication in cells were analyzed by fluorescence microscope. **(F, G)** PAMs were transfected with different doses of mixed siRNAs (#1 and #4)) targeting the *A151R* gene for 24 h and then infected with ASFV at an MOI of 1. At 24 h postinfection (hpi), the expression of *A151R* in mRNA level and protein level were detected by qRT-PCR assay and western blot, respectively. The mRNA level of ASFV P72 (*B646L* gene) and *IFN-β* were examined by qRT-PCR assay **(F)**. The viral titer in the supernatant from ASFV infected cells was analyzed by using TCID_50_ assay **(G)**. All the data were analyzed using Student’s *t* test: *, *P* < 0.05; **, *P* < 0.01; ***, *P* < 0.001.

To explore whether pA151R and its mutants influence other DNA virus replication and type I IFN production, we transfected wild-type HA-A151R or each of the mutants (H102A, C109A, C132A, C135A) into HeLa cells. At 24 hpt, we infected HeLa cells with HSV-1 (MOI = 0.05). Then, we tested *IFN-β* expression and the phosphorylation of important signal components in the cGAS-STING signaling pathway using qRT-PCR and western blot, respectively. As shown in **Figure 8A**, the mutants of pA151R did not affect *IFN-β* mRNA expression in cells infected with HSV-1. As expected, the four pA151R mutants had no effects on the phosphorylation of TBK1, IRF3 and NF-κB (**Figure 8B**). Subsequently, we analyzed HSV-1 replication, and our results indicated that WT pA151R facilitated *UL30* expression (the DNA polymerase of HSV-1), but the four mutants had no effects on HSV-1 replication (**Figure 8C**). Fluorescence staining also showed that only WT pA151R facilitated HSV-1 replication (**Figure 8D**). In addition, we analyzed the dynamics of HSV-1 growth in HeLa cells transfected with pA151R or each of the mutants. As shown in **Figure 8E**, our data displayed that WT pA151R promoted HSV-1 replication at all the time points, whereas the mutants had no effect on HSV-1 replication. These results suggest ASFV pA151R promotes virus replication though disrupting type I IFN signaling pathway.

Finally, to investigate whether knockdown of pA151R influences type I IFN production during ASFV infection, we designed and synthesized four siRNAs targeting A151R (**Table 1**). Our analysis indicated that siA151R#1 and siA151R#4 were the most effective siRNAs in down-regulating *A151R* expression (data not shown), and thus we used these two siRNAs to do the following experiments. PAMs were transfected with different doses of siA151R#1 and siA151R#4 for 24 h, and then infected with ASFV (MOI = 1). At 24 h later, cells were harvested to analyze the mRNA expression of *A151R*, *B646L* (ASFV p72) and *IFN-β* by qRT-PCR. The results showed that knockdown of pA151R significantly enhanced *IFN-β* expression, while the expression of *A151R* and *B646L* was down-regulated (**Figure 8F**). We also analyzed the viral titer in the supernatants from ASFV infected cells by using TCID_50_ assay. As shown in **Figure 8G**, knock-down of pA151R led to a notable inhibition of ASFV proliferation. Taken together, our findings indicate that ASFV pA151R might play a role in modulating type I IFN production during ASFV infection.

## 3 Discussion

As a dsDNA virus, ASFV is recognized by DNA sensor to trigger innate immune response. However, it is reported that the highly virulent ASFV dramatically inhibits the type I IFN signaling pathway to evade host immune system (24, 26, 40). In this study, ASFV pA151R was identified as a new powerful inhibitor, which decreased type I IFN production by disrupting cGAS-STING signaling pathway.

It is reported that cGAS-STING signaling pathway plays an indispensable role in host antiviral defense during DNA virus infection (41). However, viruses have evolved various strategies to interfere with different elements in cGAS-STING signaling pathway to available for their replication. For instance, porcine circovirus type 2 (PCV2) infection directly silences the catalytic activity of cGAS to inhibit type I IFN expression and facilitate virus replication (42). HSV-1 UL46 negatively regulates STING downstream anti-virus immunity response via binding to STING and disrupting its functions (43). In addition, many human cytomegalovirus (HCMV) proteins can inhibit type I IFN signaling pathway to evade host defense. Mechanistically, UL31 suppresses cGAS enzymatic functions and reduces cGAMP production through interacting with cGAS and disassociating DNA from cGAS (44). UL82 impairs the translocation of STING from the ER to perinuclear microsomes and inhibits the recruitment of TBK1 and IRF3 to the STING complex (45), whereas UL83 antagonizes the antiviral immune response via interacting with the IFI16 and blocking its oligomerization (46). Furthermore, recent studies have shown that several ASFV proteins inhibit type I IFN signaling pathway. ASFV DP96R inhibits TBK1 function to reduce type I IFN production (25). ASFV MGF-505-7R inhibits type I IFN signaling pathway via targeting STING and IRF3. Mechanistically, MGF-505-7R degrades STING by promoting the expression of the autophagy-related protein ULK1 and it also interacts with IRF3 leading to prevent IRF3 nuclear translocation (26, 47). ASFV pI215R and pA137R inhibit TBK1 activity via reducing TBK1 ubiquitination and degrading TBK1 expression respectively. In this study, we found that overexpressed ASFV pA151R significantly inhibited cGAS-STING-mediated IFN-β signaling pathway (**Figure 1**). Importantly, knockdown pA151R in ASFV could increase IFN-β production and inhibit ASFV replication (**Figure 8F**).

TBK1 is the crucial kinase in innate immune system, it stimulates transcription factor IRF3 and NF-κB in multiple signaling pathways during viruses infection to induce interferon and inflammatory factor expressions (37, 48, 49). It is reported that TBK1 polyubiquitination could enhance its kinase activity and TBK1 K63-linked polyubiquitination is a prerequisite for the activation of Ser172 phosphorylation (21). By using luciferase assay, we found overexpressed pA151R inhibited cGAS, STING and TBK1 but not IKKα and IRF3-5D induced IFN-β promoter activities (**Figure 2**). Further study showed that pA151R significantly decreased TBK1 ubiquitination and phosphorylation induced by cGAS-STING (**Figure 3**). These results suggest ASFV pA151R restrains TBK1 activity to inhibit host antivirus response. Nevertheless, pA151R could not interact with TBK1. In contrast, it interacted with E3 ligase TRAF6 (**Figures 4A–C**). Previous studies have demonstrated that E3 ligases TRAFs family function downstream of MAVS to activate TBK1 K63-linked-polyubiquitination and TRAF6 is the most essential element (20, 50). However, the relationship between TRAF6 and TBK1 in STING complex is unclear. In this study, we explored the influence of TRAF6 on TBK1 by using 2’3-cGAMP activated STING. The data indicated that in cGAS-STING signaling pathway, TRAF6 could facilitate TBK1 activation (**Figures 4D-F**; **5E**). Our findings testify TRAF6 as E3 ligase could assist TBK1 ubiquitination in STING-TRAF6-TBK1 complex, which is supplementing the mechanisms of cGAS-STING signaling pathway and the functions of TRAF6 as an important antiviral factor.

Recently, there are increasing reports demonstrating that ASFV accomplishes immune escape not only by inhibiting type I IFN but also by impeding immune-related inflammatory cytokines production to facilitate its proliferation (51–54). ASFV pI329R inhibits toll like receptor 3 (TLR3)-meditated IFN-β and proinflammatory cytokines production through targeting TRIF, an adaptor protein in TLRs pathway (55). ASFV MGF-505-7R interacts with NLRP3 to inhibit NLRP3 inflammasome assembly to decrease mature IL-1β secretion (47). The pE199L promotes the activation pro-apoptotic factor Bax and its translocation to mitochondria, leading to mitochondrial dependent apoptosis (56). The pS273R inhibit ASFV infection-induced pyroptosis to regulate the inflammatory response through interacting with GSDMD (57). These studies indicate that ASFV genes could negatively regulate host immune responses in multiple ways. It has been reported that TRAF6 as an adapter protein that participates a wide array of the regulation of immune responses to maintain homeostasis in cells. Functionally, TRAF6 could activate mitogen-activated protein kinase (MAPK), phosphoinositide 3-kinase (PI3K) signaling pathway to trigger proinflammatory transcription factor NF-κB and AP-1 activation (58–60). In this study, we proved that pA151R decreased TRAF6 expression leading to inhibit the modification of TRAF6 on TBK1 and suppress the interaction between TRAF6 and TBK1 (**Figure 5**). By using different degradation pathway inhibitors screen, we demonstrated pA151R degraded TRAF6 through apoptosis pathway to impede its function (**Figure 6**). Based on our findings and the functions of TRAF6 in other immune signaling pathway, we speculate that pA151R might be a negative regulation factor for inflammatory response. In addition, pA151R might activated other signal molecules to execute the degradation of TRAF6. However, these hypotheses should be further experimented to exploration.

In a recent paper, the crystal structure of pA151R was determined. It is reported that pA151R comprises of a thioredoxin active site feature motif (W131, C132, T133, K134, C135) and a Zn-binding motif (H102, C109, C132, C135) (39). According to these two potentially activated motifs, several mutants were constructed, and an unbiased method was used to screen the pA151R mutants involved in inhibiting type I IFN production induced by cGAS-STING. Our data indicated the mutants of pA151R (H102A, C109A, C132A, C135A) lost the ability of inhibit type I IFN production (**Figures 7A–E**). These mutants are unable to coordinate a Zn^2+^ ion to form a Zn-binding motif. Interestingly, they remained the ability to interact with TRAF6 but fail to degrade TRAF6 (**Figures 7F, G**). Consistently, the mutants of pA151R no longer promoted HSV-1 replication via inhibiting type I IFN signaling pathway (**Figures 8A-E**). These results suggest that the degradation of TRAF6 by pA151R is irrelevant to the interaction of TRAF6 and pA151R. ASFV pA151R function is complicated and need further exploration. In agreement with our results, previous reports have indicated that pA151R is identified to be a thioredoxin and it is essential for the virus morphogenesis. Besides, inhibition of A151R translation by siRNA significantly decreases virus production in Vero cells (31, 32). Of note, a recent study reports that deletion of pA151R (ASFV-G-ΔA151R) significantly decreased ASFV replication and virus virulence in pigs (33). In this study, we used siRNA interfering A151R expression during ASFV infection in PAMs and the result indicated that knockdown of pA151R significantly inhibited ASFV *B646L* (p72) expression and increased IFN-β production (**Figure 8F**). Our study provides a complementary support that ASFV-G-ΔA151R is attenuated.

In summary, our data proved that ASFV pA151R inhibited cGAS-STING signaling pathway to downregulate type I interferon production by targeting TBK1. Mechanically, we demonstrated pA151R impeded TBK1 K63-linked polyubiquitination through degrading TRAF6 in a caspase-dependent manner. Importantly, we verified that the deficiency of pA151R up-regulated type I IFN production in PAM cells infected with ASFV to suppressed ASFV growth. These findings identify pA151R as a powerful interferon repressor and help us further understand how ASFV evades the host innate immune system.

## 4 Materials and methods

### 4.1 Cells and viruses

HEK-293T cells (ATCC CRL-3216) and HeLa (ATCC CCL-2) cells were cultured in Dulbecco’s modified Eagle’s medium (DMEM) (Gibco) with 10% fetal bovine sera (FBS) and 1% penicillin-streptomycin. CRL-2843 cells (ATCC CRL-2843), a porcine alveolar macrophage cell line, were maintained in RPMI 1640 (Gibco) medium containing 10% FBS. Porcine alveolar macrophages (PAMs) were obtained from lung lavage of 6–8-week-old specific pathogen-free (SPF) piglets (the Large White breed) and were cultured in RPMI 1640 (Gibco) medium containing 10% FBS. All the cells were grown in a humidified atmosphere containing 5% CO_2_ at 37°C.

The ASFV HLJ/18 strain was isolated from a farm in northeastern China during the ASF outbreak in 2018 (3). Herpes Simplex Virus-1 (HSV-1) were provided by Professor Wang Xiaojia, College of Veterinary Medicine, China Agricultural University. The viral titers were determined using the Reed-Muench method and expressed as tissue culture infective dose 50% (TCID_50_).

### 4.2 Antibodies and reagents

Anti-TBK1/NAK (3504), anti-phospho-TBK1/NAK (5483), anti-IRF3 (10949), anti-phospho-IRF3 (4947), anti-NF-κB-p65 (8242), anti-phospho-NF-κB-p65 (3033), anti-TRAF6 (8028), anti-ub (20326), anti-ub-k63 (5621), anti-Myc-Tag (2276) and anti-Flag-Tag (14793) were purchased from Cell Signaling Technology (USA). Anti-phospho-IRF-3(Ser396) (SAB5700435), anti-HA-tag (H6908), anti-β-actin (A5441), and anti-Flag M2 Affinity Gel (A2220) were purchased from Sigma (USA). Anti-ub-k48 (EP8589) was purchased from Abcam (UK). Protein G Sepharose 4 Fast Flow was from GE Healthcare (Sweden). HRP-conjugated goat anti-mouse or anti-rabbit secondary antibodies for Western blot were purchased from Santa Cruz (USA). JetPRIME kit was obtained from Polyplus Transfection (France). Anti-pA151R polyclonal antibody was produced in rabbit by immunization with recombinant pA151R. Double-Luciferase Reporter Assay Kit was from Promaga (USA). 2’3’-cGAMP, poly(dA:dT), and Lipofectamine 3000 were purchased from Invitrogen (USA). 3-Methyladenine (3-MA, HY-19312), MG-132 (HY-13259), E-64 (HY-15282), and Z-VAD-FMK (HY-16658B) were from MedChemExpress (MCE) (USA).

### 4.3 Plasmids construction

Porcine IFN-β- luciferase reporter plasmid was constructed using pGL3-Basic vector as described elsewhere (61). Transcription factor IRF3, NF-κB and ISRE responding elements were synthesized, and then separately cloned into pGL3-basic vector (62). The pRL-TK containing Renilla luciferase was used as a normalization control. The plasmids pRK5-Flag-cGAS, pRK5-Flag-STING, pRK5-Flag-TBK1, pRK5-Flag-IRF3, pRK5-Flag-IRF3-5D, and pRK5-Flag-IKKα were constructed by own laboratory. The porcine TRAF3 and TRAF6 gene were amplified by standard RT-PCR using total RNA extracted from PAMs as templates, and the cDNAs were cloned into the pRK5-(Flag-tag) or (HA-tag) vector. All genes used here are porcine genes. ASFV *A151R* gene was synthesized by the Beijing Genomics Institute (BGI). The expression plasmids of pA151R were cloned into the pRK5-(Flag-tag), (HA-tag), or pCMV-Myc. Q5 Site-Directed Mutagenesis Kit was employed for HA-A151R mutant constructions. All the primers were listed in **Table 2**.

**Table 2:** Primers used for PCR.

Primer	Sequence (5’→3’)
pRK5-Flag-TRAF3-F	GACGATGACAAGGGATCCATGACACACAGAATGGAG
pRK5-Flag-TRAF3-R	AGTTGGGCCATGGCGGCCATCAGGGGTCAGGCAGATC
pRK5-Flag-TRAF6-F	GACGATGACAAGGGATCCATGAGTCTGCTACATTGTGAA
pRK5-Flag-TRAF6-R	AGTTGGGCCATGGCGGCCACTATGTCCCCGAGTCTGTACTT
pRK5-HA-TRAF6-F	GACTATGCGGGCGGATCCATGAGTCTGCTACATTGTGAA
pRK5-HA-TRAF6-R	AGTTGGGCCATGGCGGCCACTATGTCCCCGAGTCTGTACTT
pRK5-Flag-A151R-R	TGCACTGCAGTTATTGGAATATATTGGGCG
pRK5-HA-A151R-F	CGCGGATCCATGATGGCGTTGTTACACAAA
pRK5-HA-A151R-R	TGCACTGCAGTTATTGGAATATATTGGGCG
pCMV-Myc-A151R-F	ATGGAGGCCCGAATTCGGATGATGGCGTTGTTACACA
pCMV-Myc-A151R-R	CCGCGGCCGCGGTACCTTTATTGGAATATATTGGG
HA-A151R-5A-F	TGCAGCCACATCCTTTTACCCATTTAC
HA-A151R-5A-R	GCAGCCGCAATGCTACGATCATTAAAGATATG
HA-A151R-W131A-F	TCGTAGCATTGCGTGTACTAAATGC
HA-A151R-W131A-R	TCATTAAAGATATGAGGACCG
HA-A151R-C132A-F	TCGTAGCATTTGGGCTACTAAATGCACA
HA-A151R-C132A-R	TCATTAAAGATATGAGGACCG
HA-A151R-T133A-F	TCGTAGCATTTGGTGTGCTAAATG
HA-A151R-T133A-R	TCATTAAAGATATGAGGACCG
HA-A151R-K134A-F	TTGGTGTACTGCATGCACATCCTTTTAC
HA-A151R-K134A-R	ATGCTACGATCATTAAAGATATG
HA-A151R-C135A-F	TTGGTGTACTAAAGCCACATCCTTTTACCC
HA-A151R-C135A-R	ATGCTACGATCATTAAAGATATG
HA-A151R-H102A-F	TGATTTATATgctACAAATTACAATCATAAATGTATAAAAG
HA-A151R-H102A-R	TTTTCAAAATAAATTTGAGTACG
HA-A151R-C109A-F	CAATCATAAAgctATAAAAGATTTTTGGAATGTTTCAAC
HA-A151R-C109A-R	TAATTTGTATGATATAAATCATTTTCAAAATAAATTTG

### 4.4 Luciferase reporter assays

Cells were transiently transfected with pRL-IFN-β–Luc, pRL-ISRE–Luc, pRL-IRF3–Luc or pRL-NF-κB–Luc, pRL-TK, and the indicated plasmids for 24 h. Cell extracts were prepared and analyzed for firefly and Renilla luciferase activities using a Dual-Luciferase reporter assay kit (Promega) according to the manufacturer’s instructions.

### 4.5 RNA extraction and real-time PCR

Cells cultured in 24-well plates were transiently transfected with the indicated plasmids for 24 h and treated with different agonists or infected with viruses. Total RNA was extracted from treated cells with TRIzon reagent (CWBIO) according to the manufacturer’s instructions. Quantitative real-time PCR analysis was performed in a ViiA7 real-time PCR System with SYBR Green real-time PCR Master Mix (Mei5 Biotechnology). The 2^-ΔΔCT^ Ct method was used to calculate the relative expression of the target gene. Gene-specific primers for *IFN-β*, *ISG15*, *ISG54*, *ISG56*, *MX1*, *CXCL10*, *TRAF6*, *A151R* and *GAPDH* were designed and listed in **Table 3**. The indicated genes expressions were normalized to glyceraldehyde-3-phosphate dehydrogenase (GAPDH) (5’CCTTCCGTGTCCCTACTGCCAAC3’[forward] and 5’ GACGCCTGCTTCACCCTTCT3’ [reverse]) and presented as the change (n-fold) in induction relative to the control.

**Table 3:** Primers used for Quantitative real time PCR.

Primer	Sequence (5’→3’)
Human GAPDH-F	CTGTTCGACAGTCAGCCGCATC
Human GAPDH-R	GCGCCCAATACGACCAAATCCG
Human IFNβ-F	ACGCCGCATTGACCATCTAT
Human IFNβ-R	GTCTCATTCCAGCCAGTGCT
Human TRAF6-F	GGCCCAGGCTGTTCATAGTT
Human TRAF6-R	CAGCTCCCGGATTTGATGGT
Porcine GAPDH-F	CCTTCCGTGTCCCTACTGCCAAC
Porcine GAPDH-R	GACGCCTGCTTCACCACCTTCT
Porcine IFNβ-F	AGCACTGGCTGGAATGAAAC
Porcine IFNβ-R Porcine IFNα-F Porcine IFNα-R	TCCAGGATTGTCTCCAGGTC CTGCTGCCTGGAATGAGAGCC TGACACAGGCTTCCAGGTCCC
Porcine ISG15-F Λ	TGAAGATGCTGGGAGGCAAG
Porcine ISG15-R	CACCCCATCCTGAAGCACAT
Porcine ISG56-F	TTCCGACACGCAGTCAAGTT
Porcine ISG56-R	GTAGCAAAGCCCTGTCTGGT
Porcine ISG54-F	TCTGTGGCTTTGCACCTCTT
Porcine ISG54-R	GGGGTTTCAGCTCCATTCCA
Porcine MX1-F	CACAGAACTGCCAAGTCCAA
Porcine MX1-R	GCAGTACACGATCTGCTCCA
Porcine CXCL10-F	TGCCCACATGTTGAGATCAT
Porcine CXCL10-R	CGGCCCATCCTTATCAGTAG
Porcine TRAF6-F	TCGCAGTAGCTCCTGTACCT
Porcine TRAF6-R	CTGAGCAACAGCCAGAGGAA
HSV-1 UL30-F	CCGGCCATCAAGAAGTACGA
HSV-1 UL30-R	CTGGGCTAGCTTCACCACCTTCT
ASFV B646L-F	TTGGCCCAAGACTTGCTGAATAGC
ASFV B646L-R	ATACGTTGCGTCCGTGATAGGAGT
ASFV A151R-F	TGATGGCGTTGTTACACAAAGAA
ASFV A151R-R	AGAAGCAATATTGTCCCGCCA

### 4.6 ELISA

The IFN-β protein levels in cell culture supernatants were measured using porcine IFN-β ELISA kits (Gene-Protein Link) in accordance with the manufacturer’s instructions.

### 4.7 RNA interference

The transfection of siRNA was performed with HiPerFect Transfection Reagent (QIAGEN, Germantown, MD) following the manufacturer’s instructions. Cells were transfected with siRNA for 24 h and then treated with poly(dA:dT) or 2’3’-cGAMP for another 12 h. The siRNA knockdown efficiency of the target gene was assessed by quantitative real-time PCR (qRT-PCR). The target sequences of small interfering RNAs (siRNAs) are listed in **Table 1**.

### 4.8 Western blot

Cells were harvested and lysed with ice-cold RIPA lysis buffer (CWBIO) on ice for 15 min. Protein levels were quantified by using bicinchoninic acid (BCA) assay. The same amount of protein from each extract was separated by SDS-PAGE and transferred to polyvinylidene difluoride membranes (Millipore). Membranes were blocked with 5% skim milk in phosphate-buffered saline (PBS) with 0.05% Tween 20 (PBST) for 1 h, and then incubated for 1-2 h at room temperature or overnight at 4°C with antibodies at a suitable dilution as recommended (anti-p-TBK1, -TBK1, -p-IRF3, -IRF3, -p-NF-κB-p65, -NF-κB-p65, -TRAF6, -Flag, -HA, -Myc at 1:1000; anti-β-actin, -GAPDH at 1:3000). The membranes were then incubated with the appropriate secondary antibody for 1 h at a dilution of 1:5000. After washing, signals were visualized using enhanced chemiluminescence (Millipore) according to the manufacturer’s protocols.

### 4.9 Co-immunoprecipitation and ubiquitination analysis

Cells were collected and lysed with a lysis buffer (50 mM Tris (pH 7.4), 150 mM NaCl, 1% NP-40, 10% glycerol and 1 mM EDTA (pH 8.0)) supplemented with 1 mM PMSF and 1 mM NaF for 20 min on ice. The lysates were centrifuged at 13,000 rpm for 15 min at 4°C to remove the debris. Cell lysates were incubated with anti-Flag M2 Affinity Gel or Protein G Sepharose 4 Fast Flow plus pre-specified antibodies at 4°C more than 6 h. For immunoblot analysis, equal amounts of cell lysates and immunoprecipitants were separated by 6-12% SDS-PAGE and then transferred to a polyvinylidene difluoride membrane (Millipore). Membranes were blocked with 5% skim milk in PBS with 0.05% Tween-20, followed by incubation 2 h at room temperature or overnight at 4°C with the primary antibodies. After incubation with secondary antibodies, the membranes were visualized using enhanced chemiluminescence (Millipore).

### 4.10 Statistical analysis

Results are presented as means ± SD for at least three independent experiments. Data were analyzed by GraphPad Prism software using *Student’s t test*. Differences in data were statistically significant if the *P* value is less than 0.05. * *P* < 0.05; * * *P* < 0.01; * * * *P* < 0.001.

## Data availability statement

The original contributions presented in the study are included in the article/**Supplementary Materials**, further inquiries can be directed to the corresponding author/s.

## Author contributions

YL: Conceptualization, Data curation, Investigation, Methodology, Software, Writing – original draft. LH: Data curation, Methodology, Writing – original draft. HL: Methodology, Software, Writing – original draft. YZ: Methodology, Software, Writing – original draft. ZY: Methodology, Software, Writing – original draft. XZ: Methodology, Software, Writing – original draft. CW: Supervision, Validation, Writing – review & editing. W-HF: Conceptualization, Formal Analysis, Funding acquisition, Project administration, Resources, Supervision, Validation, Visualization, Writing – review & editing.

## Supplementary material

Supplementary Figure 1 ASFV pA151R negatively regulates type I IFN production induced by cGAS-STING. **(A)** HEK-293T cells cultured in 24-well plates were transfected with IL-6 luciferase (Luc) reporter (40 ng) and pRL-TK (4 ng), Flag-cGAS (20 ng) and Flag-STING (80 ng), and different doses of HA-A151R (0, 100, 200, or 400 ng) for 24 h followed by Dual-Luciferase assay. (B) CRL-2843 cells cultured in 24-well plates were transfected with different doses of HA-A151R (0, 100, 200, or 400 ng). At 24 hpt, cells were treated with poly(dA:dT) (1 μg/ml) for 12 h, and the mRNA levels of *TBK1* was then analyzed by qRT-PCR assay. The data are representative of three independent experiments (means ± the standard errors of the mean [SEM]). ns, not significant (Student t test).

Supplementary Figure 2 ASFV pA151R inhibits TBK1 K63-linked polyubiquitination and phosphorylation. **(A)** Flag-STING and HA-ubiquitin (Ub) were co-transfected with or without Myc-A151R into HEK-293T cells cultured in 6-well plates for 24 h. The cell lysates were immunoprecipitated with anti-Flag (M2) beads for the detection of STING ubiquitination by using the indicated antibodies.
