# Supplementary figures and images for: ASFV pA151R negatively regulates type I IFN production via degrading E3 ligase TRAF6 (part 1 of 2)
Source: Front Immunol. 2024 Feb 21;15:1339510. doi: 10.3389/fimmu.2024.1339510 (PMC10914938; doi:10.3389/fimmu.2024.1339510)

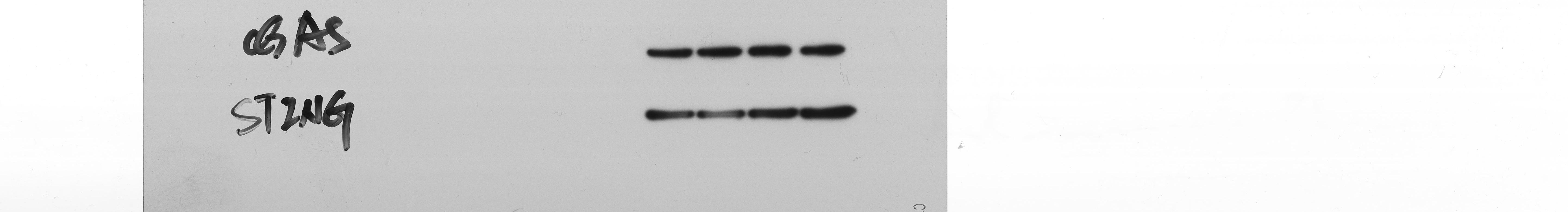

Supplement: Supplementary file 2 [file DataSheet_2.zip › fig 2/2C/fig2C Flag-cGAS.jpg]

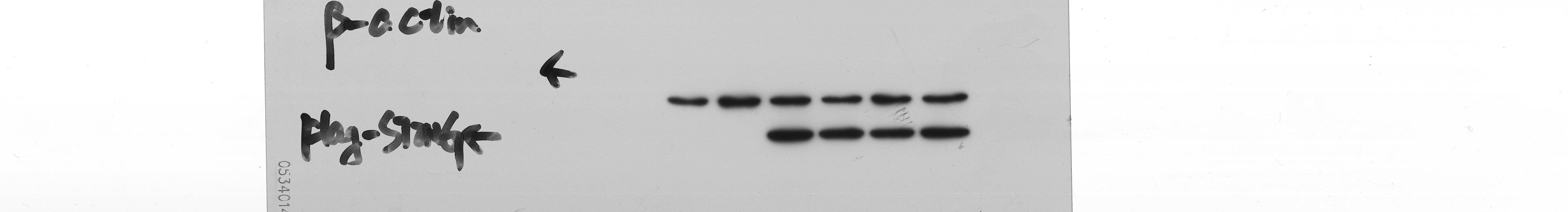

Supplement: Supplementary file 2 [file DataSheet_2.zip › fig 2/2C/fig2C Flag-STING and actin.jpg]

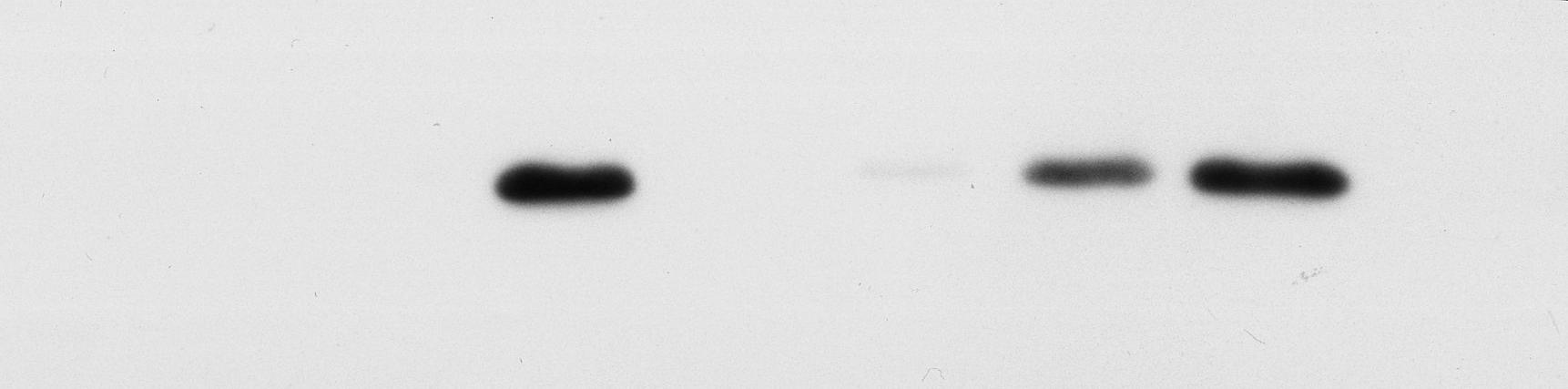

Supplement: Supplementary file 2 [file DataSheet_2.zip › fig 2/2C/fig2C HA-A151R.jpg]

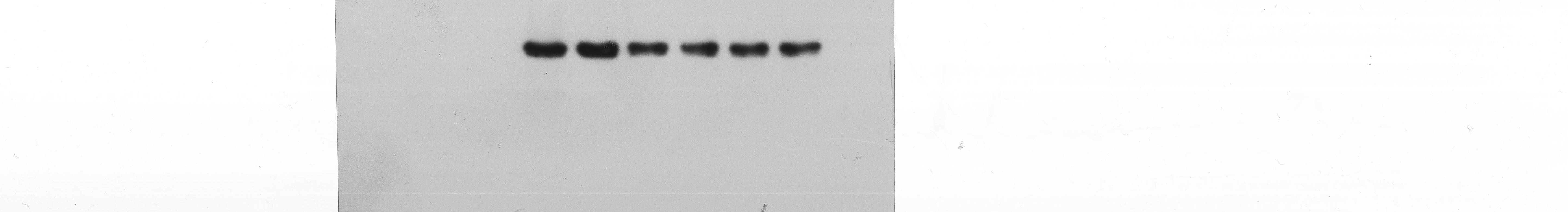

Supplement: Supplementary file 2 [file DataSheet_2.zip › fig 2/2C/fig2C IRF3.jpg]

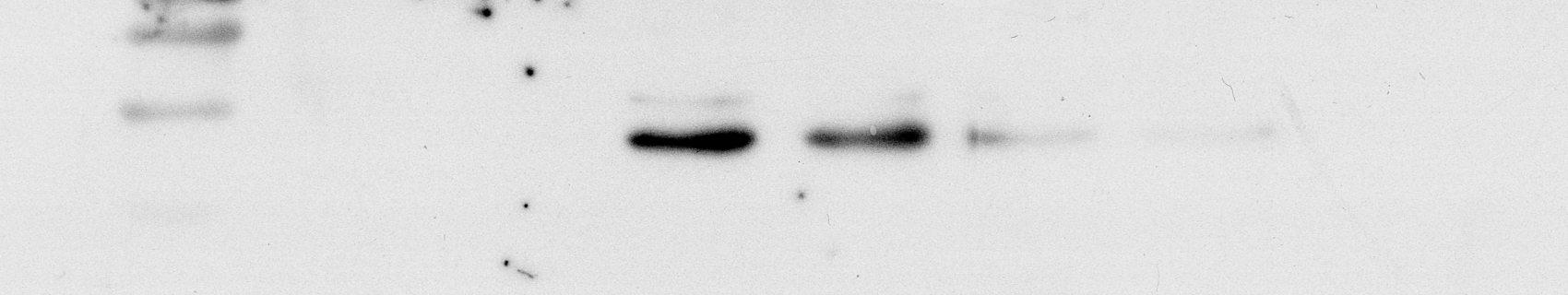

Supplement: Supplementary file 2 [file DataSheet_2.zip › fig 2/2C/fig2C p-IRF3.jpg]

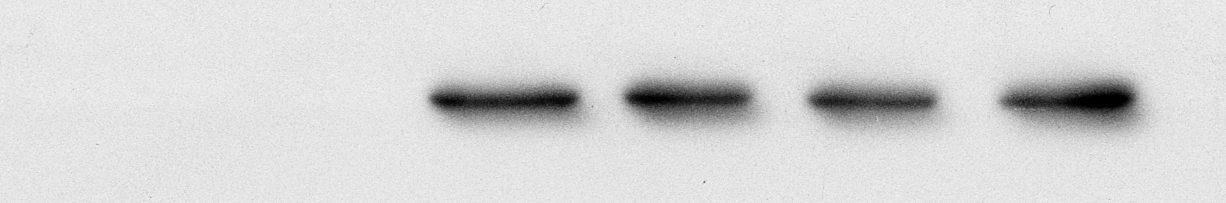

Supplement: Supplementary file 2 [file DataSheet_2.zip › fig 2/2D/fig2D Flag-cGAS.jpg]

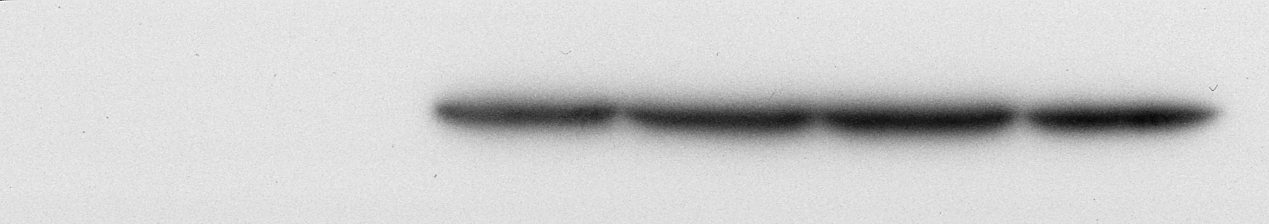

Supplement: Supplementary file 2 [file DataSheet_2.zip › fig 2/2D/fig2D Flag-STING.jpg]

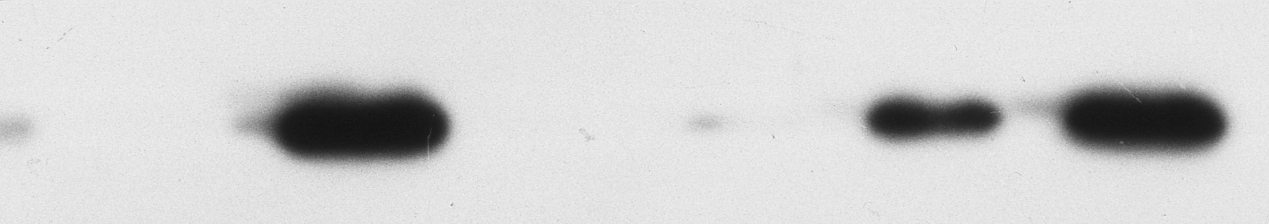

Supplement: Supplementary file 2 [file DataSheet_2.zip › fig 2/2D/fig2D HA-A151R.jpg]

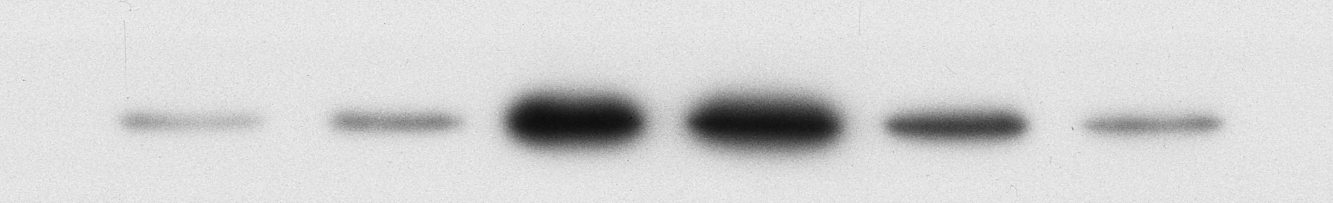

Supplement: Supplementary file 2 [file DataSheet_2.zip › fig 2/2D/fig2D p-p65.jpg]

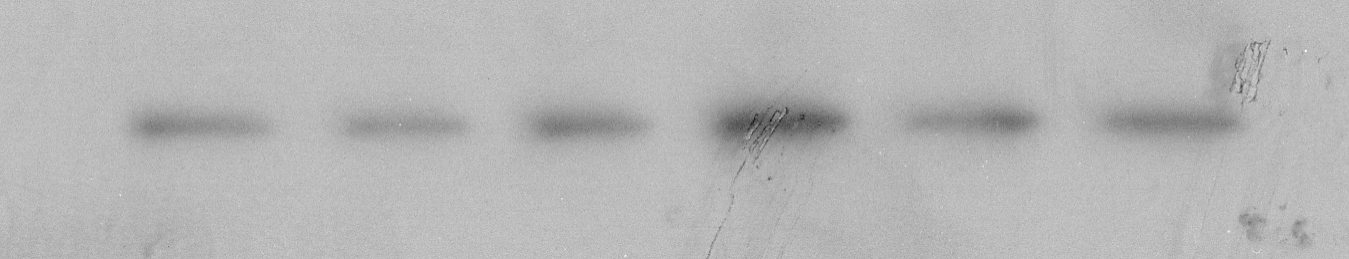

Supplement: Supplementary file 2 [file DataSheet_2.zip › fig 2/2D/fig2D p65.jpg]

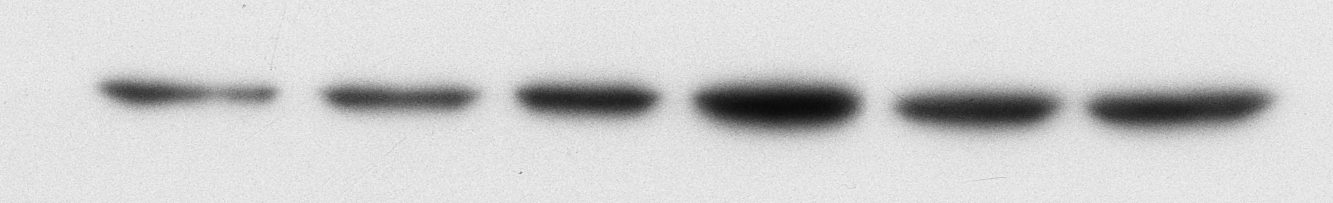

Supplement: Supplementary file 2 [file DataSheet_2.zip › fig 2/2D/fig2D a┬-actin.jpg]

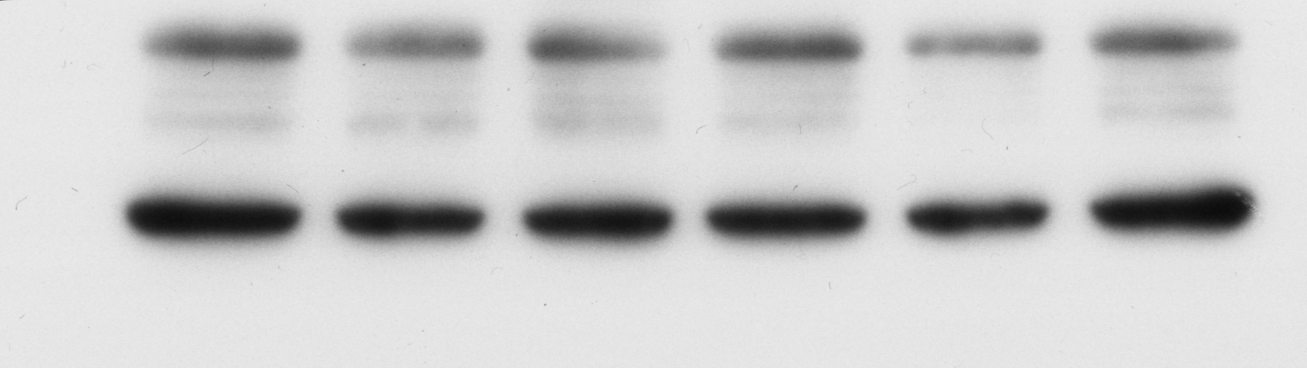

Supplement: Supplementary file 2 [file DataSheet_2.zip › fig 2/2F/fig2F B-actin.jpg]

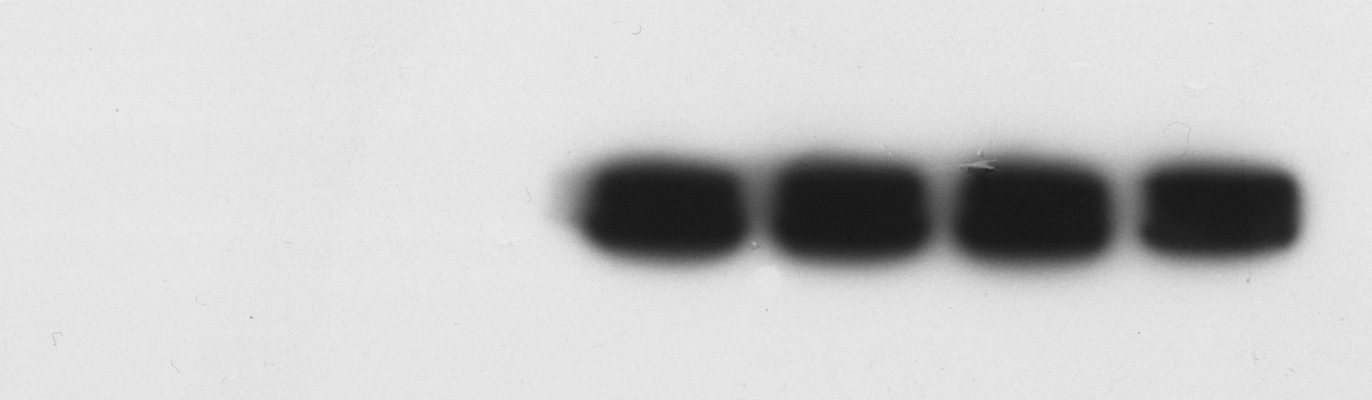

Supplement: Supplementary file 2 [file DataSheet_2.zip › fig 2/2F/fig2F Flag-TBK1.jpg]

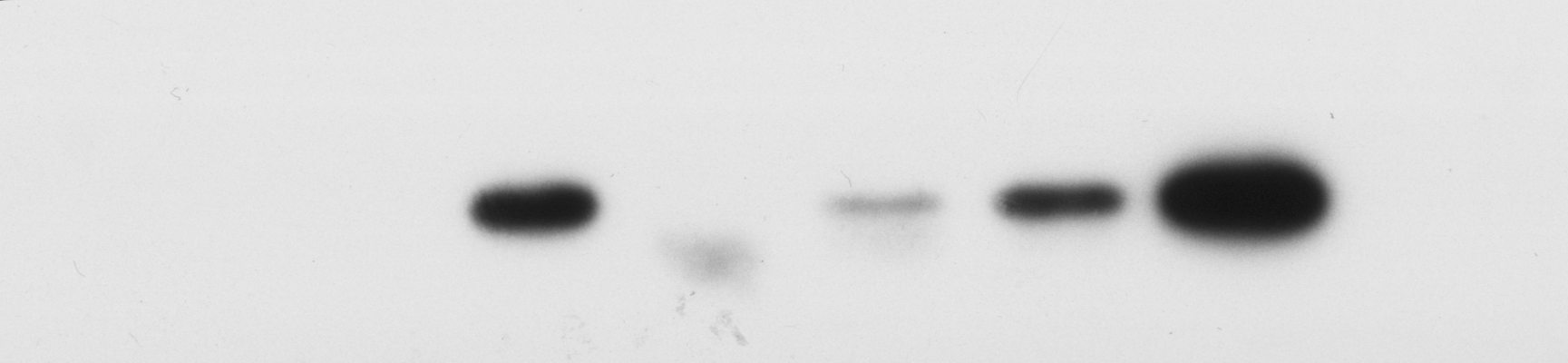

Supplement: Supplementary file 2 [file DataSheet_2.zip › fig 2/2F/fig2F HA-A151R.jpg]

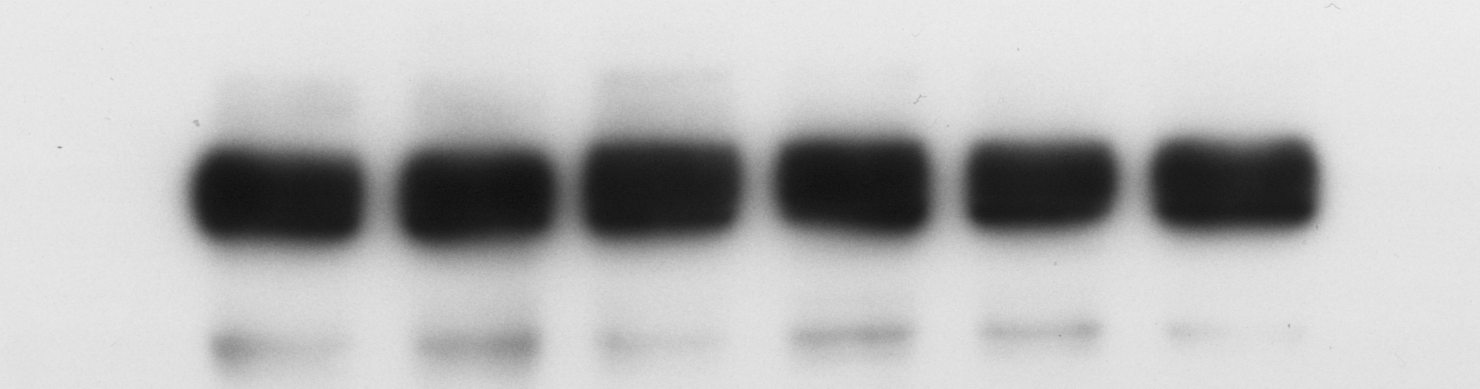

Supplement: Supplementary file 2 [file DataSheet_2.zip › fig 2/2F/fig2F IRF3.jpg]

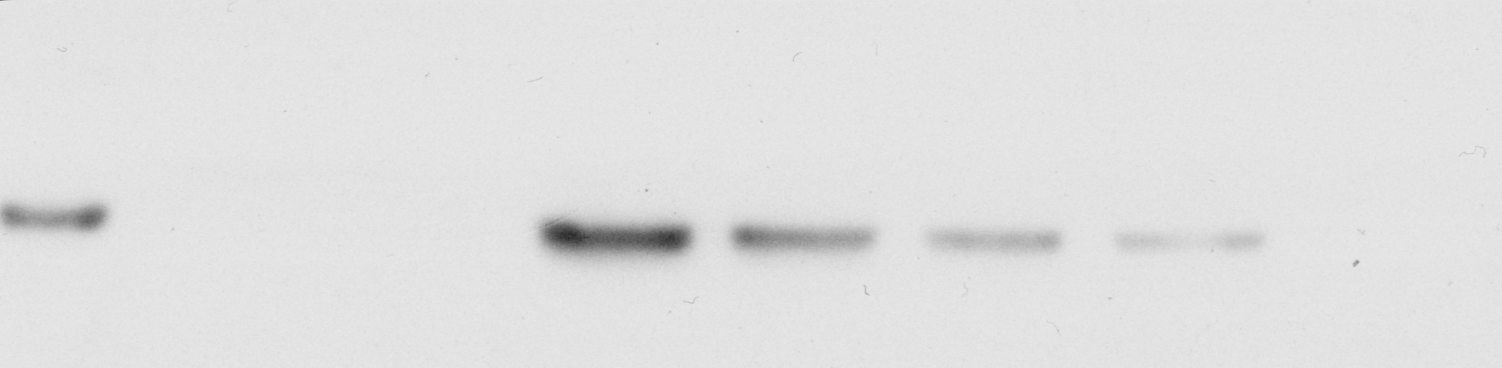

Supplement: Supplementary file 2 [file DataSheet_2.zip › fig 2/2F/fig2F p-IRF3.jpg]

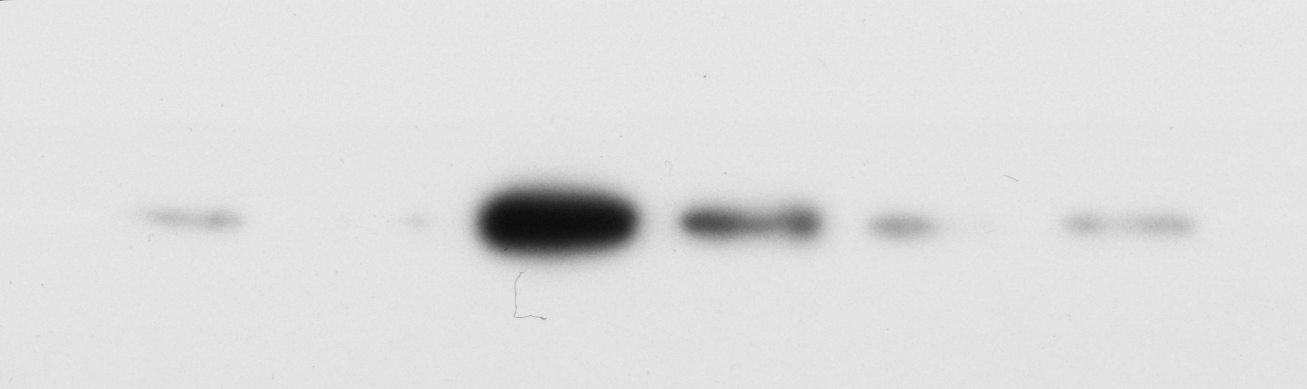

Supplement: Supplementary file 2 [file DataSheet_2.zip › fig 2/2F/fig2F p-P65.jpg]

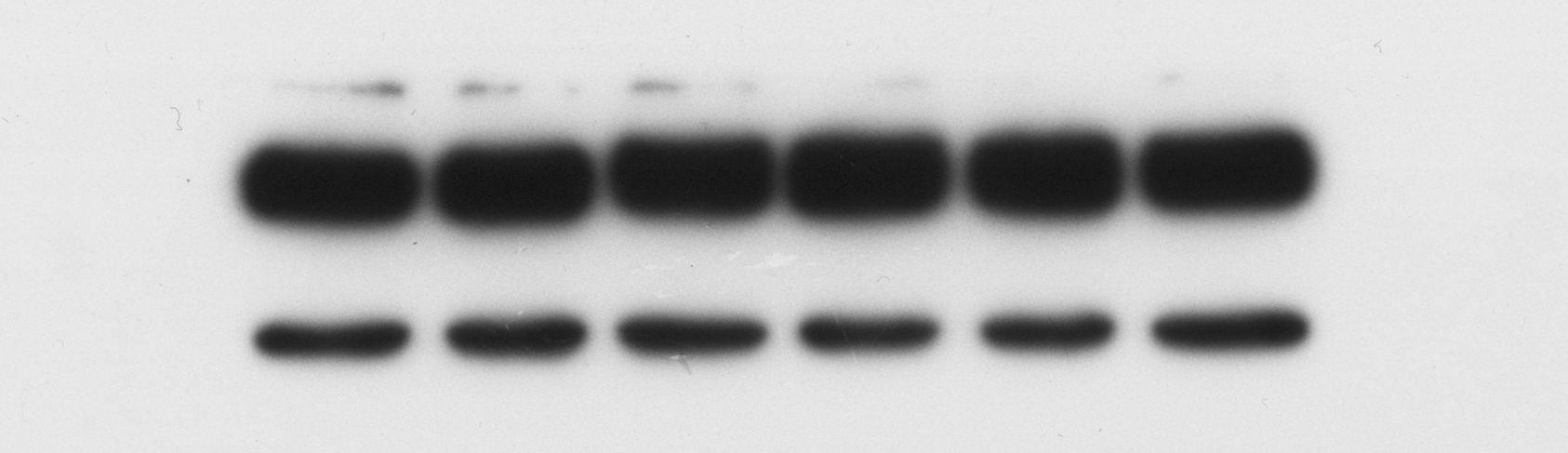

Supplement: Supplementary file 2 [file DataSheet_2.zip › fig 2/2F/fig2F P65 and B- ACTIN.jpg]

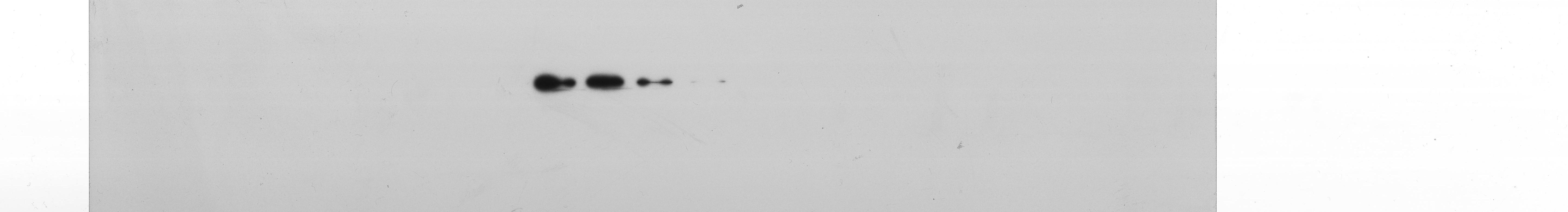

Supplement: Supplementary file 3 [file DataSheet_3.zip › fig 3/3A/fig3A p-TBK1.jpg]

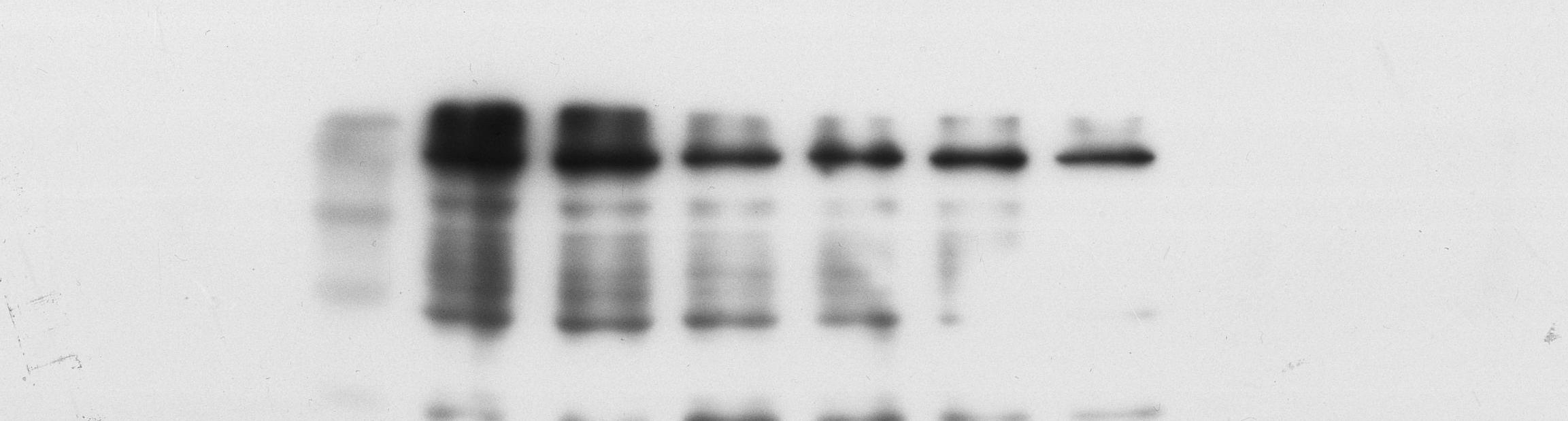

Supplement: Supplementary file 3 [file DataSheet_3.zip › fig 3/3A/fig3A TBK1.jpg]

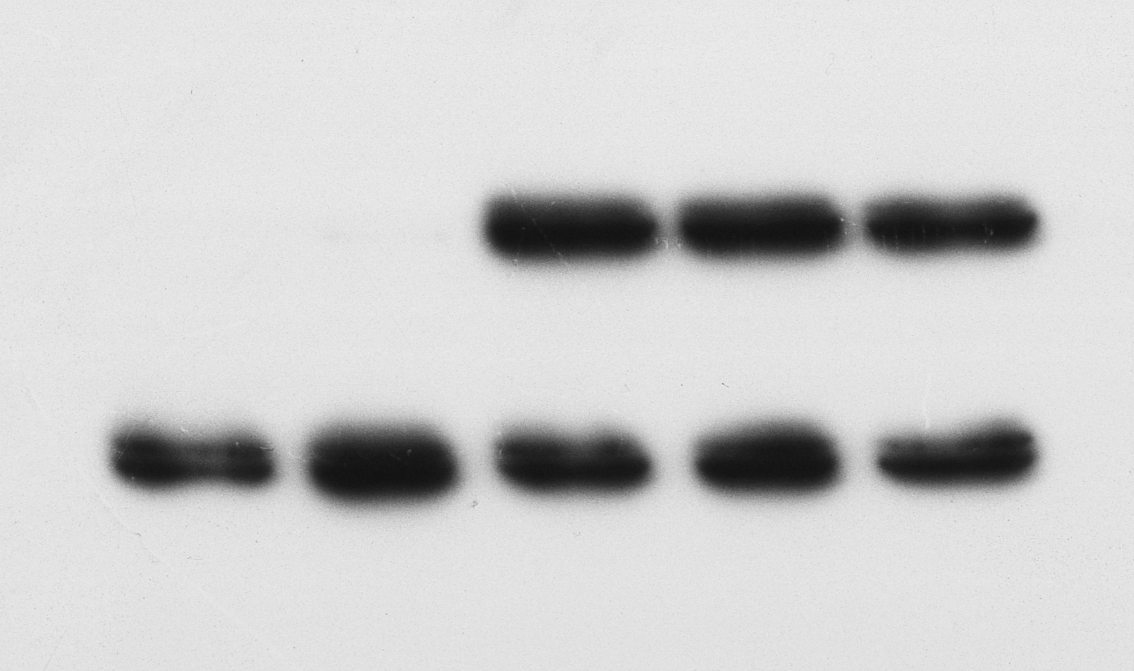

Supplement: Supplementary file 3 [file DataSheet_3.zip › fig 3/3B/fig3B Input-B-actin.jpg]

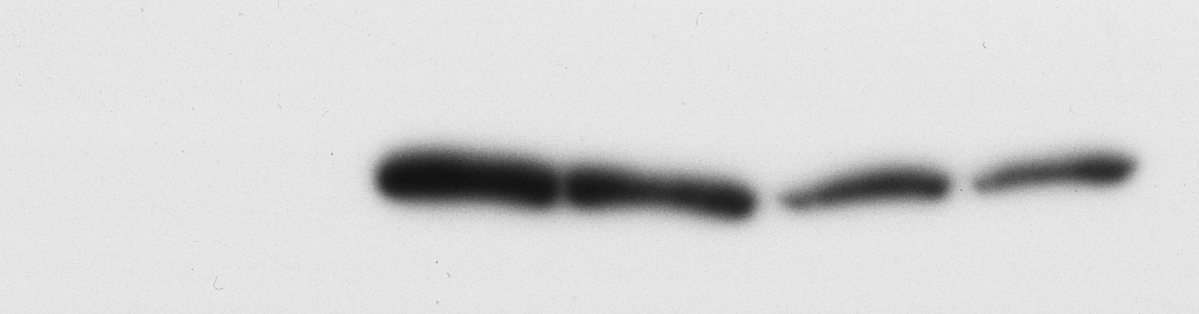

Supplement: Supplementary file 3 [file DataSheet_3.zip › fig 3/3B/fig3B Input-Flag-TBK1.jpg]

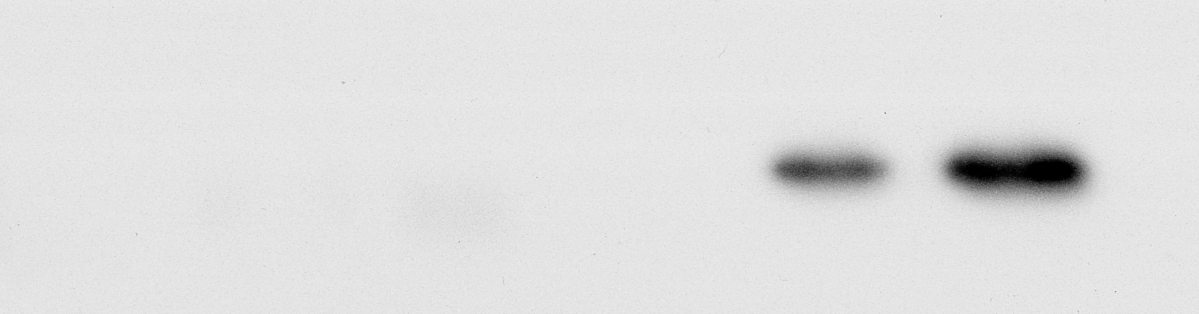

Supplement: Supplementary file 3 [file DataSheet_3.zip › fig 3/3B/fig3B Input-HA-A151R.jpg]

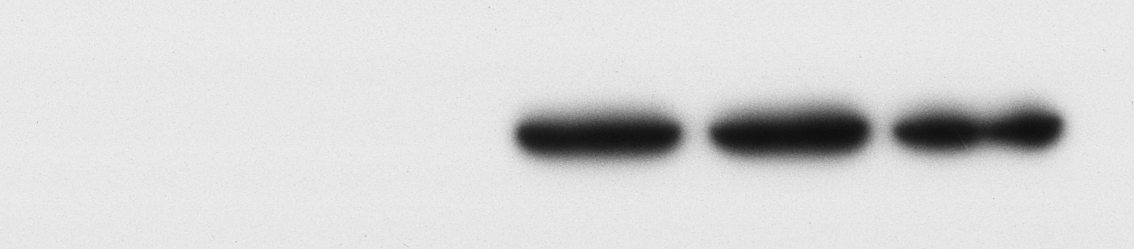

Supplement: Supplementary file 3 [file DataSheet_3.zip › fig 3/3B/fig3B Input-HA-TBK1.jpg]

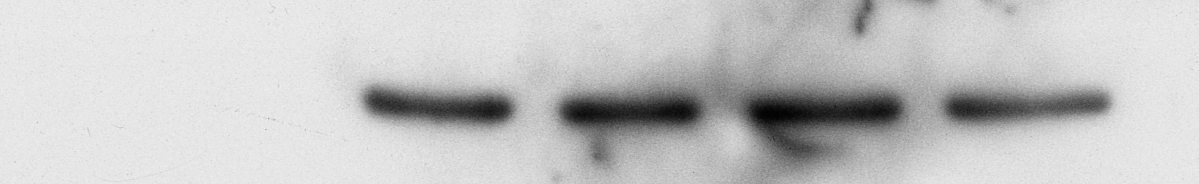

Supplement: Supplementary file 3 [file DataSheet_3.zip › fig 3/3B/fig3B IP-Flag-TBK1.jpg]

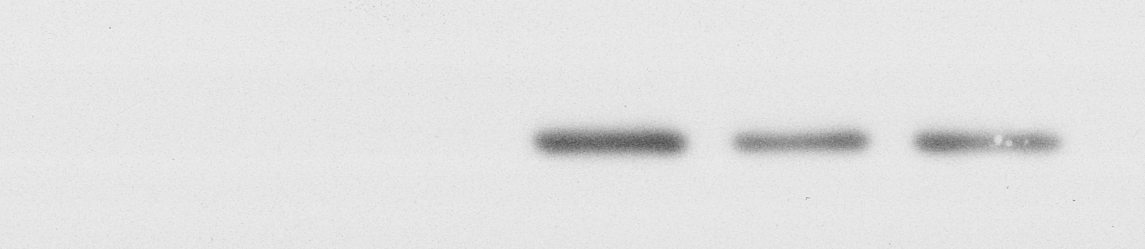

Supplement: Supplementary file 3 [file DataSheet_3.zip › fig 3/3B/fig3B IP-HA-TBK1.jpg]

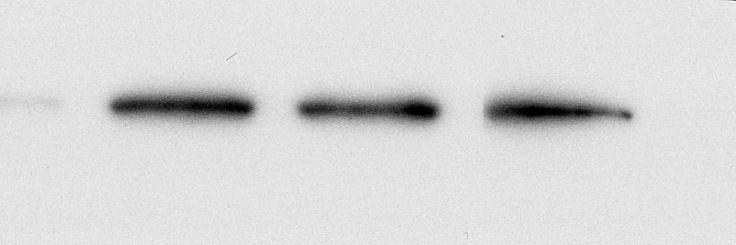

Supplement: Supplementary file 3 [file DataSheet_3.zip › fig 3/3C/fig3C Input Flag-TBK1 k48 for third figure.jpg]

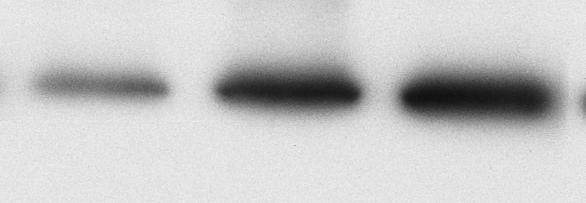

Supplement: Supplementary file 3 [file DataSheet_3.zip › fig 3/3C/fig3C Input Flag-TBK1 k63 for second figure.jpg]

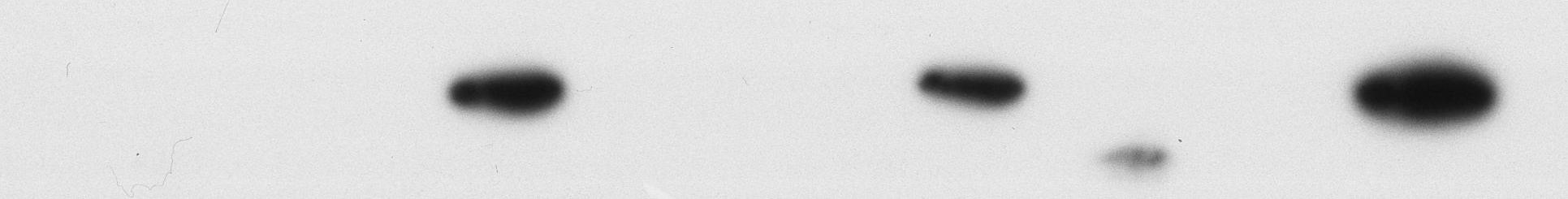

Supplement: Supplementary file 3 [file DataSheet_3.zip › fig 3/3C/fig3C Input HA-A151R for 3 figure.jpg]

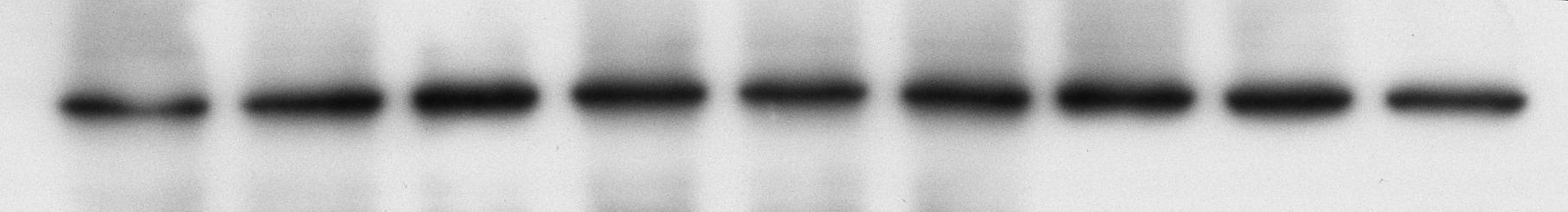

Supplement: Supplementary file 3 [file DataSheet_3.zip › fig 3/3C/fig3C Input a┬-actin for 3 figure.jpg]

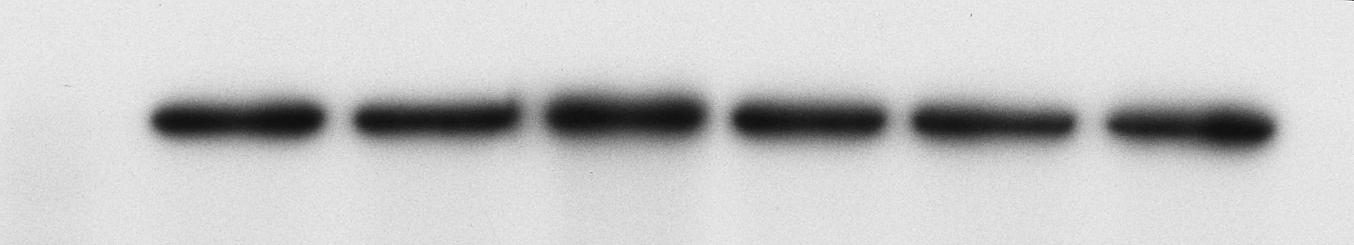

Supplement: Supplementary file 3 [file DataSheet_3.zip › fig 3/3C/fig3C Input-Flag-TBK1 Ub left 3 lane for first figure.jpg]

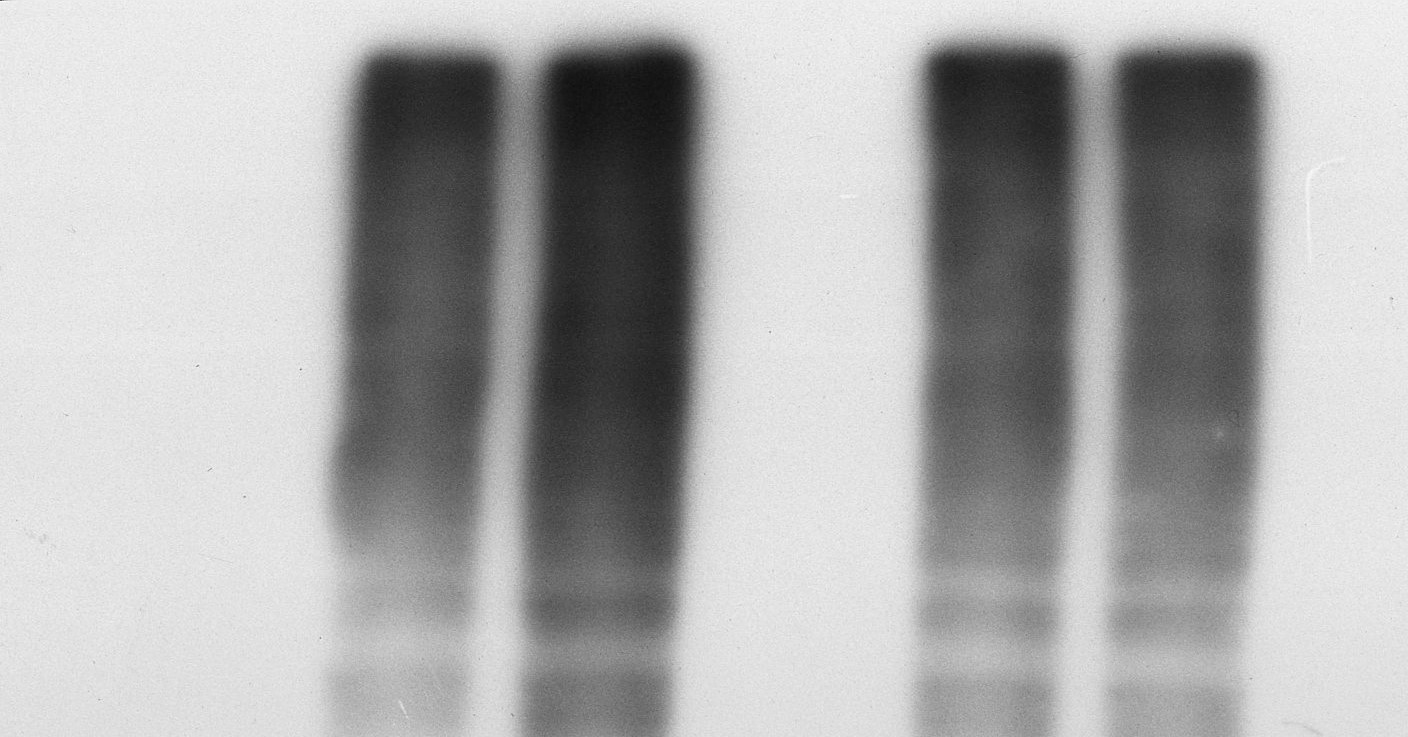

Supplement: Supplementary file 3 [file DataSheet_3.zip › fig 3/3C/fig3C Input-HA-Ub HA-K63.jpg]

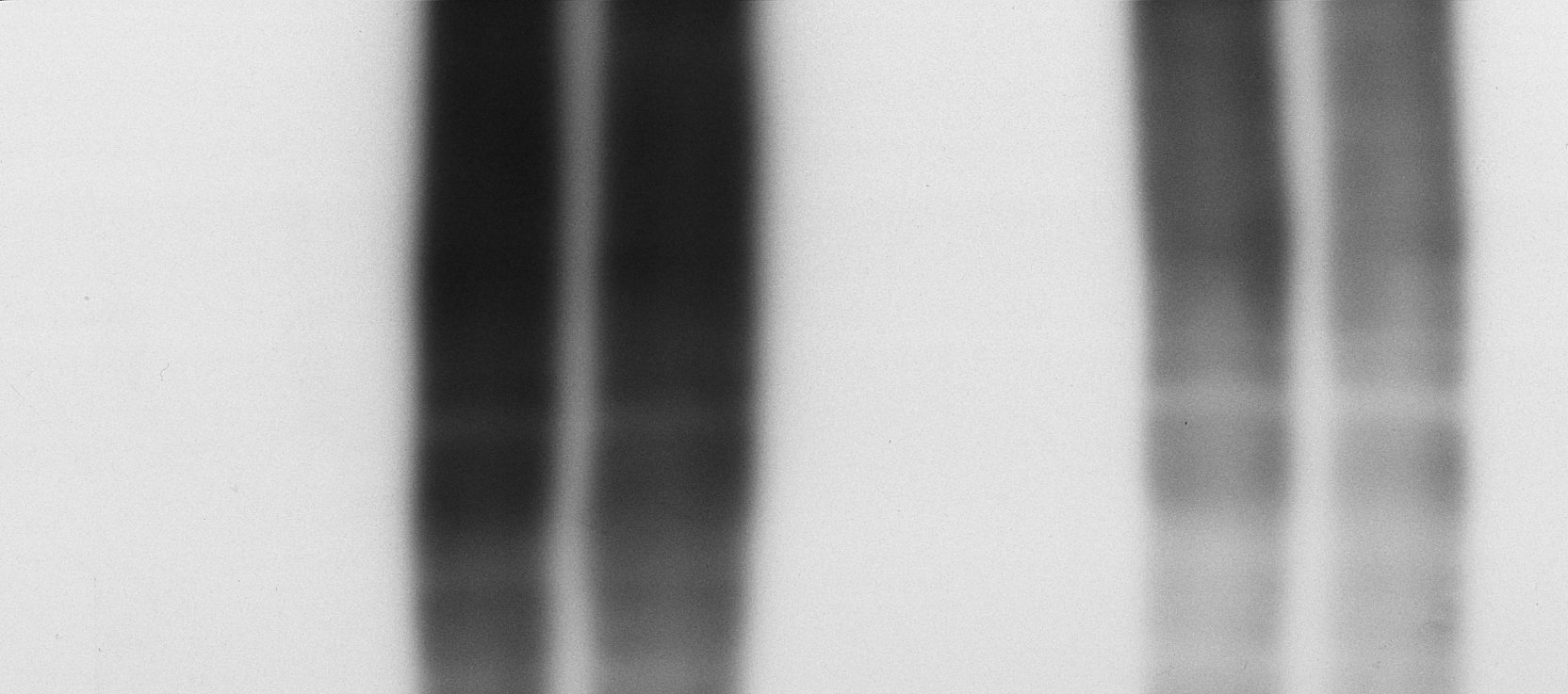

Supplement: Supplementary file 3 [file DataSheet_3.zip › fig 3/3C/fig3C Input-HA-UB K48 right 3 lane for third firgure.jpg]

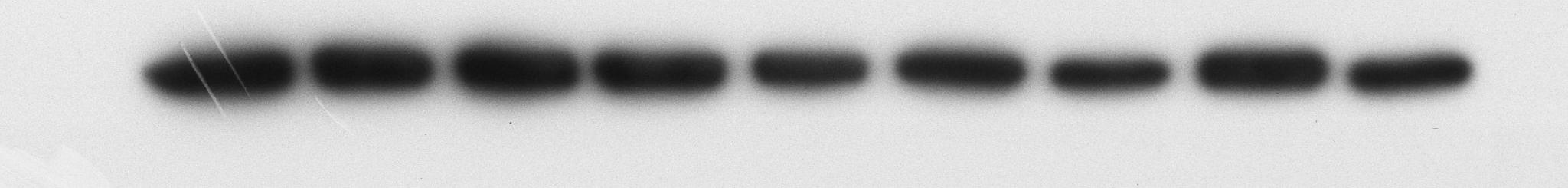

Supplement: Supplementary file 3 [file DataSheet_3.zip › fig 3/3C/fig3C IP Flag-TBK1 mid 3 lane for second figure right 3 lane for third figure.jpg]

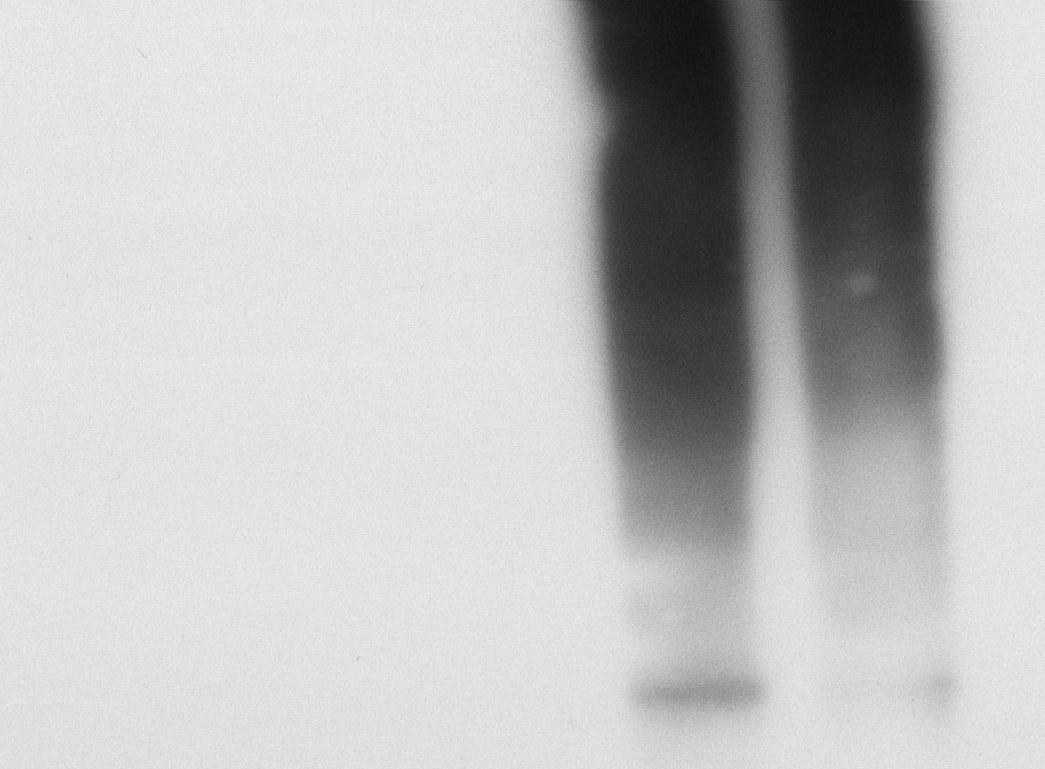

Supplement: Supplementary file 3 [file DataSheet_3.zip › fig 3/3C/fig3C IP HA-K48 for third figure.jpg]

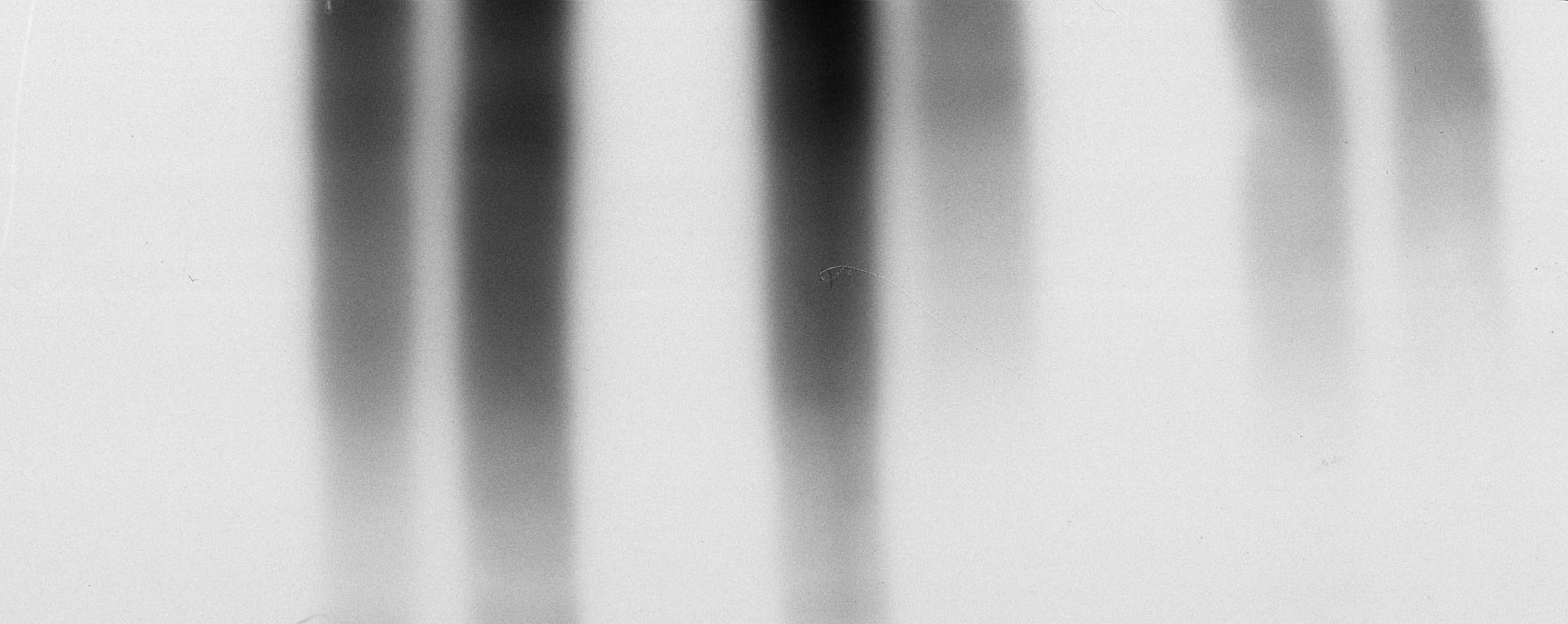

Supplement: Supplementary file 3 [file DataSheet_3.zip › fig 3/3C/fig3C IP HA-UB K63 mid 3 lane for second figure right 3 lane for third figure.jpg]

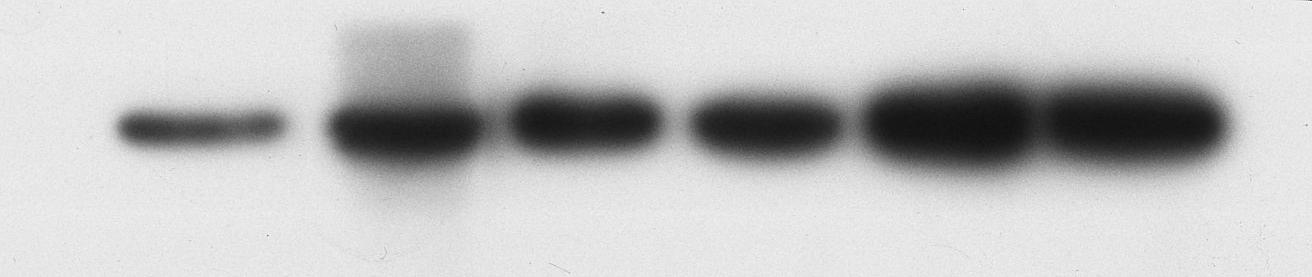

Supplement: Supplementary file 3 [file DataSheet_3.zip › fig 3/3C/fig3C IP-Flag-TBK1 left 3 lane for first figure.jpg]

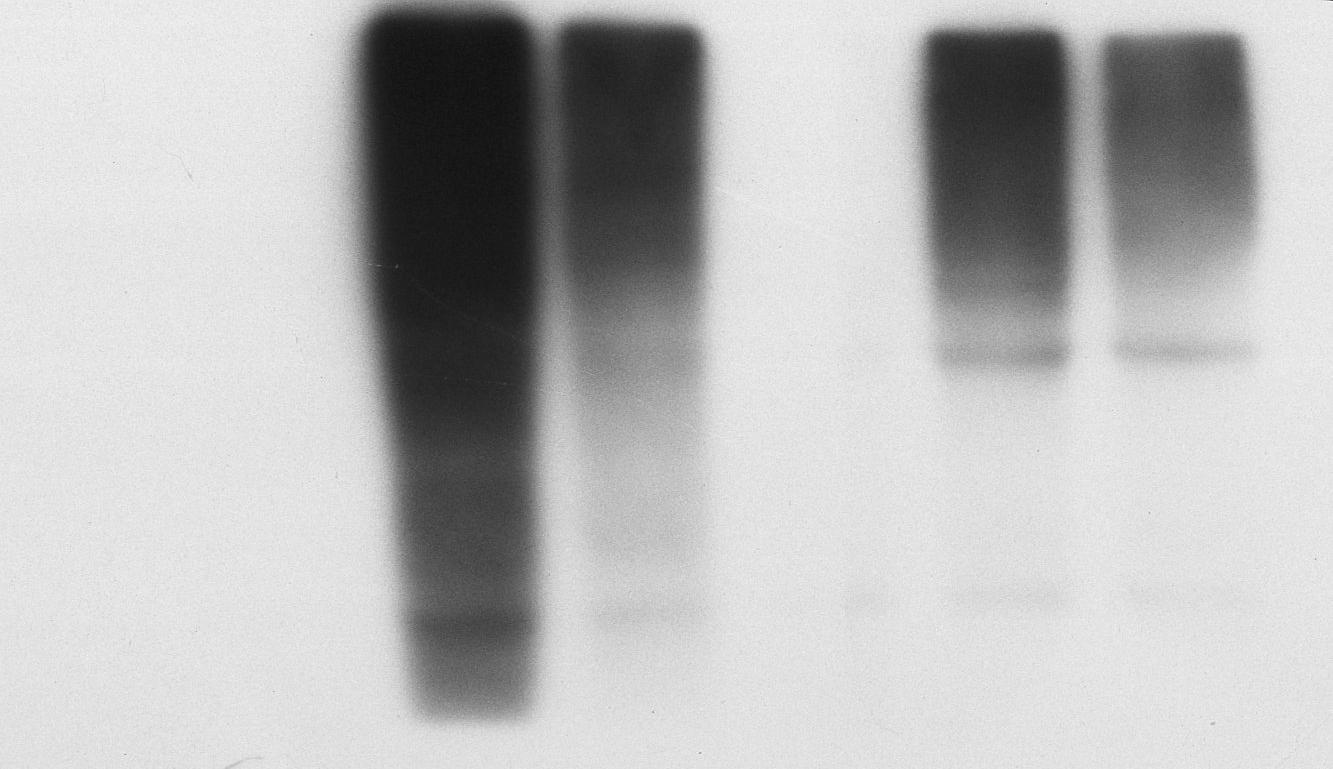

Supplement: Supplementary file 3 [file DataSheet_3.zip › fig 3/3C/fig3C IP-HA-Ub left 3 lane for first figure.jpg]

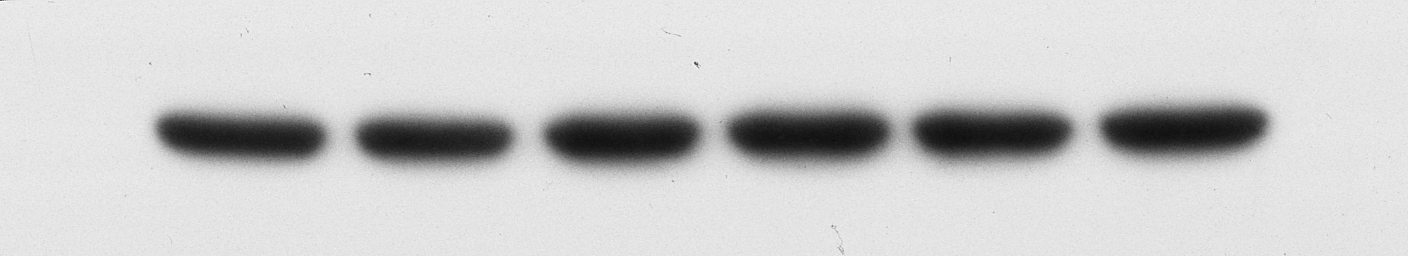

Supplement: Supplementary file 3 [file DataSheet_3.zip › fig 3/3D/fig3D actin008.jpg]

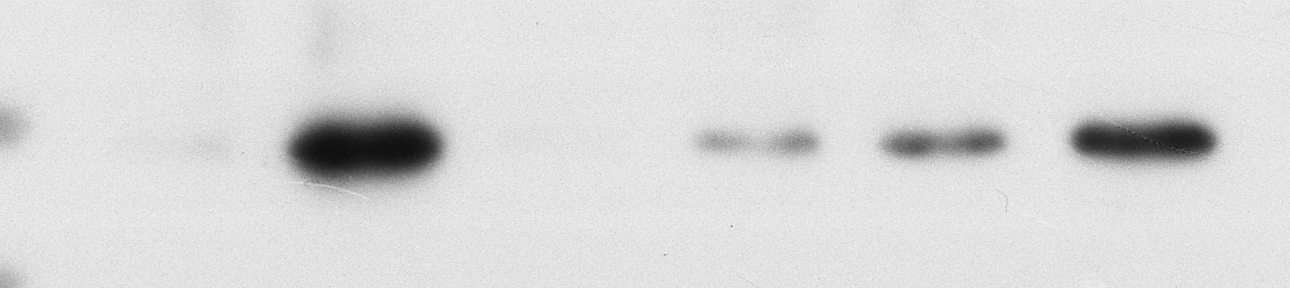

Supplement: Supplementary file 3 [file DataSheet_3.zip › fig 3/3D/fig3D HA-A151R.jpg]

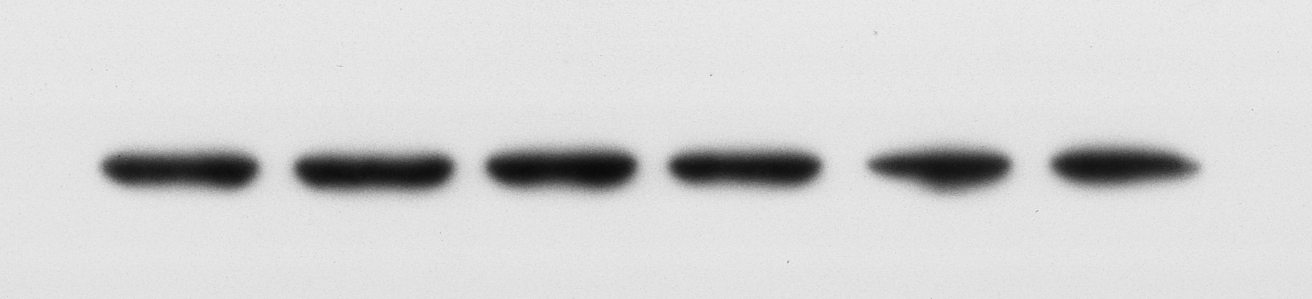

Supplement: Supplementary file 3 [file DataSheet_3.zip › fig 3/3D/fig3D IRF3.jpg]

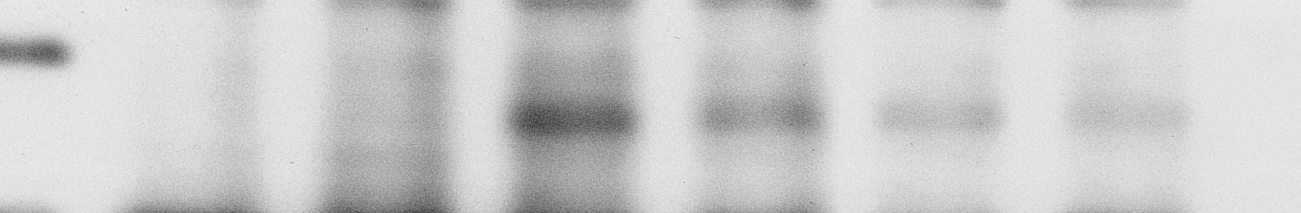

Supplement: Supplementary file 3 [file DataSheet_3.zip › fig 3/3D/fig3D p-IRF3.jpg]

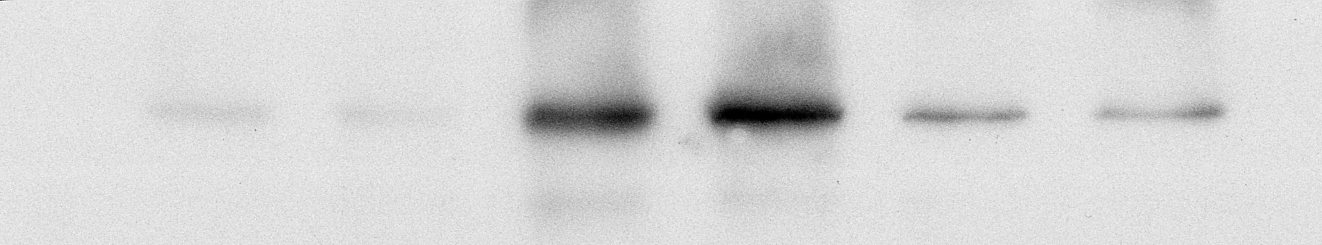

Supplement: Supplementary file 3 [file DataSheet_3.zip › fig 3/3D/fig3D p-P65.jpg]

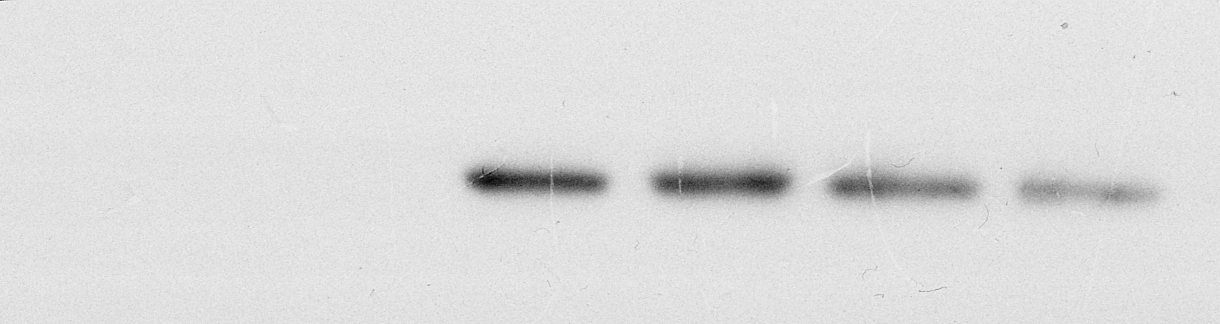

Supplement: Supplementary file 3 [file DataSheet_3.zip › fig 3/3D/fig3D p-TBK1.jpg]

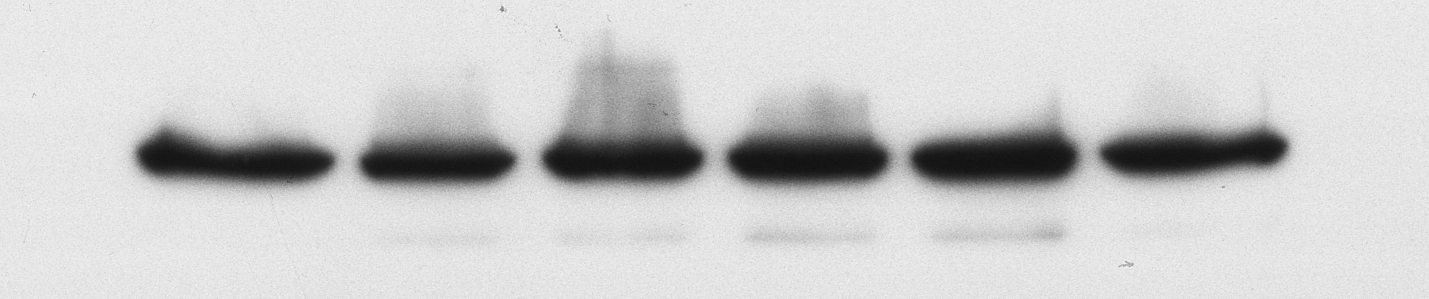

Supplement: Supplementary file 3 [file DataSheet_3.zip › fig 3/3D/fig3D P65012.jpg]

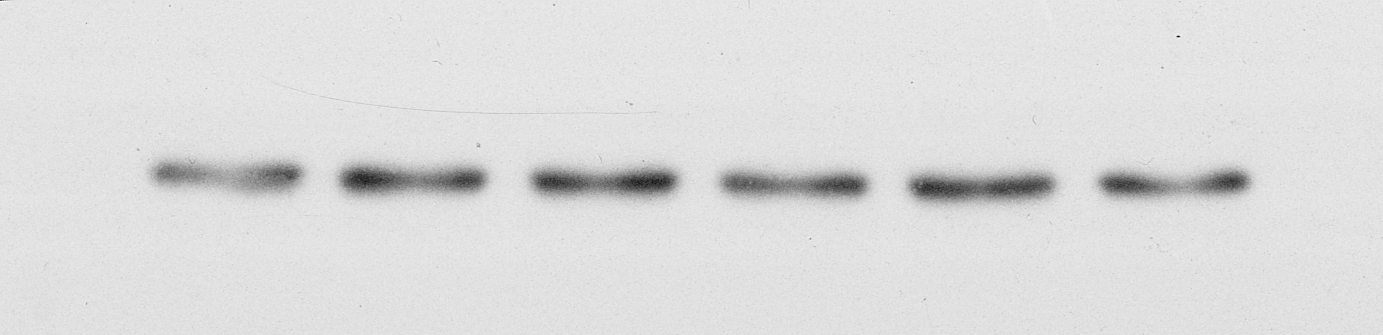

Supplement: Supplementary file 3 [file DataSheet_3.zip › fig 3/3D/fig3D TBK1.jpg]

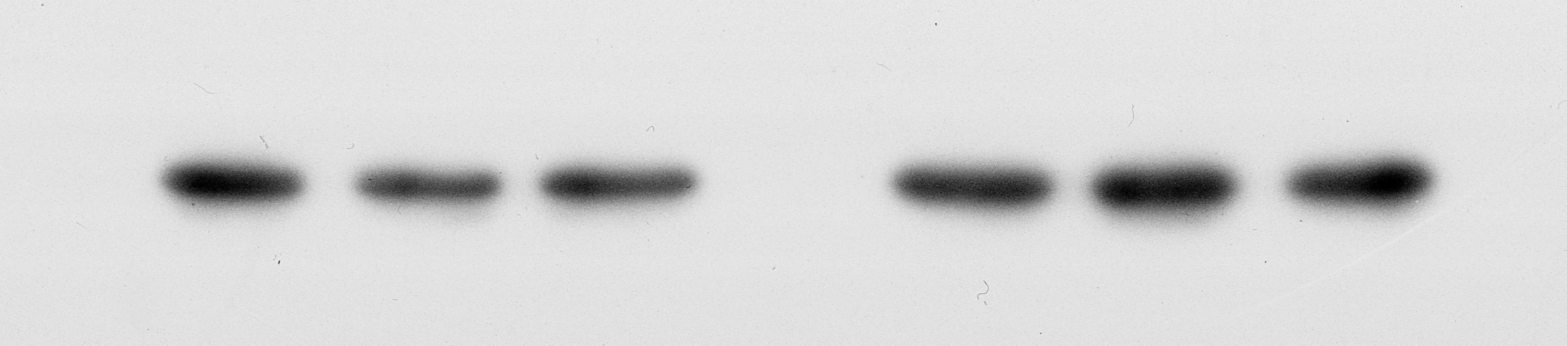

Supplement: Supplementary file 3 [file DataSheet_3.zip › fig 3/3E/actin020.jpg]

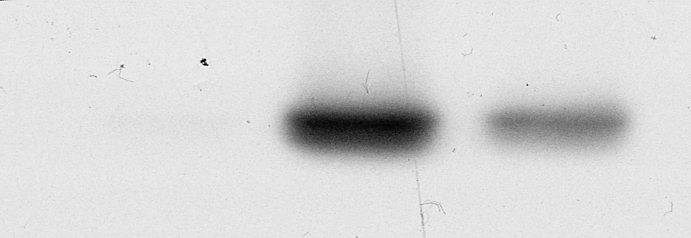

Supplement: Supplementary file 3 [file DataSheet_3.zip › fig 3/3E/Input p-TBK1 k63 for second figure.jpg]

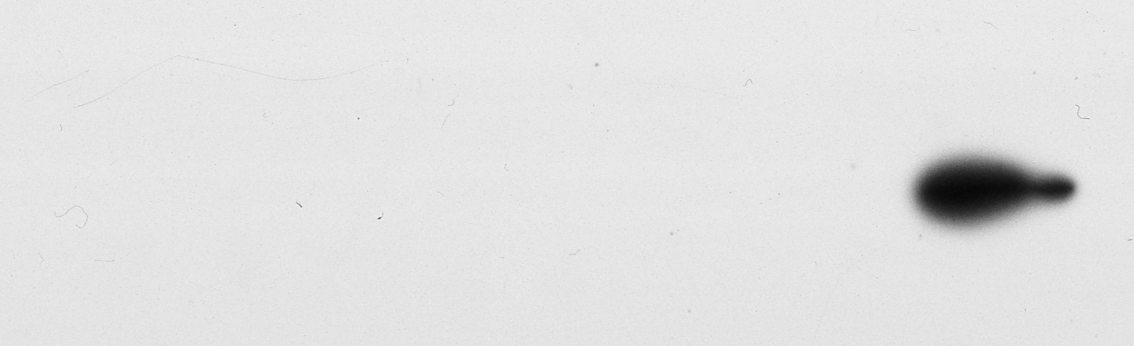

Supplement: Supplementary file 3 [file DataSheet_3.zip › fig 3/3E/Input-HA-A151R for second figure.jpg]

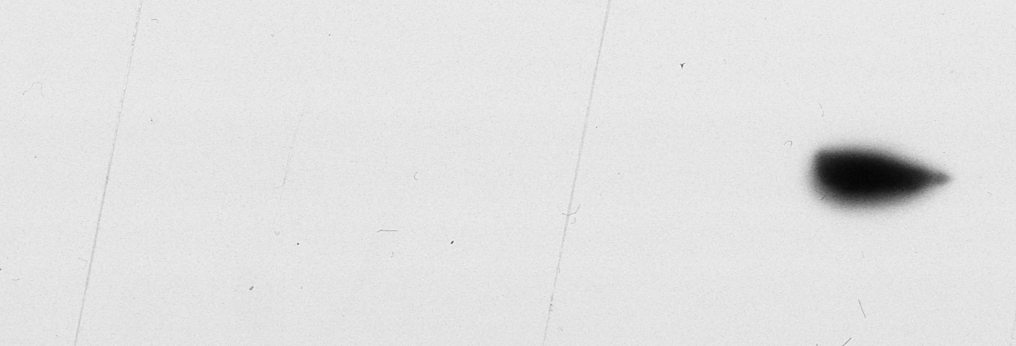

Supplement: Supplementary file 3 [file DataSheet_3.zip › fig 3/3E/Input-HA-A151R ub for first figure.jpg]

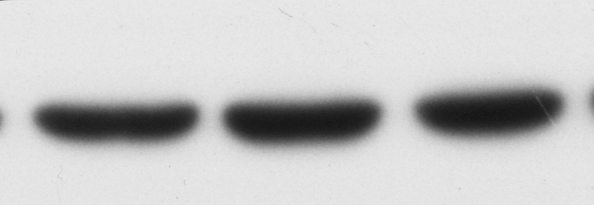

Supplement: Supplementary file 3 [file DataSheet_3.zip › fig 3/3E/Input-K63-TBK1 for second figure.jpg]

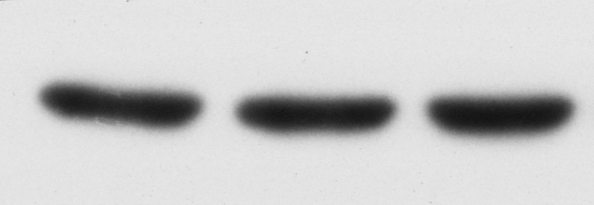

Supplement: Supplementary file 3 [file DataSheet_3.zip › fig 3/3E/Input-UB-TBK1 for first figure.jpg]

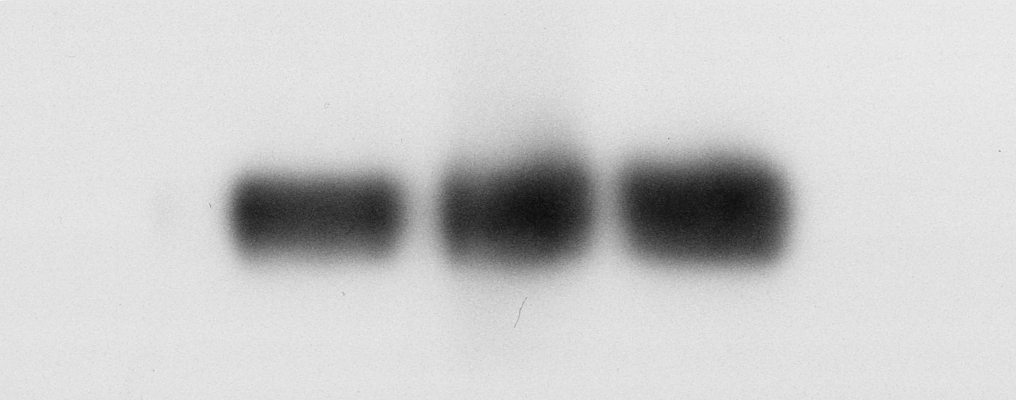

Supplement: Supplementary file 3 [file DataSheet_3.zip › fig 3/3E/IP-TBK1 K63 for second figure.jpg]

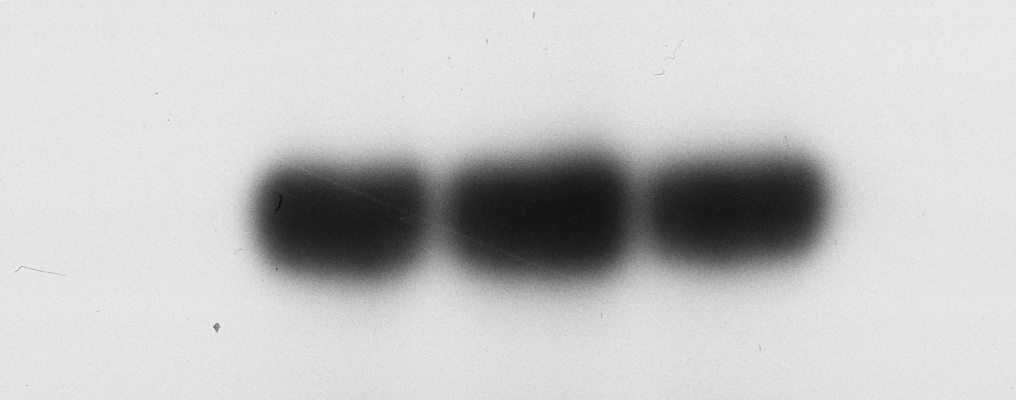

Supplement: Supplementary file 3 [file DataSheet_3.zip › fig 3/3E/IP-TBK1 UB for first figure.jpg]

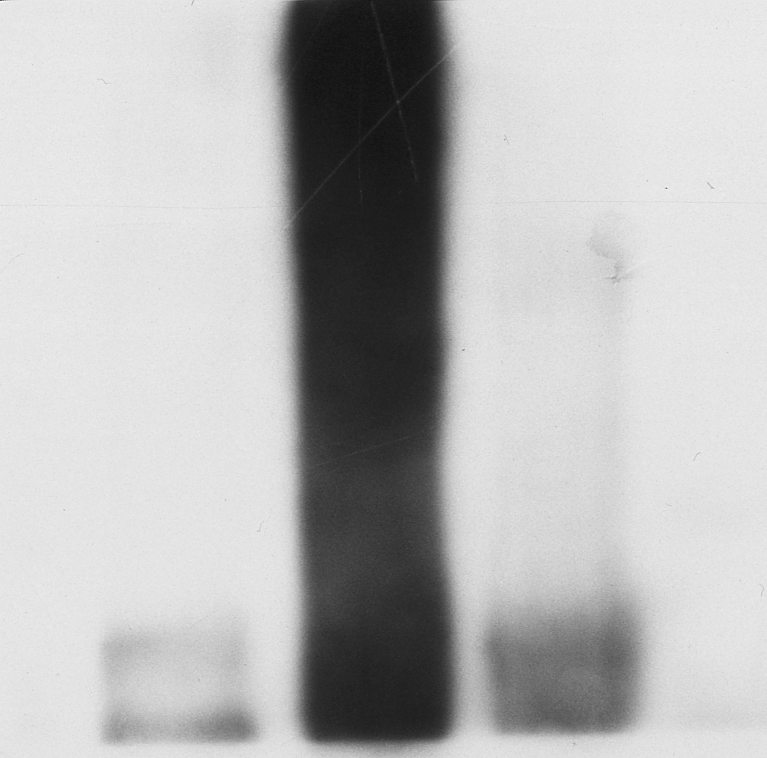

Supplement: Supplementary file 3 [file DataSheet_3.zip › fig 3/3E/IP-TBK1-K63 for second.jpg]

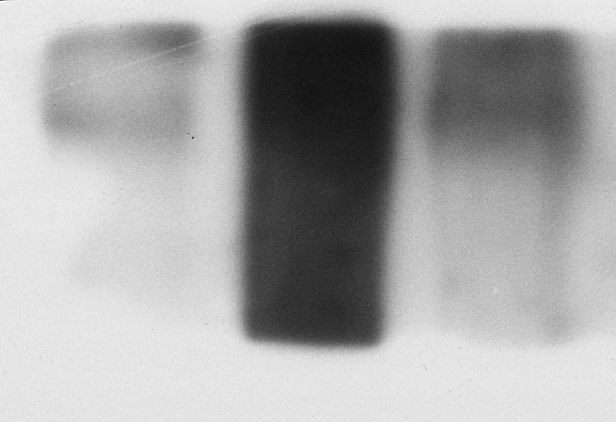

Supplement: Supplementary file 3 [file DataSheet_3.zip › fig 3/3E/IP-TBK1-UB for first.jpg]

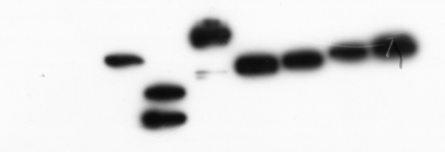

Supplement: Supplementary file 4 [file DataSheet_4.zip › fig 4/4A/fig4A IP-Flag.jpg]

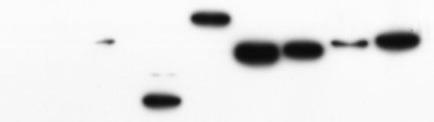

Supplement: Supplementary file 4 [file DataSheet_4.zip › fig 4/4A/fig4A Input-Flag.jpg]

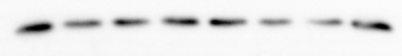

Supplement: Supplementary file 4 [file DataSheet_4.zip › fig 4/4A/fig4A Input-HA-A151R.jpg]

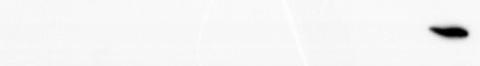

Supplement: Supplementary file 4 [file DataSheet_4.zip › fig 4/4A/fig4A IP-HA-A151R.jpg]

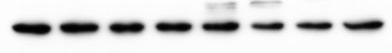

Supplement: Supplementary file 4 [file DataSheet_4.zip › fig 4/4A/fig4A a┬-actin.jpg]

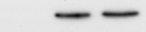

Supplement: Supplementary file 4 [file DataSheet_4.zip › fig 4/4B/fig4B Input Flag-A151R left figure.jpg]

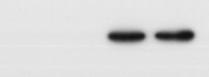

Supplement: Supplementary file 4 [file DataSheet_4.zip › fig 4/4B/fig4B Input FLAG-TRAF6 right figure.jpg]

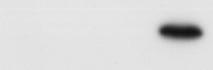

Supplement: Supplementary file 4 [file DataSheet_4.zip › fig 4/4B/fig4B Input HA-A151R right figure.jpg]

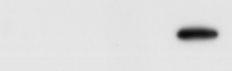

Supplement: Supplementary file 4 [file DataSheet_4.zip › fig 4/4B/fig4B Input HA-TRAF6 left figure.jpg]

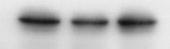

Supplement: Supplementary file 4 [file DataSheet_4.zip › fig 4/4B/fig4B Input a┬-actin left figure.jpg]

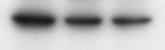

Supplement: Supplementary file 4 [file DataSheet_4.zip › fig 4/4B/fig4B Input a┬-actin right figure.jpg]

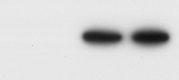

Supplement: Supplementary file 4 [file DataSheet_4.zip › fig 4/4B/fig4B IP FLAG-A151R left figure.jpg]

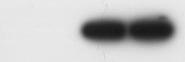

Supplement: Supplementary file 4 [file DataSheet_4.zip › fig 4/4B/fig4B IP FLAG-TRAF6 right figure.jpg]

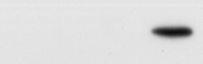

Supplement: Supplementary file 4 [file DataSheet_4.zip › fig 4/4B/fig4B IP HA-A151R right figure.jpg]

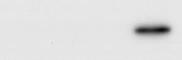

Supplement: Supplementary file 4 [file DataSheet_4.zip › fig 4/4B/fig4B IP HA-TRAF6 left figure.jpg]

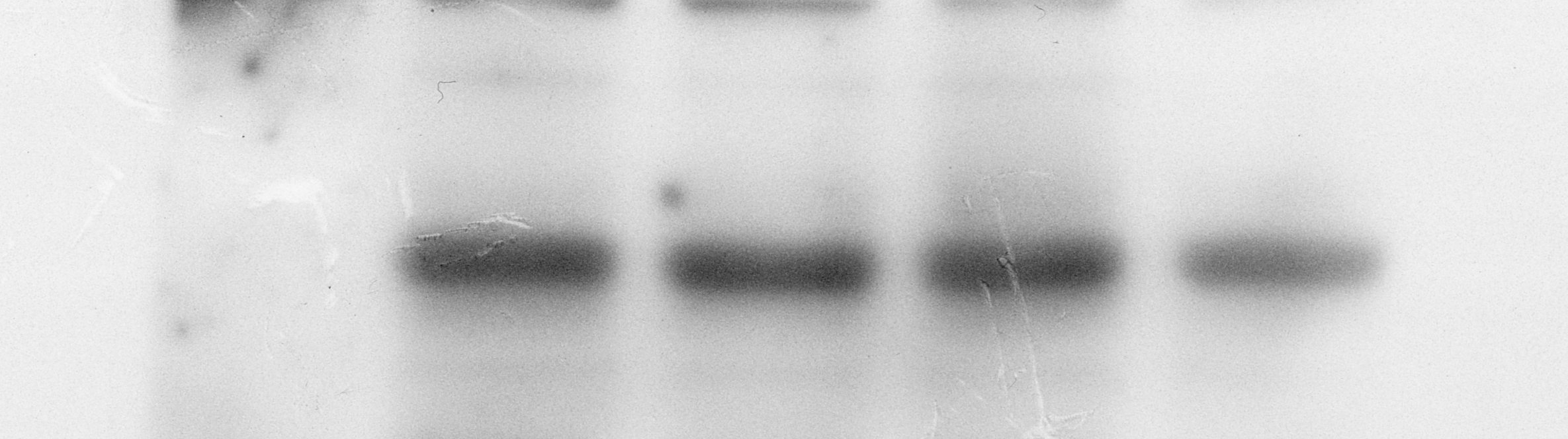

Supplement: Supplementary file 4 [file DataSheet_4.zip › fig 4/4C/FIG4C Input TRAF6.jpg]

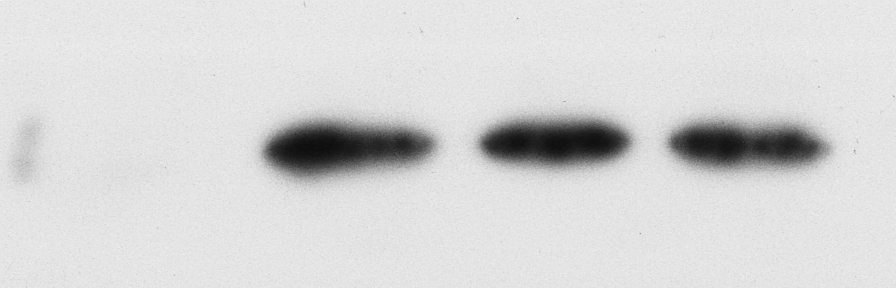

Supplement: Supplementary file 4 [file DataSheet_4.zip › fig 4/4C/FIG4C Input-Flag-A151R.jpg]

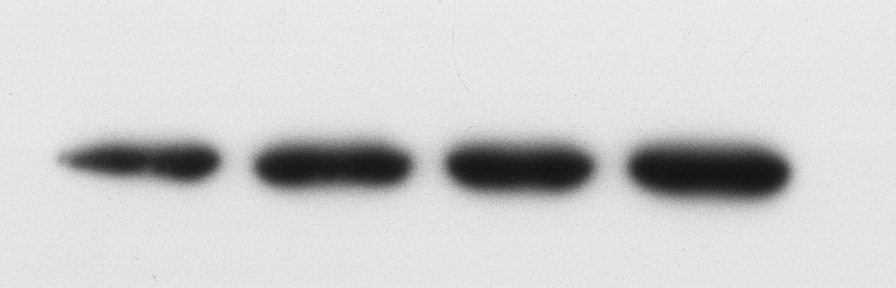

Supplement: Supplementary file 4 [file DataSheet_4.zip › fig 4/4C/FIG4C Input-a┬-actin028.jpg]

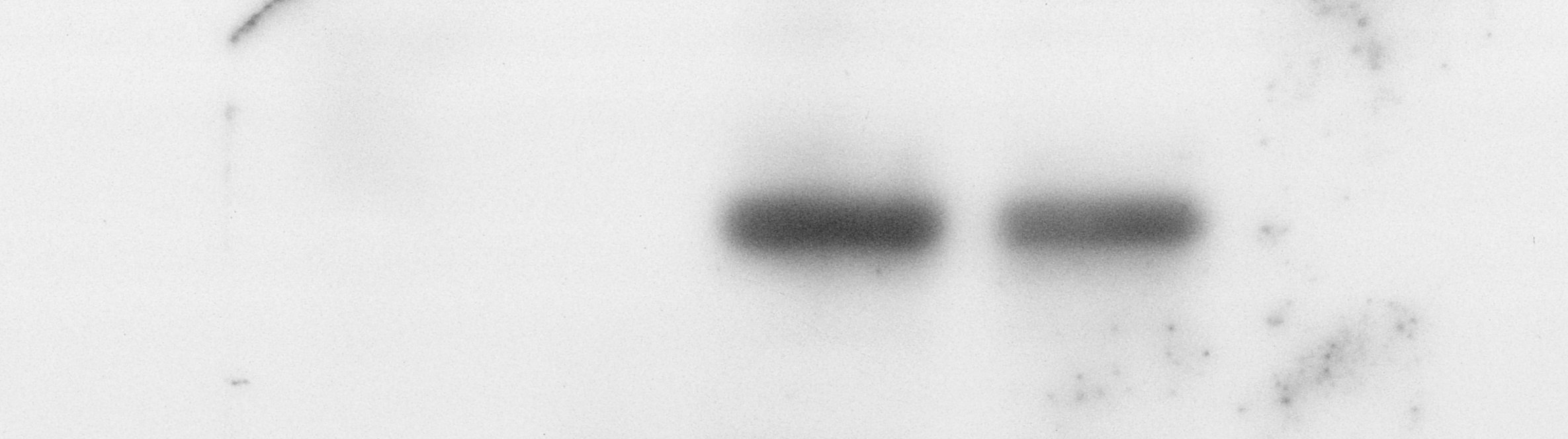

Supplement: Supplementary file 4 [file DataSheet_4.zip › fig 4/4C/FIG4C IP TRAF6.jpg]

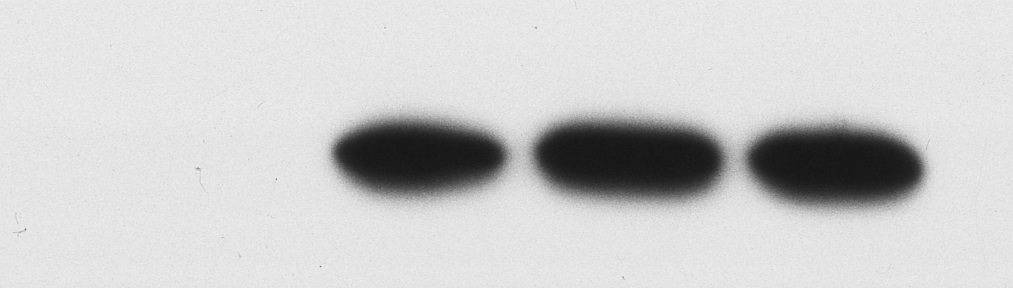

Supplement: Supplementary file 4 [file DataSheet_4.zip › fig 4/4C/FIG4C IP-Flag-A151R029.jpg]

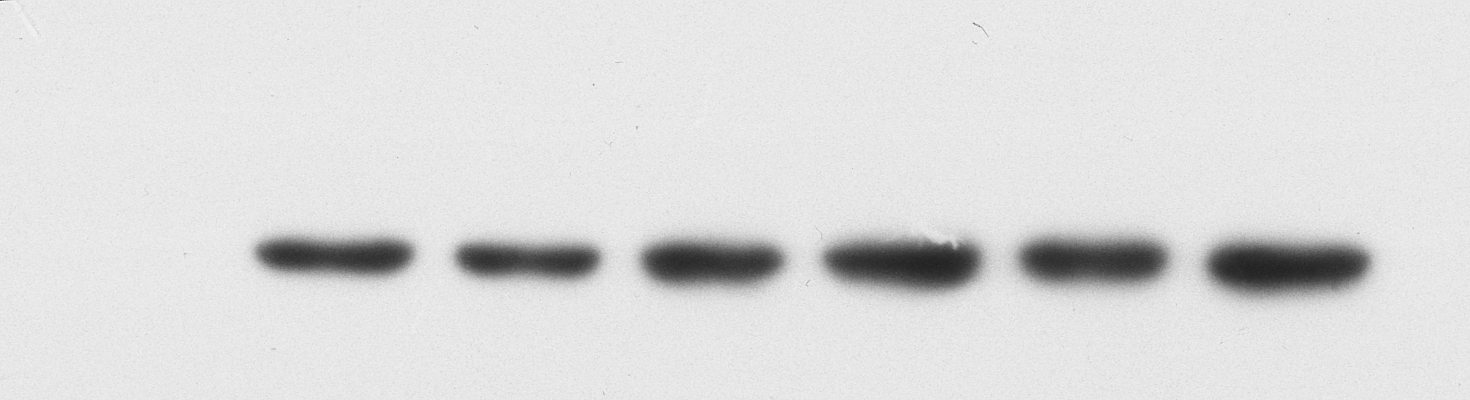

Supplement: Supplementary file 4 [file DataSheet_4.zip › fig 4/4F/FIG4F ACTIN.jpg]

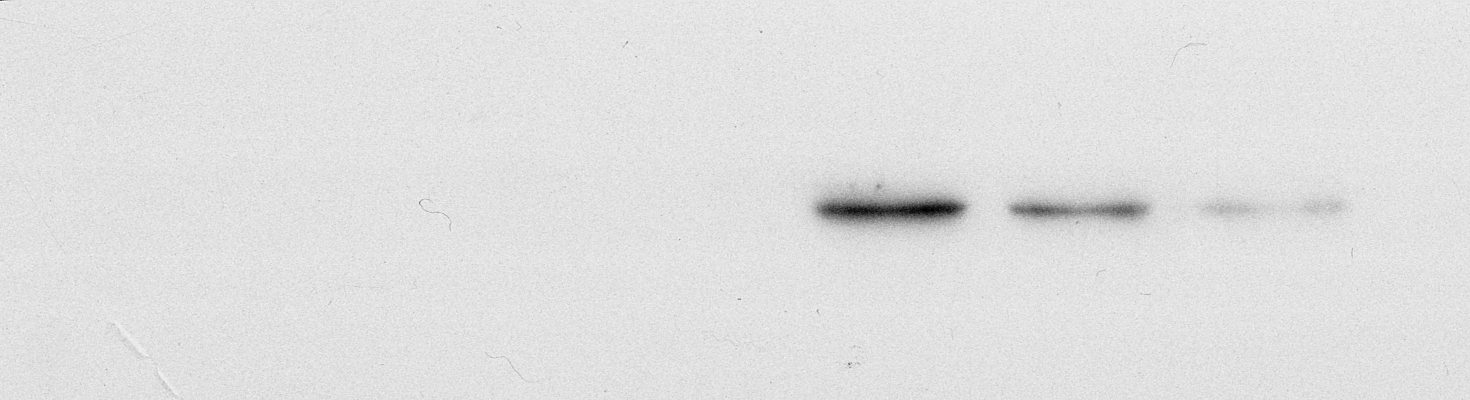

Supplement: Supplementary file 4 [file DataSheet_4.zip › fig 4/4F/FIG4F p-TBK1.jpg]

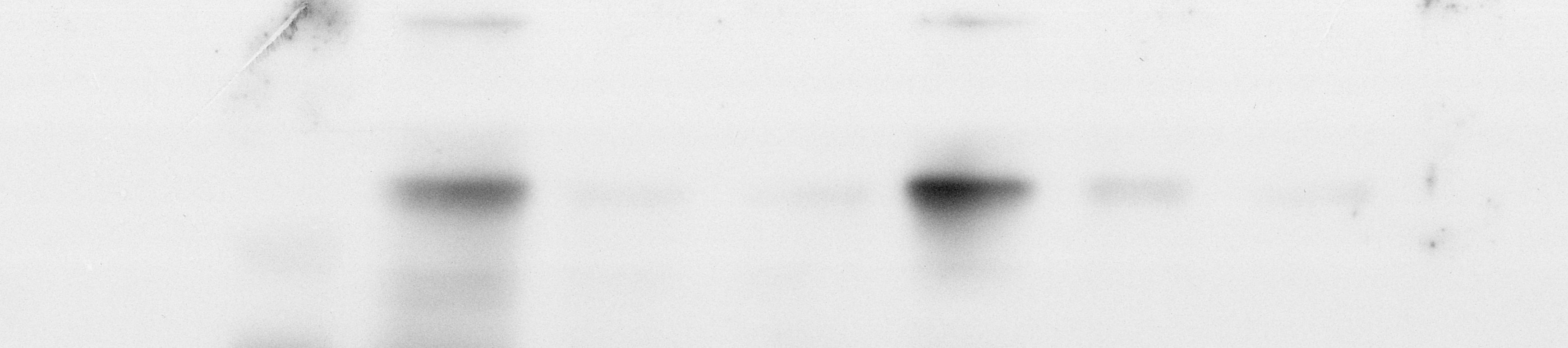

Supplement: Supplementary file 4 [file DataSheet_4.zip › fig 4/4F/FIG4F siTRAF6-1.jpg]

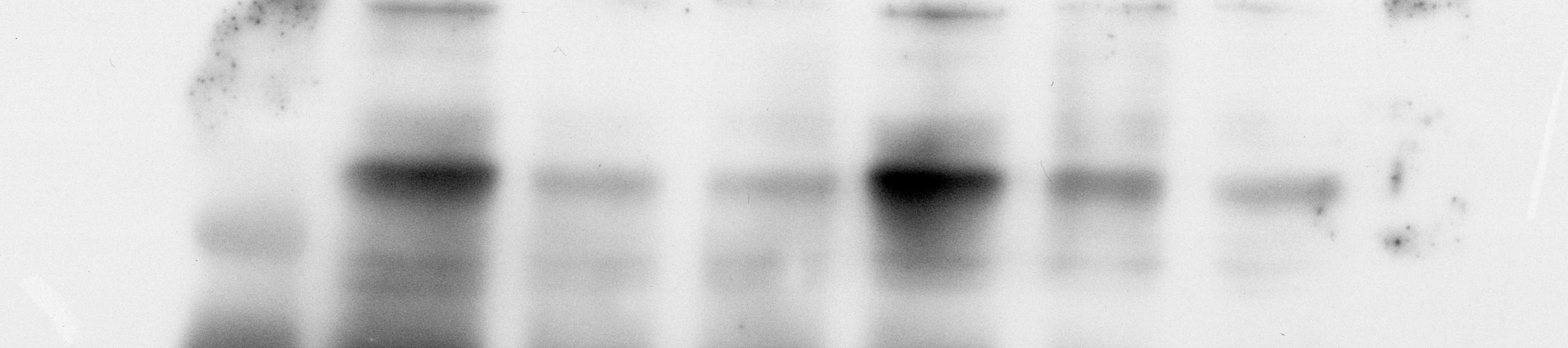

Supplement: Supplementary file 4 [file DataSheet_4.zip › fig 4/4F/FIG4F siTRAF6-2.jpg]

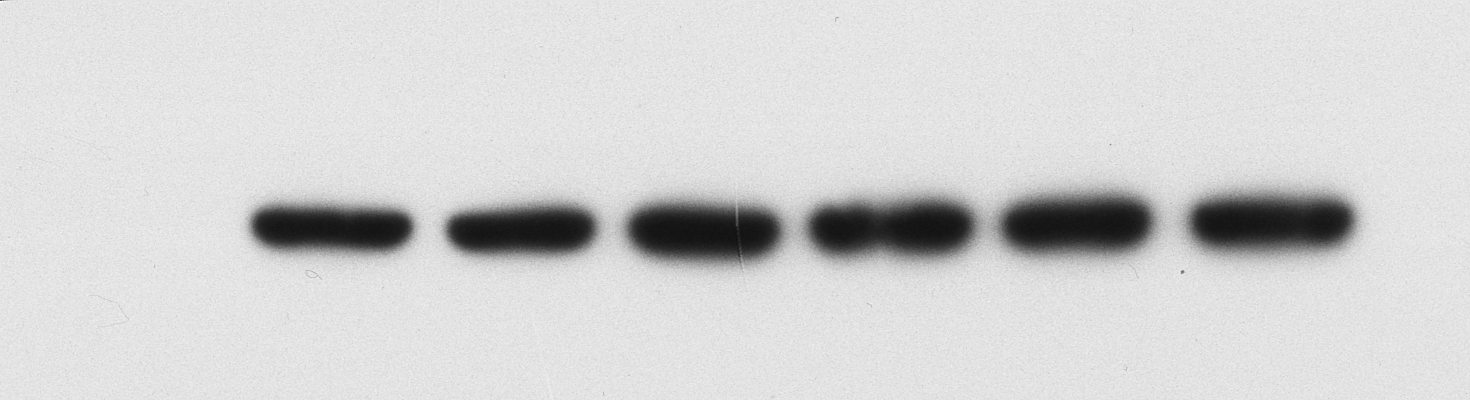

Supplement: Supplementary file 4 [file DataSheet_4.zip › fig 4/4F/FIG4F TBK1.jpg]

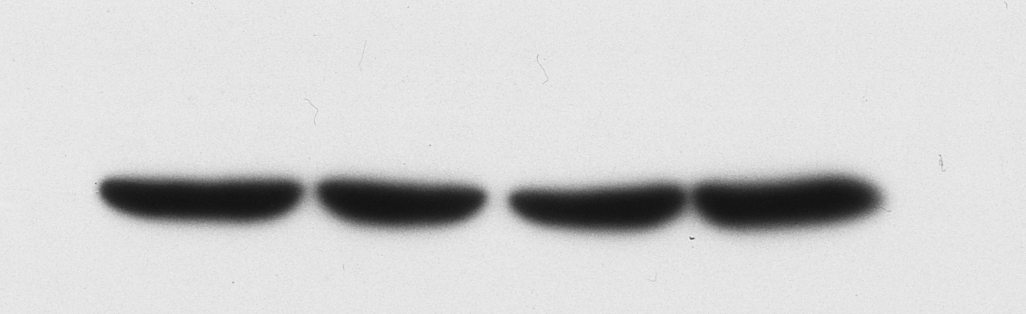

Supplement: Supplementary file 4 [file DataSheet_4.zip › fig 4/4G/fig4G actin008.jpg]

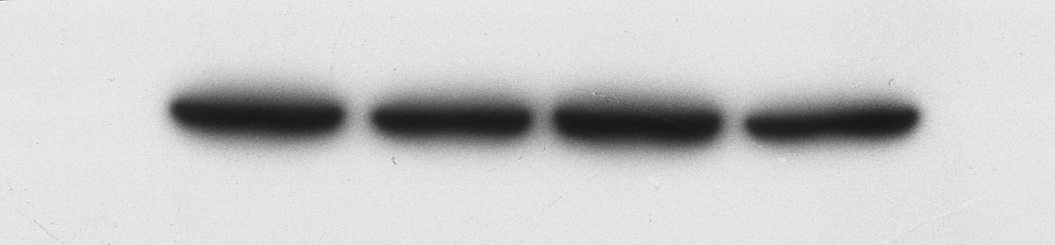

Supplement: Supplementary file 4 [file DataSheet_4.zip › fig 4/4G/fig4G Input TBK1.jpg]

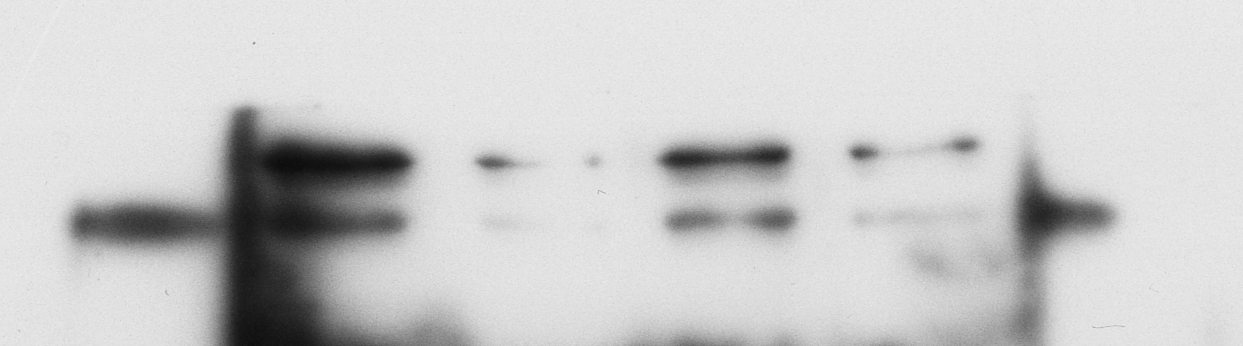

Supplement: Supplementary file 4 [file DataSheet_4.zip › fig 4/4G/fig4G Input-TRAF6.jpg]

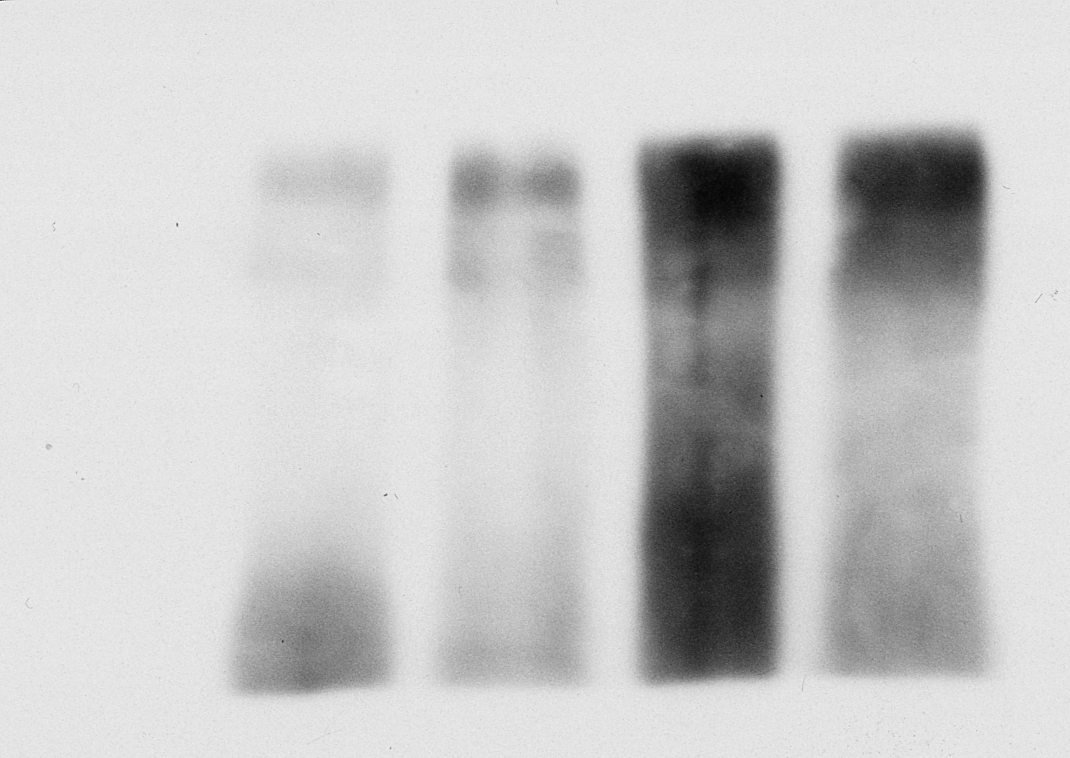

Supplement: Supplementary file 4 [file DataSheet_4.zip › fig 4/4G/fig4G IP-K63.jpg]

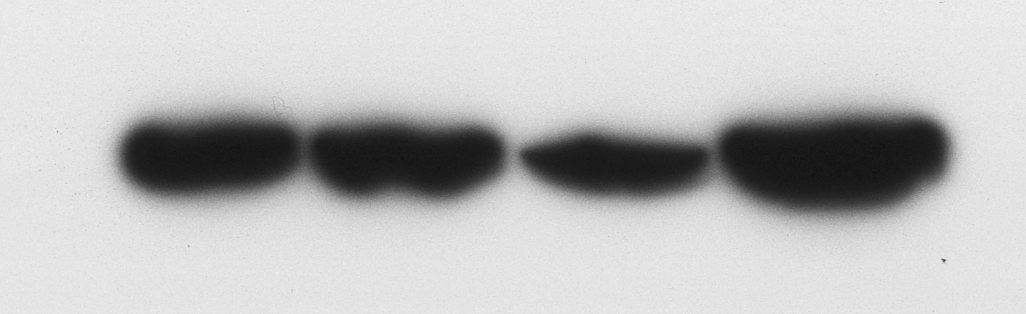

Supplement: Supplementary file 4 [file DataSheet_4.zip › fig 4/4G/fig4G IP-TBK1.jpg]

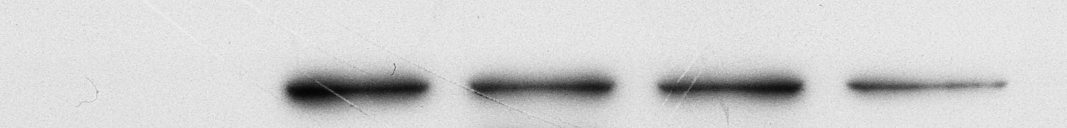

Supplement: Supplementary file 5 [file DataSheet_5.zip › fig 5/5B/fig5B Input-Flag-TBK1.jpg]

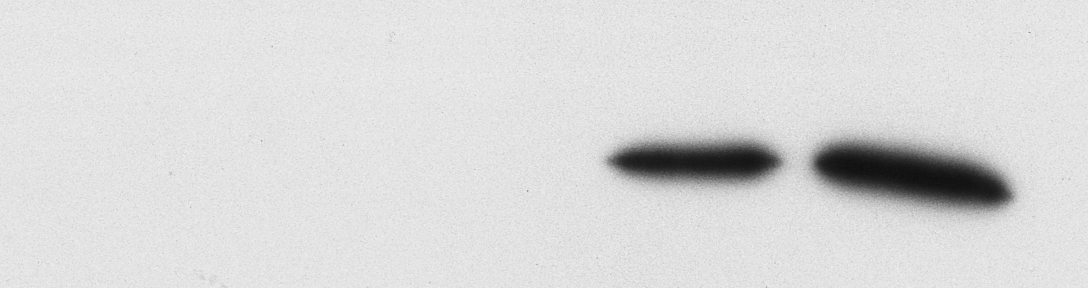

Supplement: Supplementary file 5 [file DataSheet_5.zip › fig 5/5B/fig5B Input-HA-A151R.jpg]

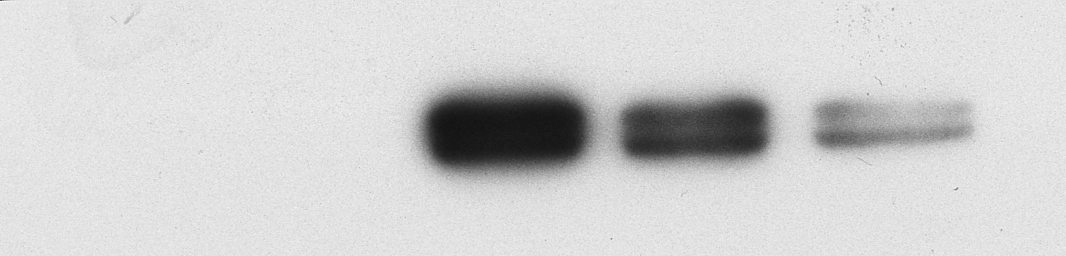

Supplement: Supplementary file 5 [file DataSheet_5.zip › fig 5/5B/fig5B Input-HA-TRAF6.jpg]

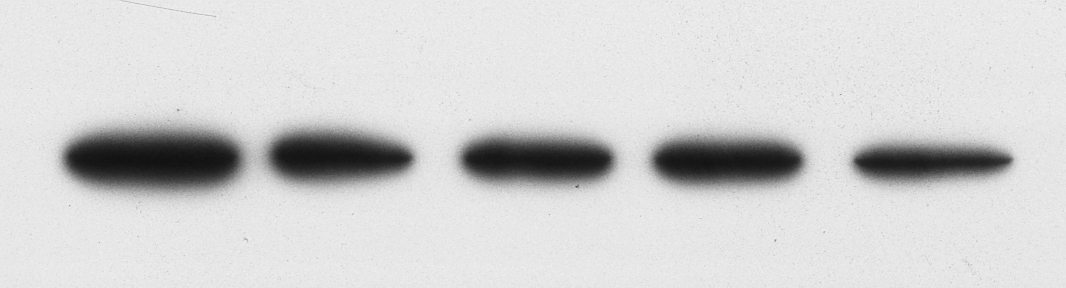

Supplement: Supplementary file 5 [file DataSheet_5.zip › fig 5/5B/fig5B Input-a┬-actin.jpg]

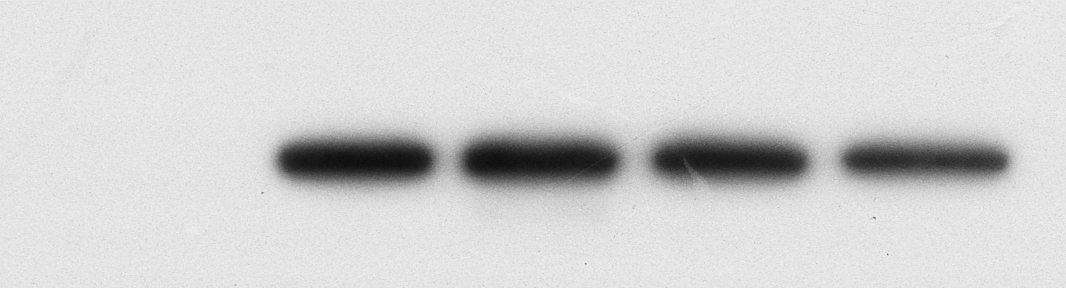

Supplement: Supplementary file 5 [file DataSheet_5.zip › fig 5/5B/fig5B IP-Flag-TBK1.jpg]

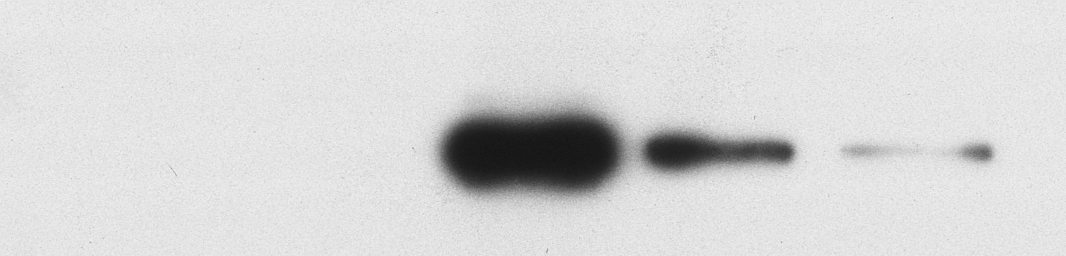

Supplement: Supplementary file 5 [file DataSheet_5.zip › fig 5/5B/fig5B IP-HA-TRAF6.jpg]

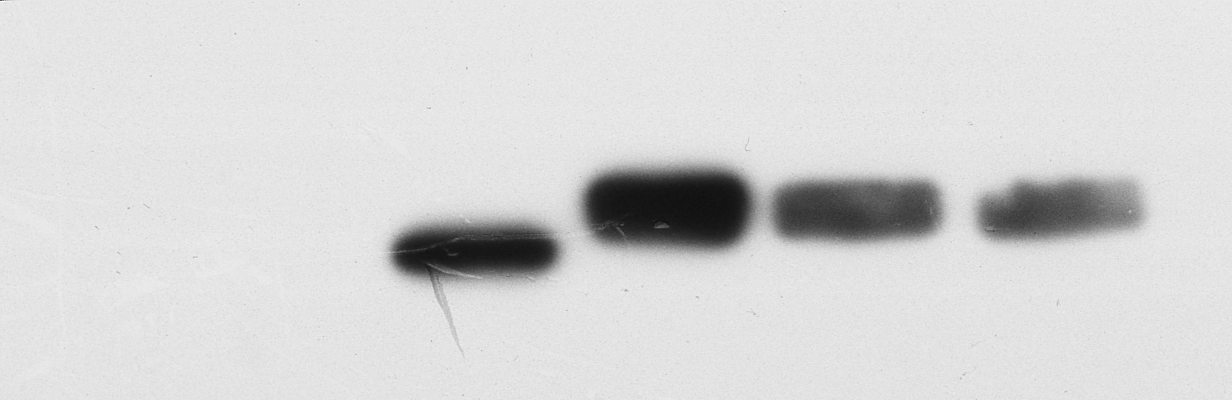

Supplement: Supplementary file 5 [file DataSheet_5.zip › fig 5/5C/fig5C Input-Flag-TRAF6.jpg]

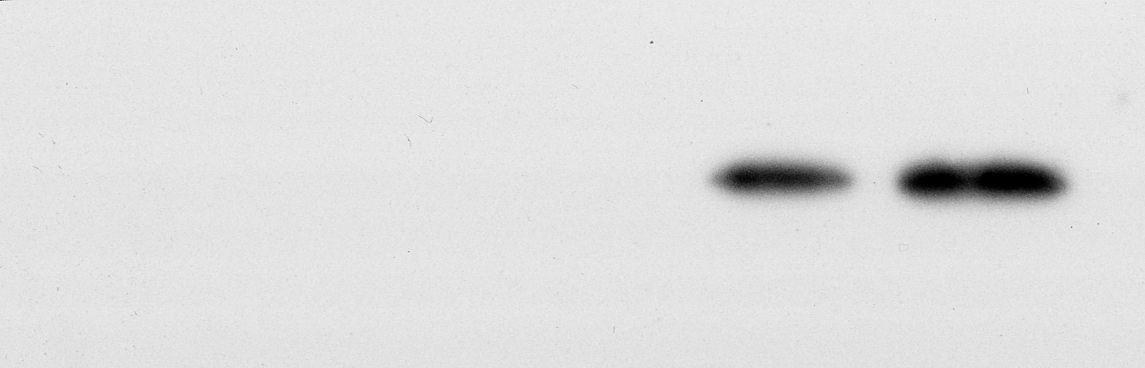

Supplement: Supplementary file 5 [file DataSheet_5.zip › fig 5/5C/fig5C Input-HA-A151R.jpg]

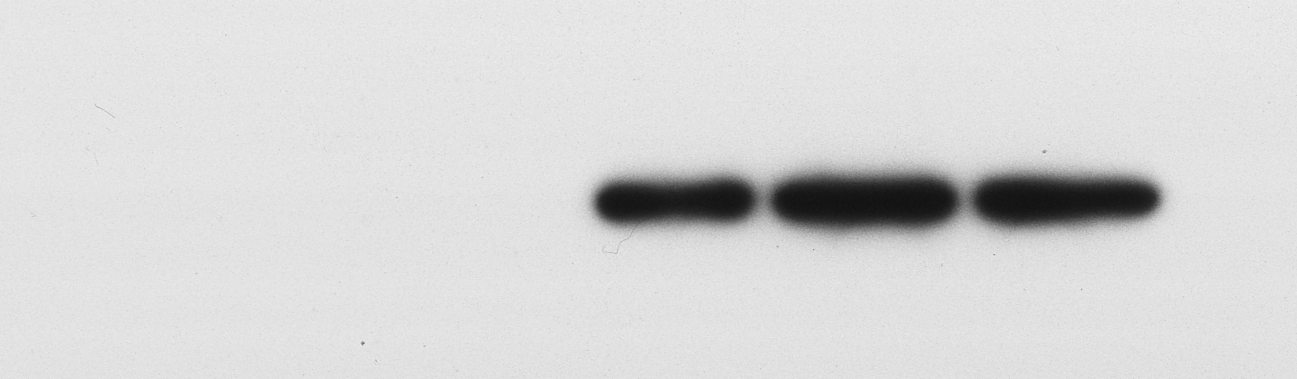

Supplement: Supplementary file 5 [file DataSheet_5.zip › fig 5/5C/fig5C Input-HA-TBK1.jpg]

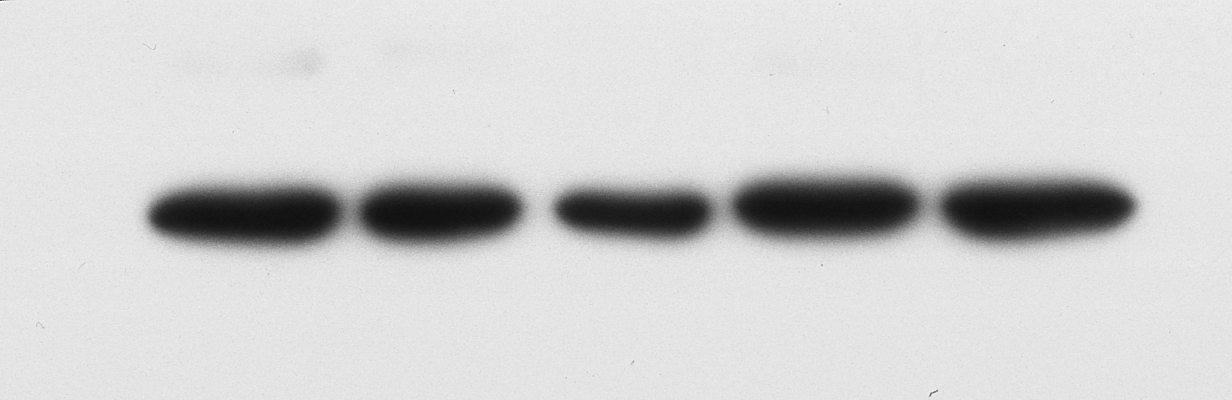

Supplement: Supplementary file 5 [file DataSheet_5.zip › fig 5/5C/fig5C Input-a┬-actin.jpg]

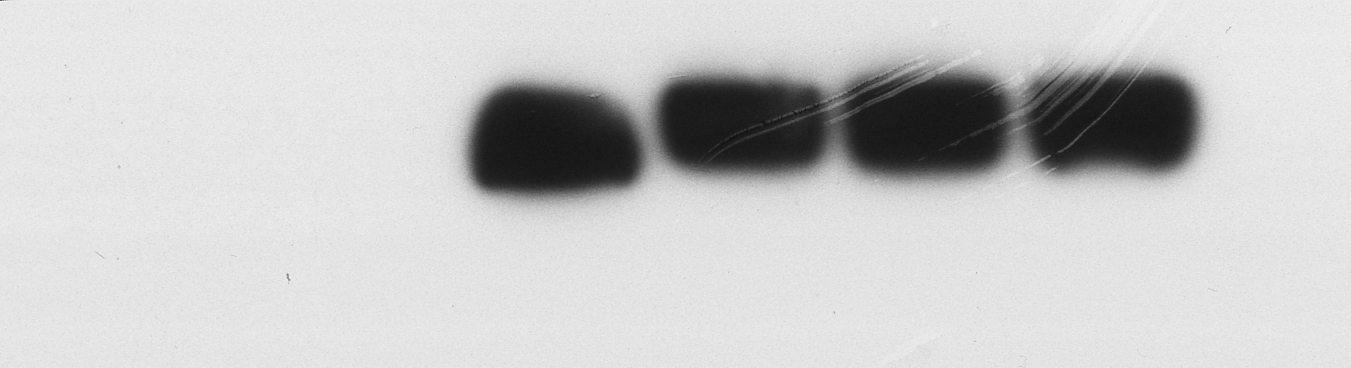

Supplement: Supplementary file 5 [file DataSheet_5.zip › fig 5/5C/fig5C IP-Flag-TRAF6.jpg]

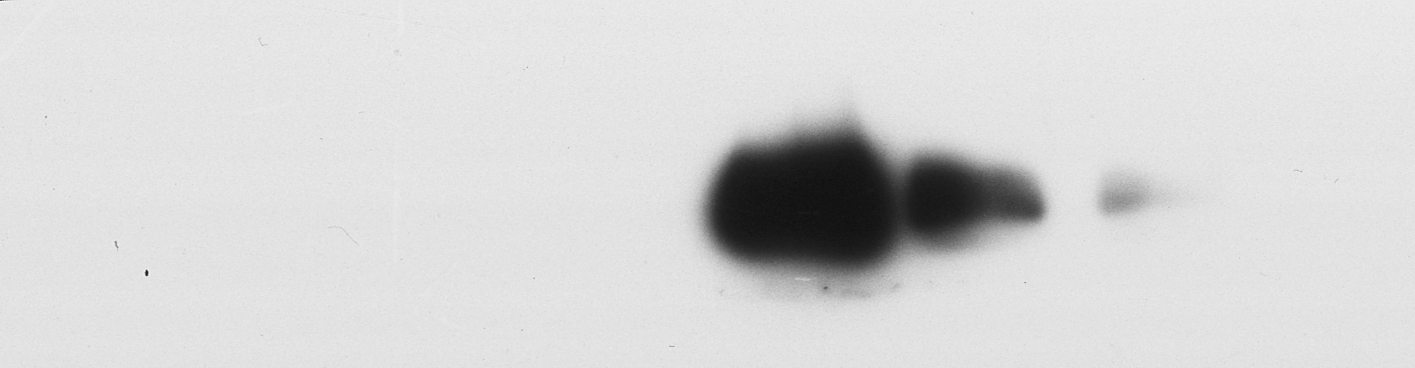

Supplement: Supplementary file 5 [file DataSheet_5.zip › fig 5/5C/fig5C IP-HA-TBK1.jpg]

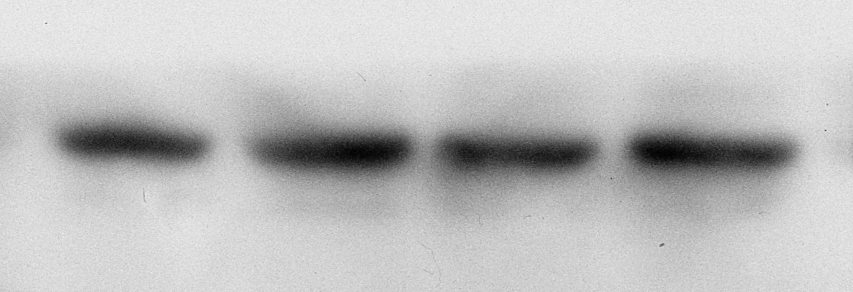

Supplement: Supplementary file 5 [file DataSheet_5.zip › fig 5/5D/fig5D Input-B-actin.jpg]

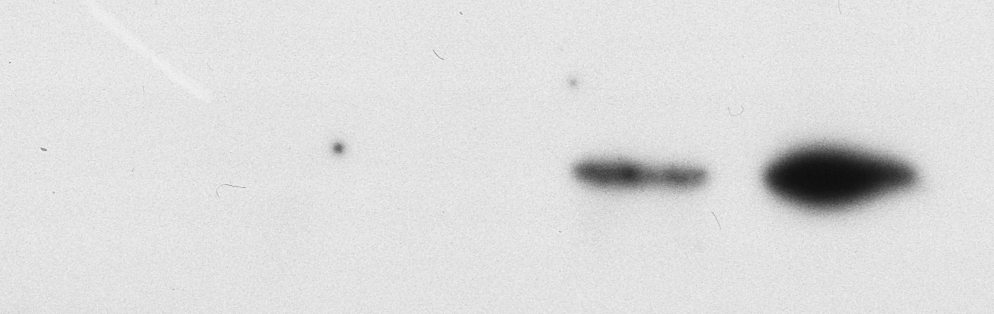

Supplement: Supplementary file 5 [file DataSheet_5.zip › fig 5/5D/fig5D Input-HA-A151R007.jpg]
