# Supplementary figures and images for: ASFV pA151R negatively regulates type I IFN production via degrading E3 ligase TRAF6 (part 2 of 2)
Source: Front Immunol. 2024 Feb 21;15:1339510. doi: 10.3389/fimmu.2024.1339510 (PMC10914938; doi:10.3389/fimmu.2024.1339510)

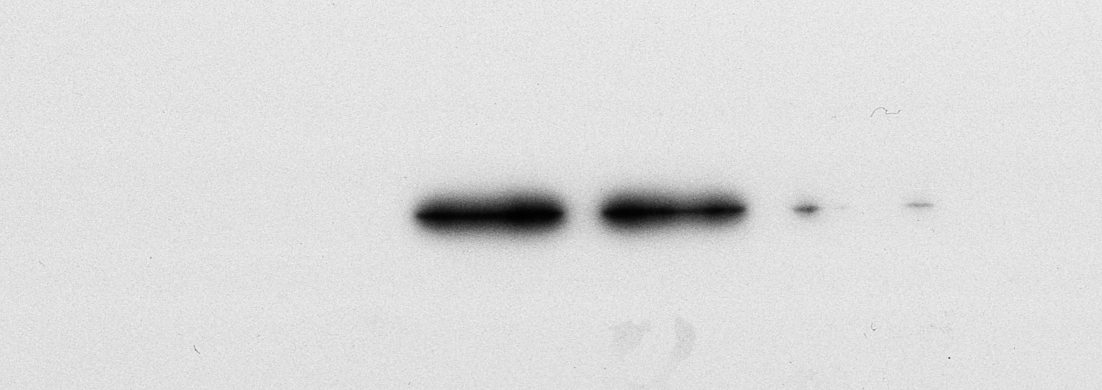

Supplement: Supplementary file 5 [file DataSheet_5.zip › fig 5/5D/fig5D Input-pTBK1011.jpg]

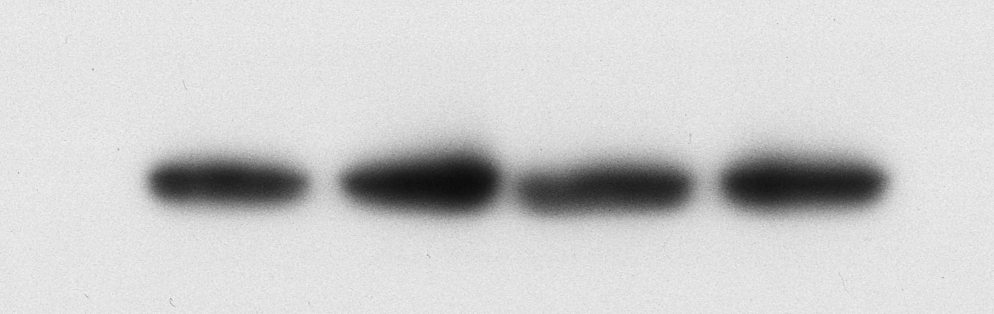

Supplement: Supplementary file 5 [file DataSheet_5.zip › fig 5/5D/fig5D Input-TBK1008.jpg]

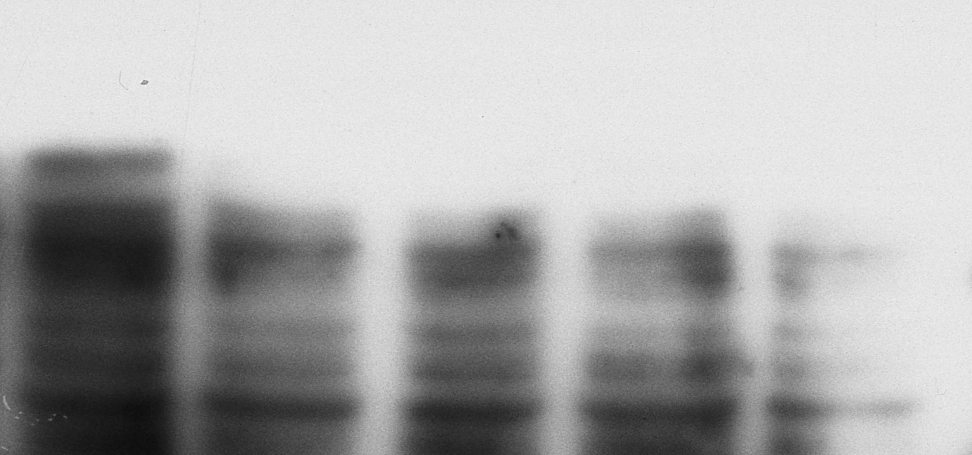

Supplement: Supplementary file 5 [file DataSheet_5.zip › fig 5/5D/fig5D Input-TRAF6 lane 2-5 TRAF6 ú¿lane 1 is makerú⌐.jpg]

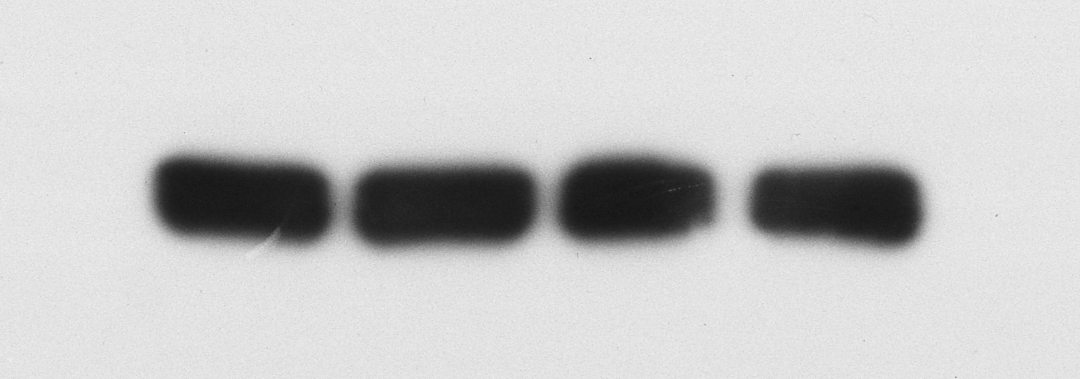

Supplement: Supplementary file 5 [file DataSheet_5.zip › fig 5/5D/fig5D IP-TBK1005.jpg]

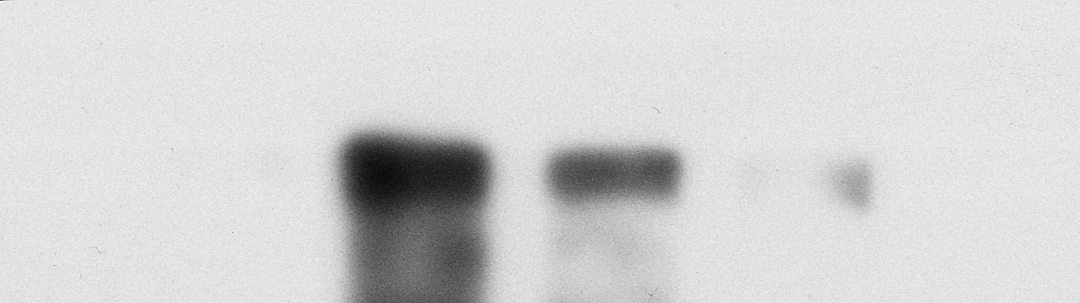

Supplement: Supplementary file 5 [file DataSheet_5.zip › fig 5/5D/fig5D IP-TRAF6.jpg]

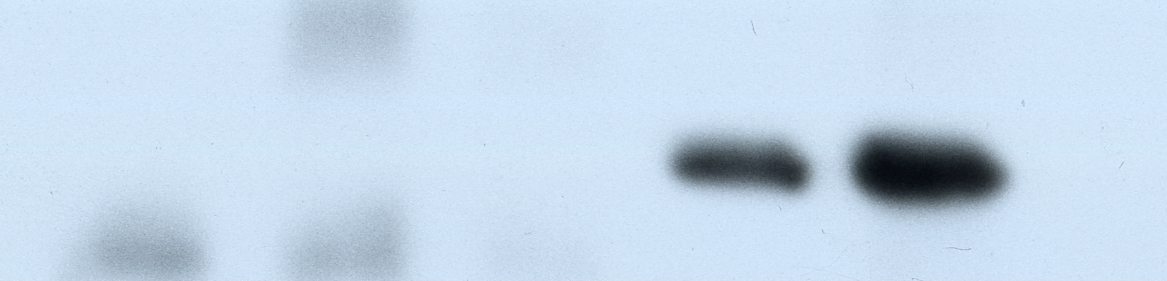

Supplement: Supplementary file 5 [file DataSheet_5.zip › fig 5/5E/fig5E A151R 1-4 lane.jpg]

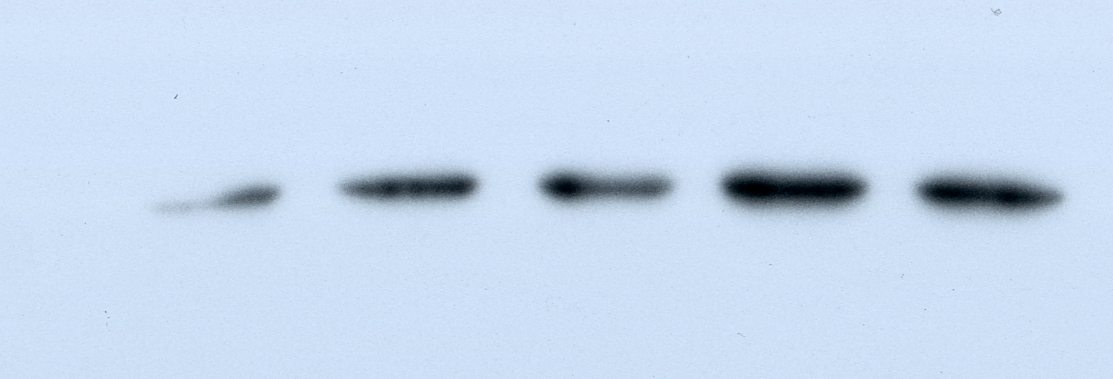

Supplement: Supplementary file 5 [file DataSheet_5.zip › fig 5/5E/fig5E Input-actin 1-4 lane.jpg]

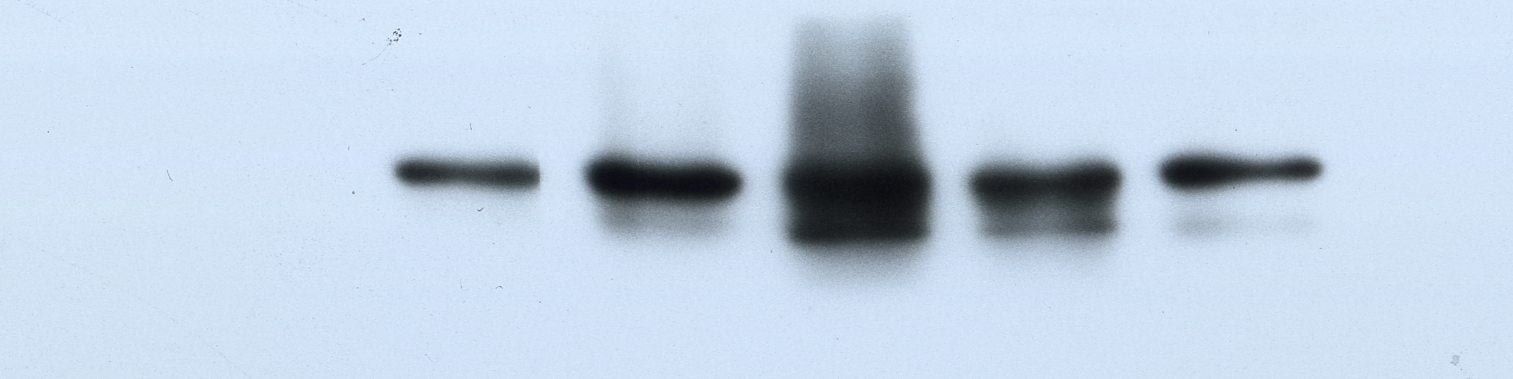

Supplement: Supplementary file 5 [file DataSheet_5.zip › fig 5/5E/fig5E Input-Flag-TBK1 1-4 lane.jpg]

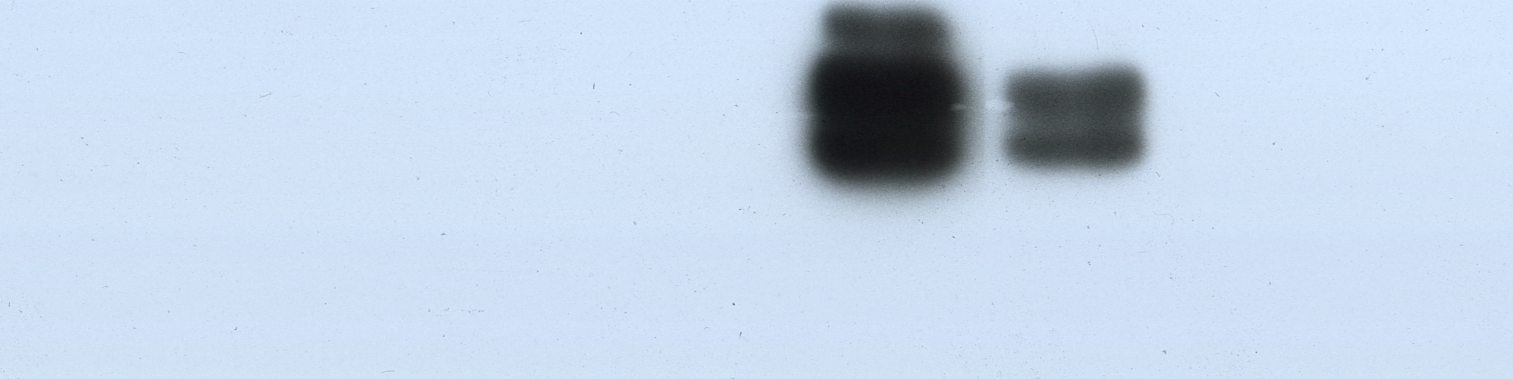

Supplement: Supplementary file 5 [file DataSheet_5.zip › fig 5/5E/fig5E Input-HA-TRAF6 1-4 lane.jpg]

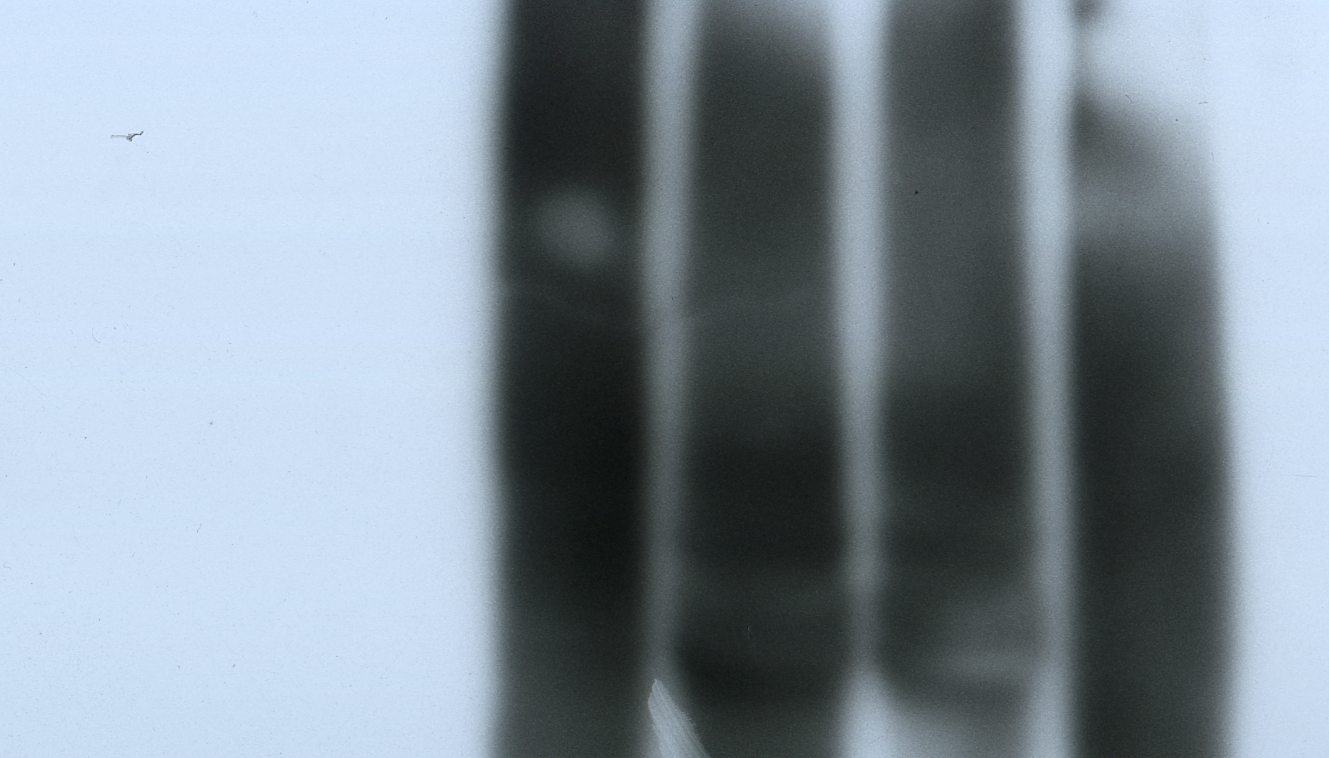

Supplement: Supplementary file 5 [file DataSheet_5.zip › fig 5/5E/fig5E Input-K63 1-4 lane (1).jpg]

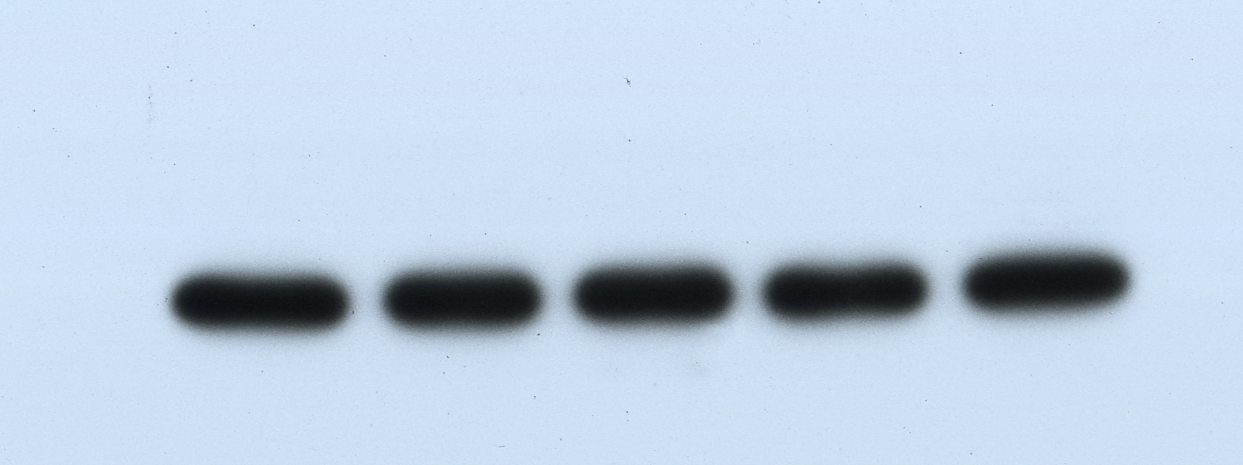

Supplement: Supplementary file 5 [file DataSheet_5.zip › fig 5/5E/fig5E IP-Flag-TBK1 1-4 lane.jpg]

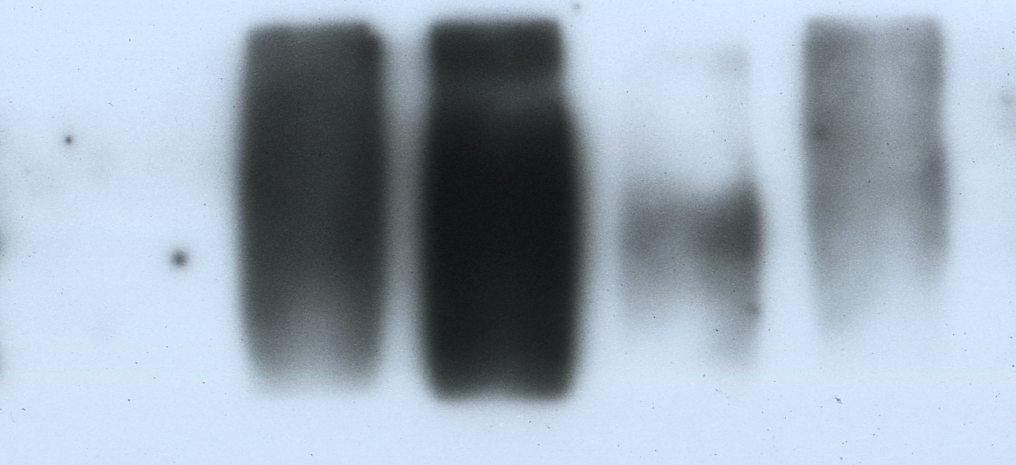

Supplement: Supplementary file 5 [file DataSheet_5.zip › fig 5/5E/fig5E IP-HA-TBK1(K63) 1-4 lane.jpg]

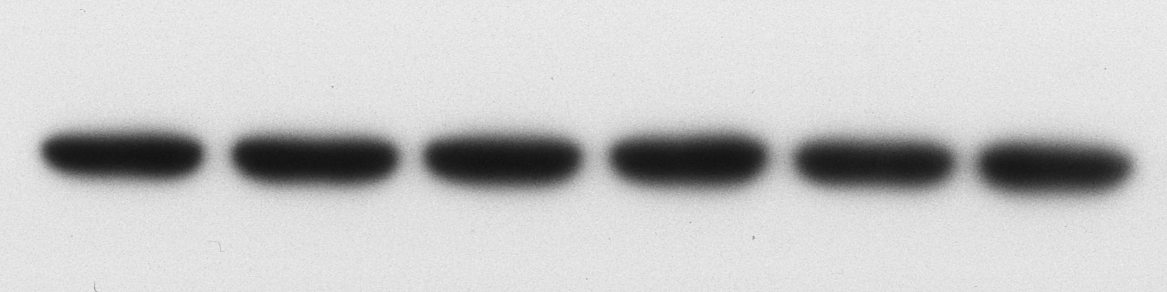

Supplement: Supplementary file 5 [file DataSheet_5.zip › fig 5/5F/fig5F B-actin.jpg]

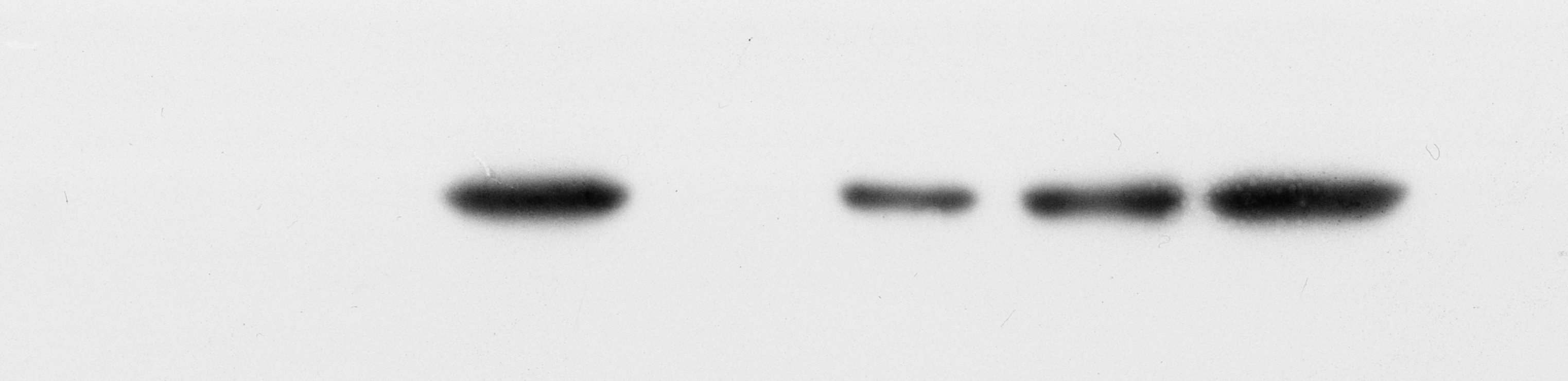

Supplement: Supplementary file 5 [file DataSheet_5.zip › fig 5/5F/fig5F HA.jpg]

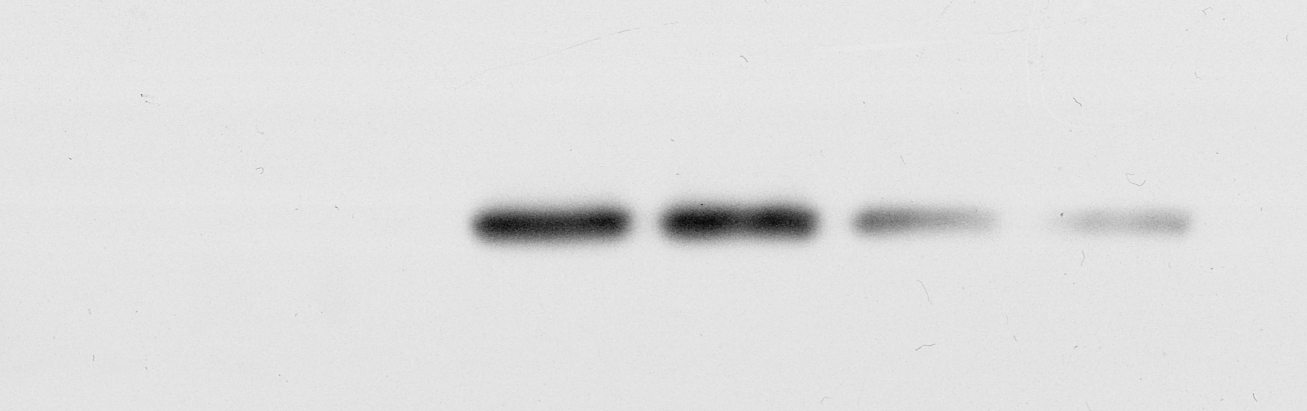

Supplement: Supplementary file 5 [file DataSheet_5.zip › fig 5/5F/fig5F p-TBK1.jpg]

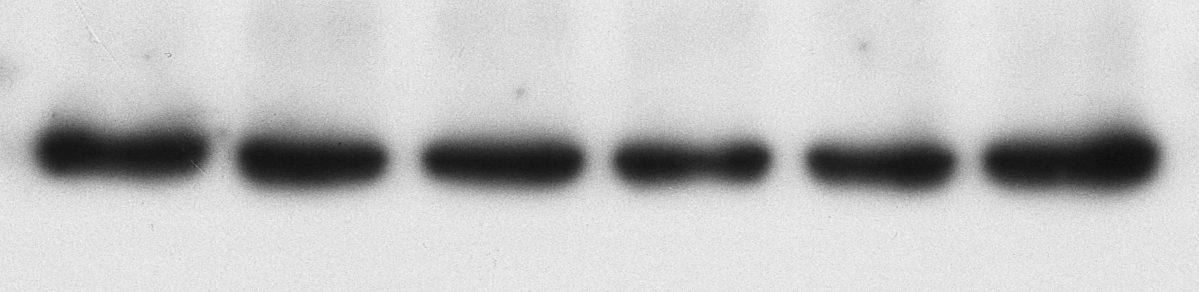

Supplement: Supplementary file 5 [file DataSheet_5.zip › fig 5/5F/fig5F TBK1.jpg]

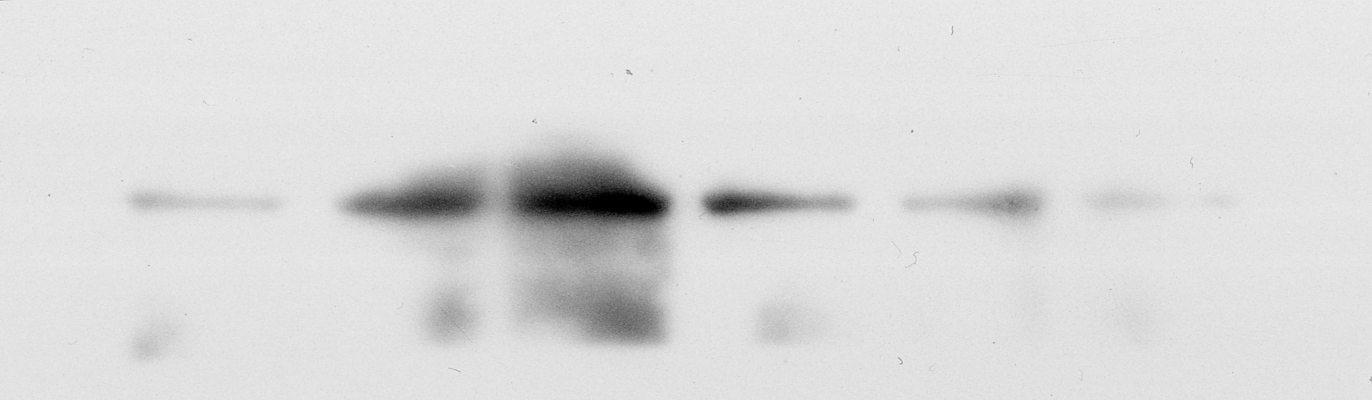

Supplement: Supplementary file 5 [file DataSheet_5.zip › fig 5/5F/fig5F TRAF6.jpg]

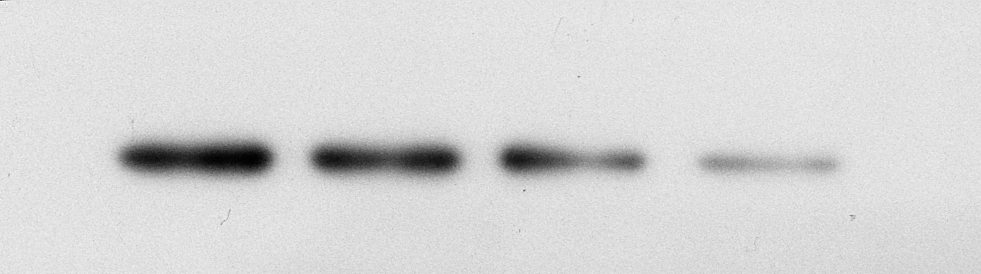

Supplement: Supplementary file 6 [file DataSheet_6.zip › fig 6/6A/FIG6A Flag-TRAF6.jpg]

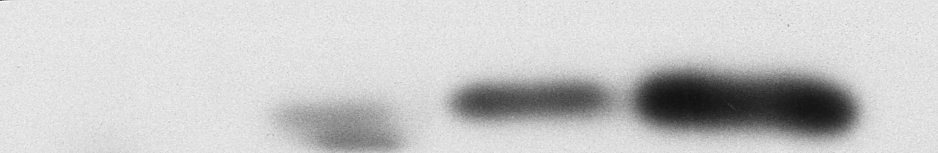

Supplement: Supplementary file 6 [file DataSheet_6.zip › fig 6/6A/FIG6A HA-A151R005.jpg]

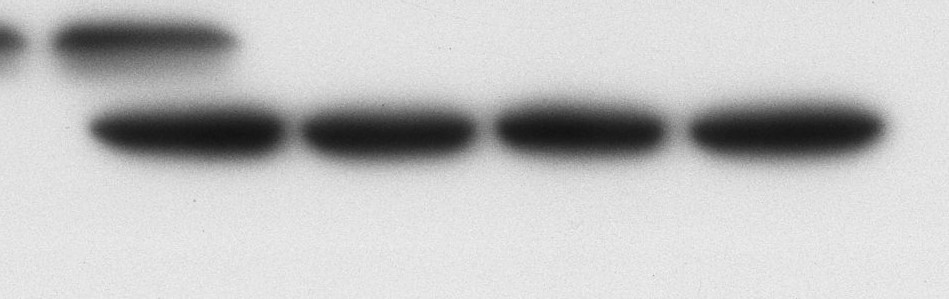

Supplement: Supplementary file 6 [file DataSheet_6.zip › fig 6/6A/FIG6A a┬-actin.jpg]

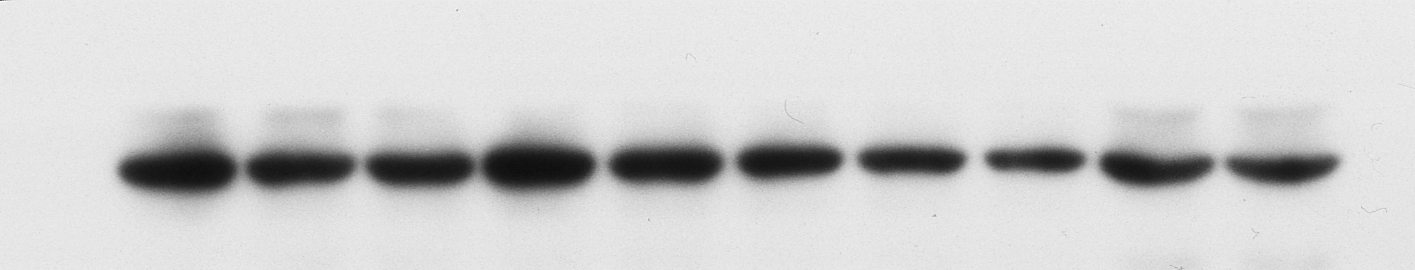

Supplement: Supplementary file 6 [file DataSheet_6.zip › fig 6/6D/FIG6D ACTIN.jpg]

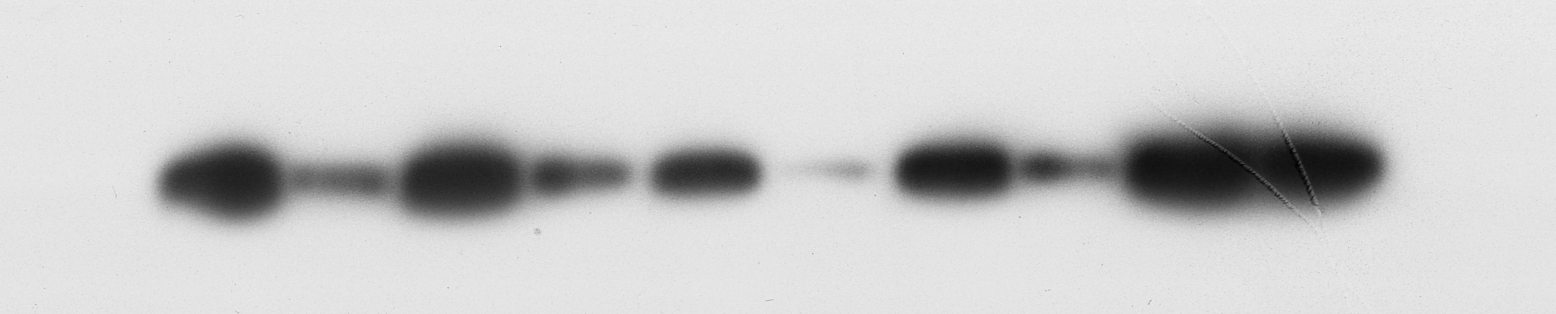

Supplement: Supplementary file 6 [file DataSheet_6.zip › fig 6/6D/FIG6D Flag-TRAF6 (2).jpg]

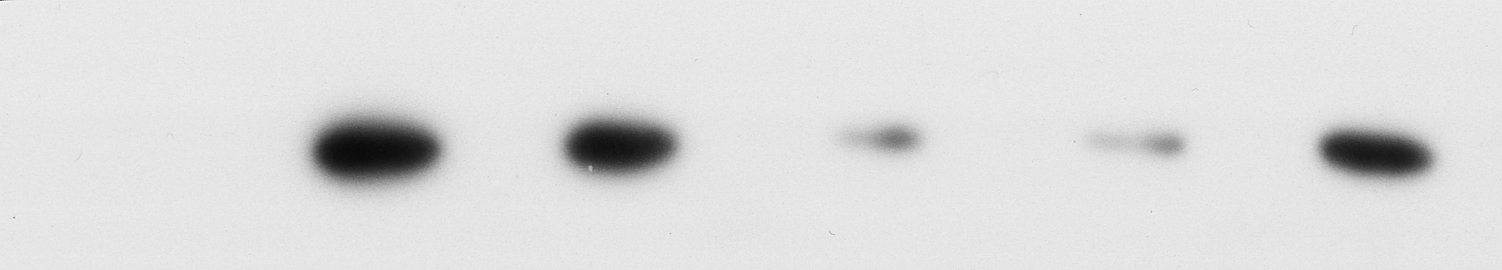

Supplement: Supplementary file 6 [file DataSheet_6.zip › fig 6/6D/FIG6D HA-A151R.jpg]

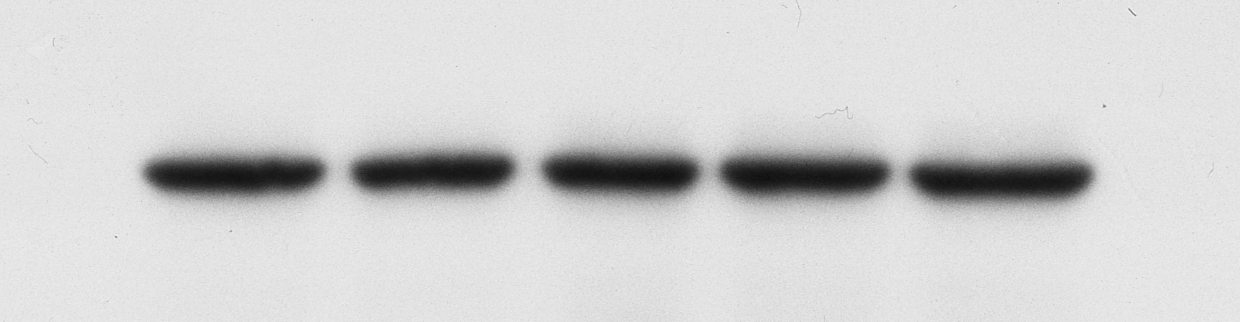

Supplement: Supplementary file 6 [file DataSheet_6.zip › fig 6/6E/FIG6E actin.jpg]

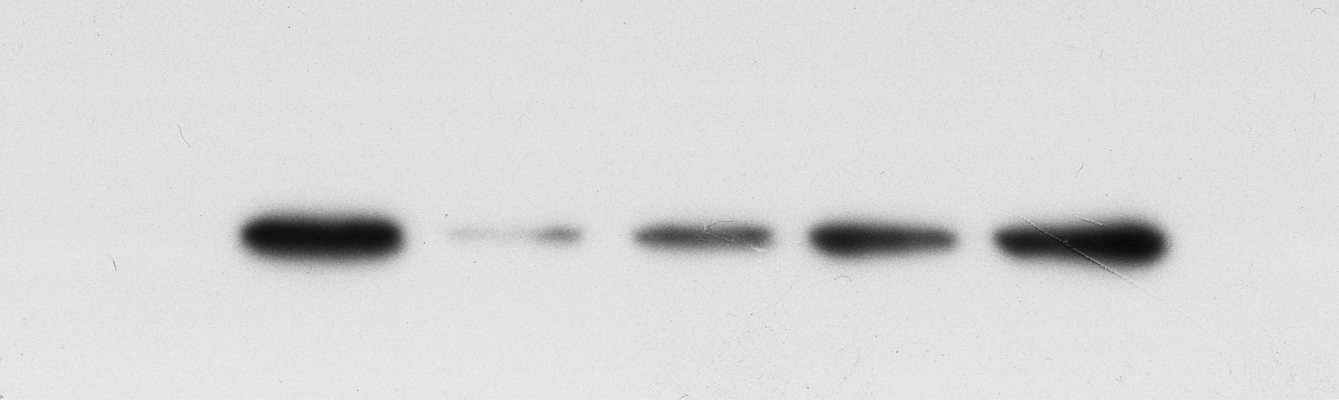

Supplement: Supplementary file 6 [file DataSheet_6.zip › fig 6/6E/FIG6E Flag-TRAF6.jpg]

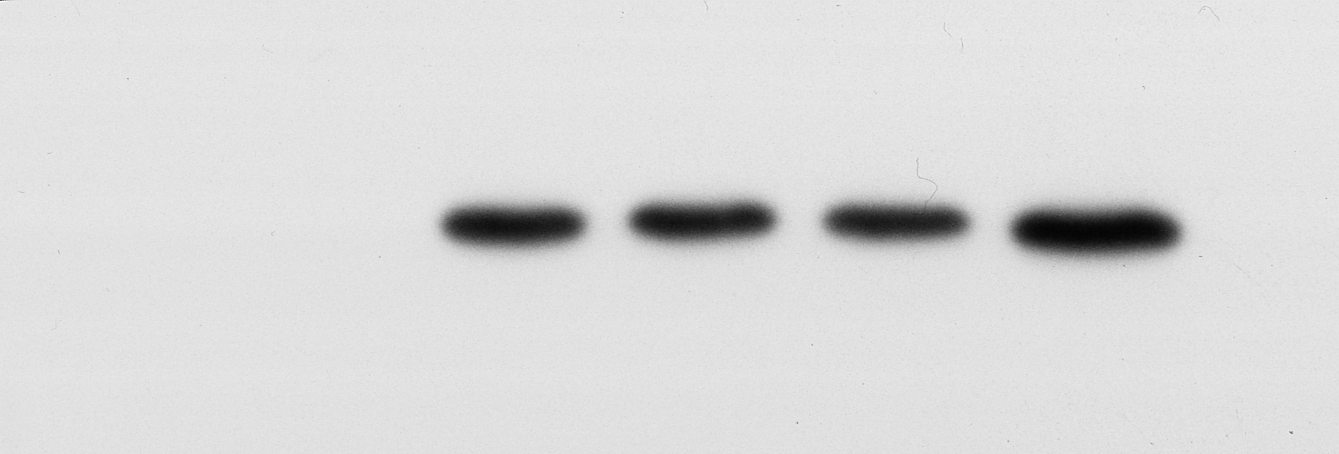

Supplement: Supplementary file 6 [file DataSheet_6.zip › fig 6/6E/FIG6E HA-A151R.jpg]

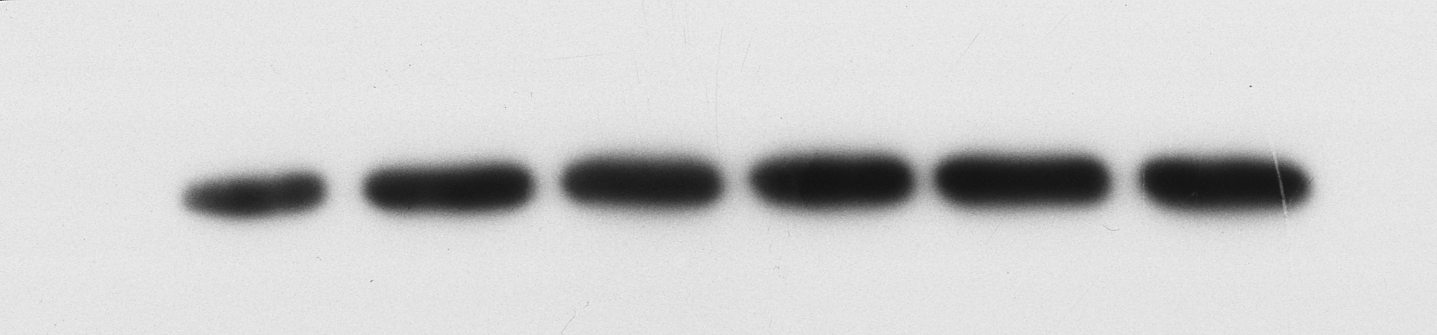

Supplement: Supplementary file 6 [file DataSheet_6.zip › fig 6/6F/FIG6F ACTIN.jpg]

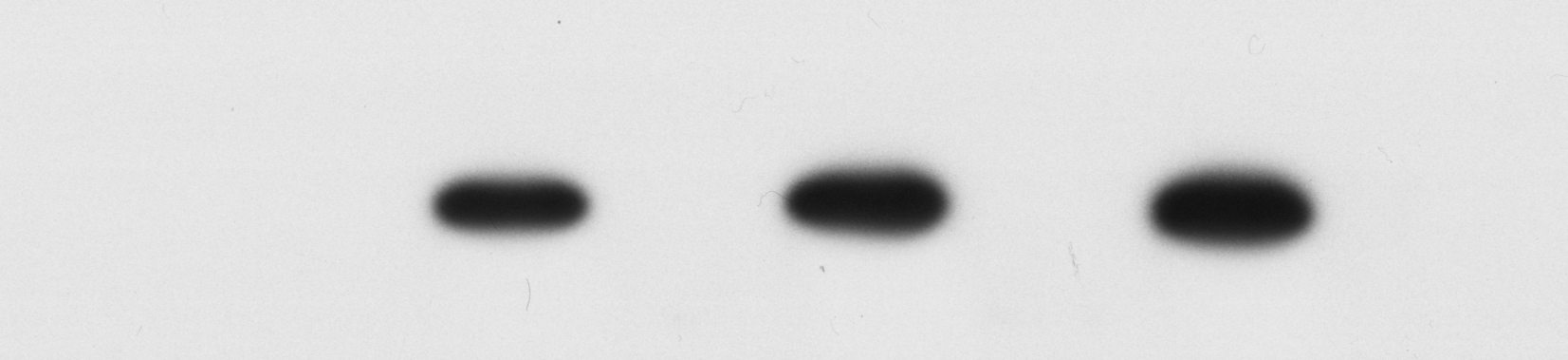

Supplement: Supplementary file 6 [file DataSheet_6.zip › fig 6/6F/FIG6F HA-A151R.jpg]

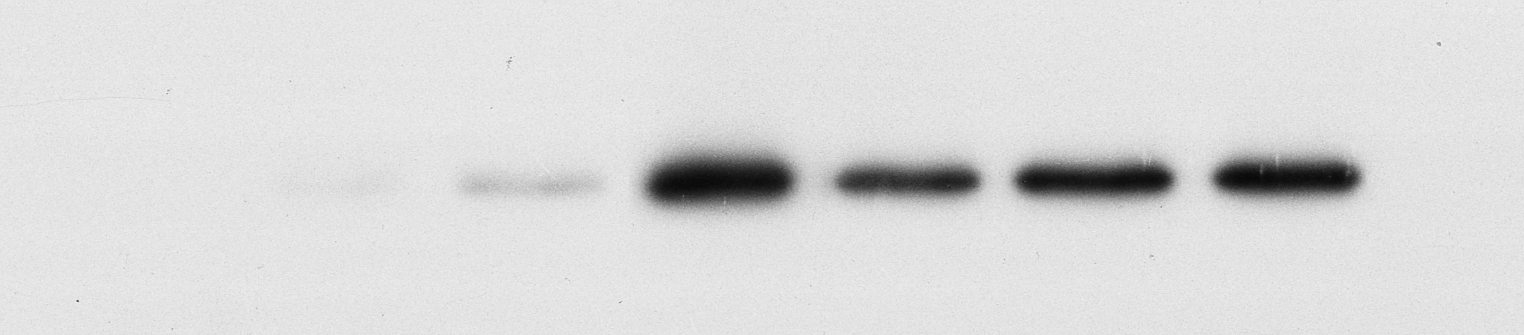

Supplement: Supplementary file 6 [file DataSheet_6.zip › fig 6/6F/FIG6F p-TBK1.jpg]

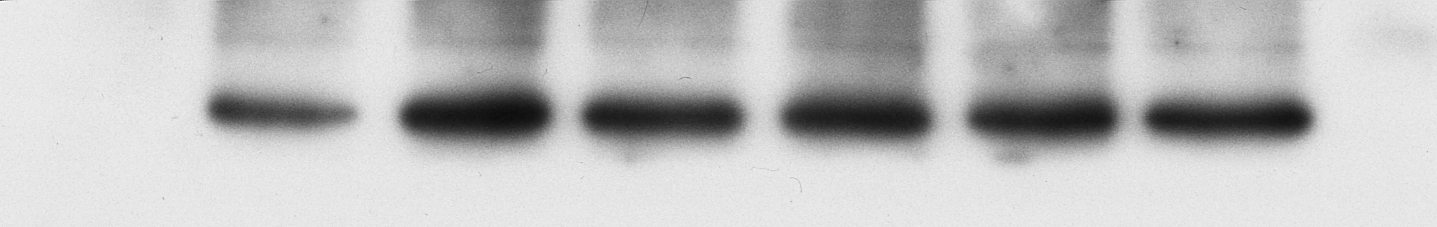

Supplement: Supplementary file 6 [file DataSheet_6.zip › fig 6/6F/FIG6F TBK1.jpg]

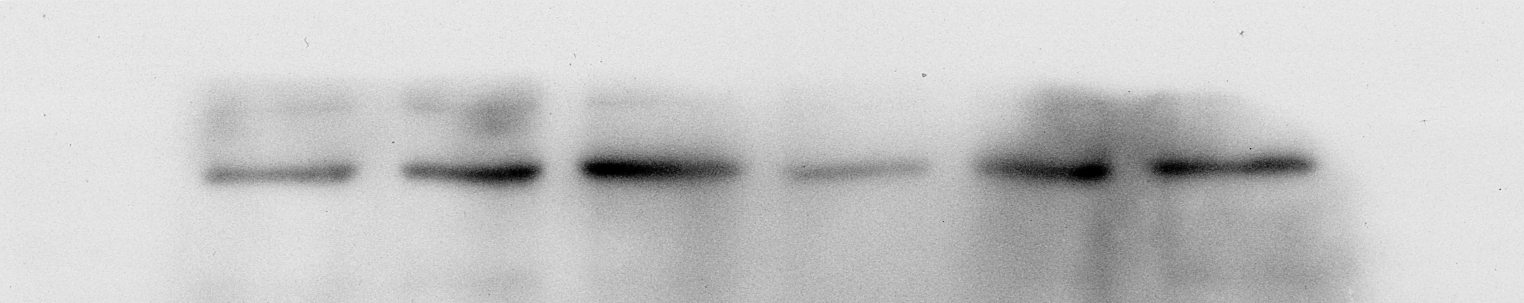

Supplement: Supplementary file 6 [file DataSheet_6.zip › fig 6/6F/FIG6F TRAF6.jpg]

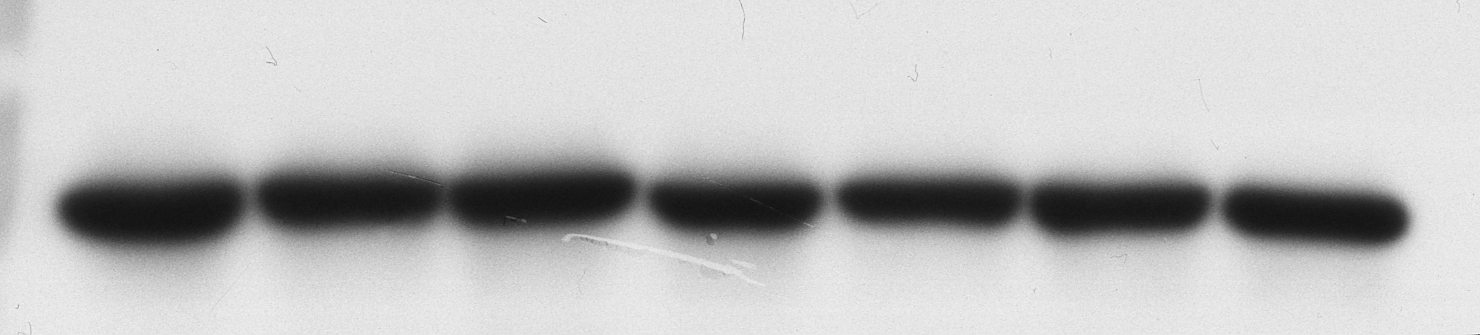

Supplement: Supplementary file 7 [file DataSheet_7.zip › fig 7/7D/fig7D actin.jpg]

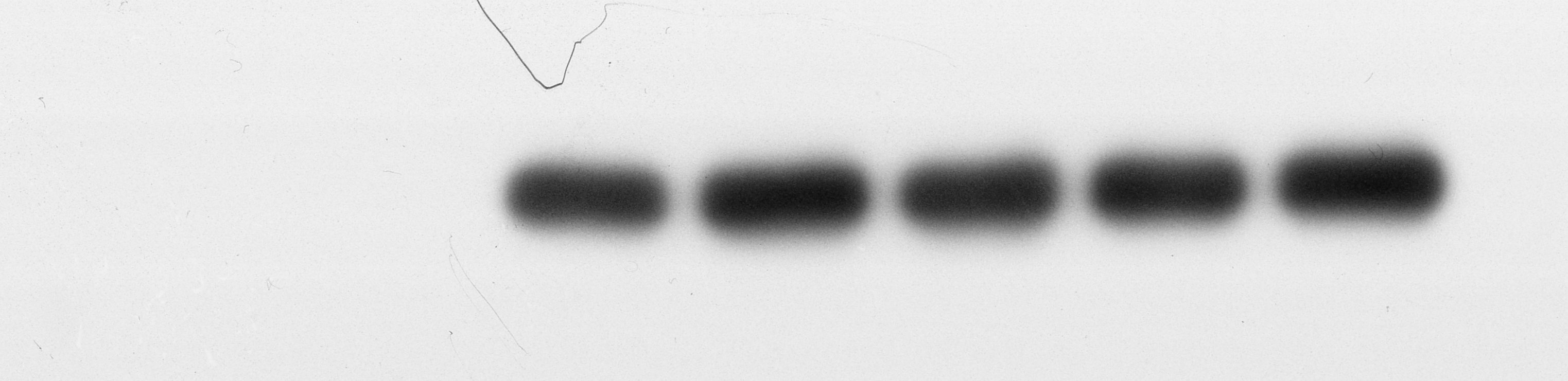

Supplement: Supplementary file 7 [file DataSheet_7.zip › fig 7/7D/fig7D anti HA.jpg]

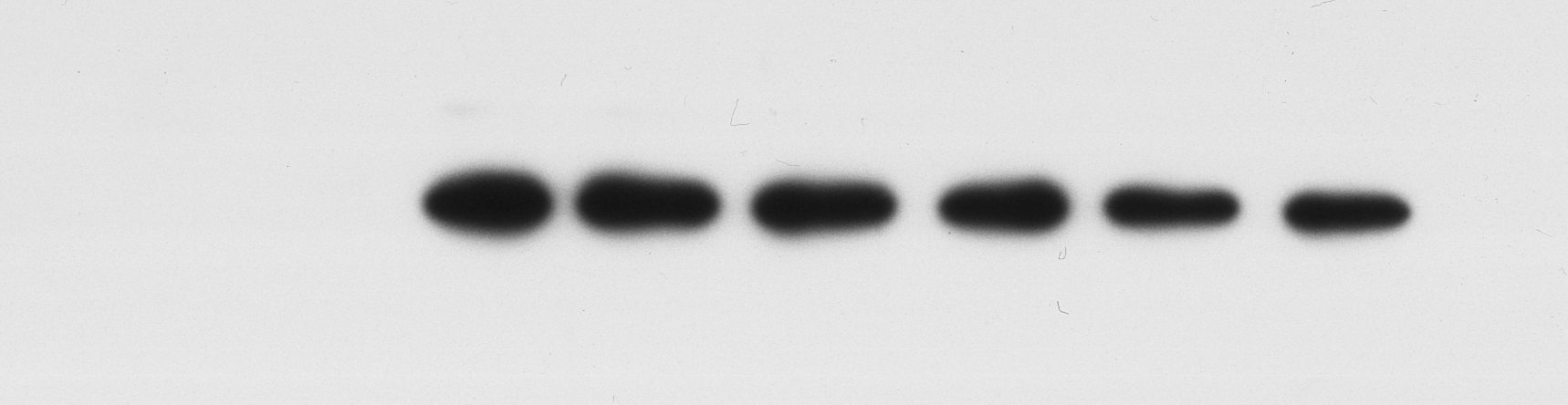

Supplement: Supplementary file 7 [file DataSheet_7.zip › fig 7/7D/fig7D F-cgas.jpg]

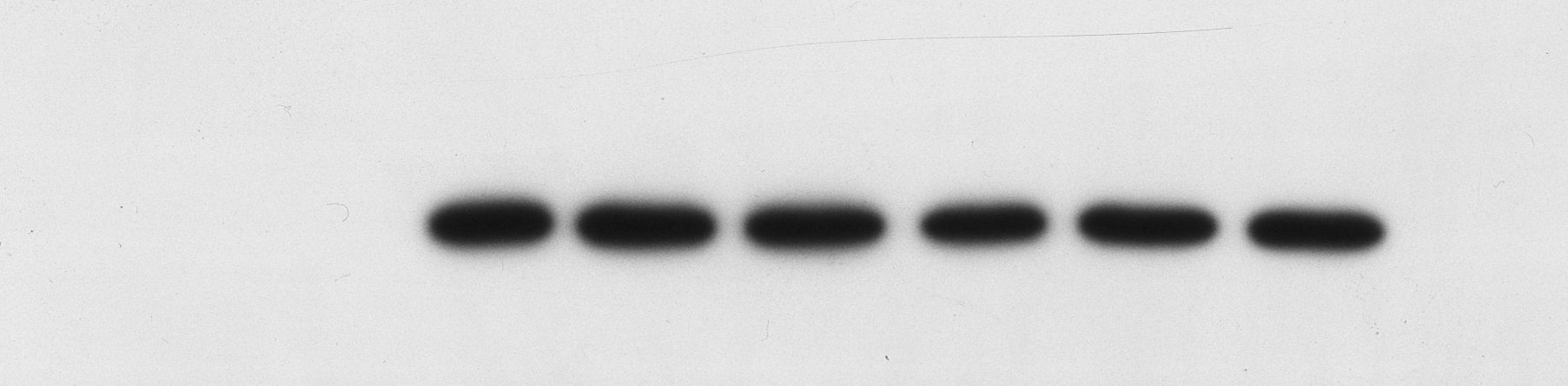

Supplement: Supplementary file 7 [file DataSheet_7.zip › fig 7/7D/fig7D F-STING.jpg]

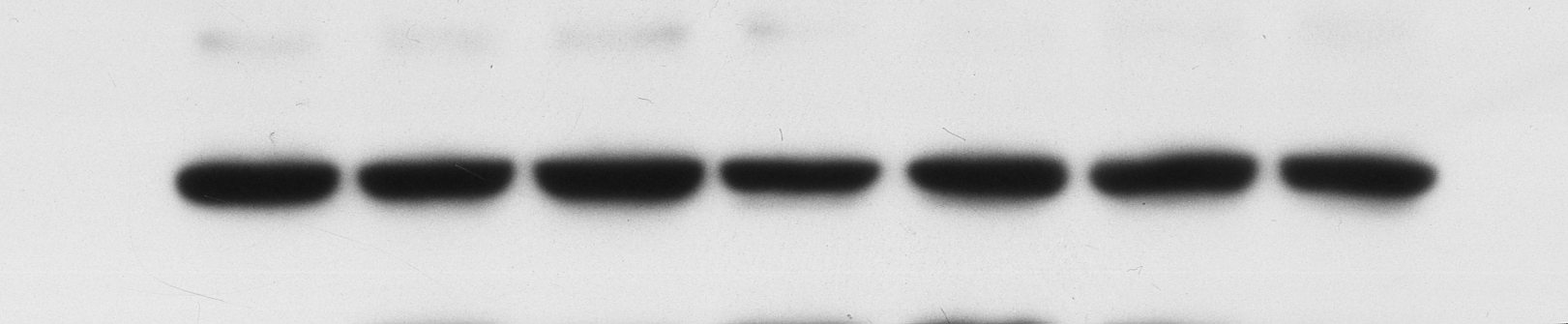

Supplement: Supplementary file 7 [file DataSheet_7.zip › fig 7/7D/fig7D IRF3.jpg]

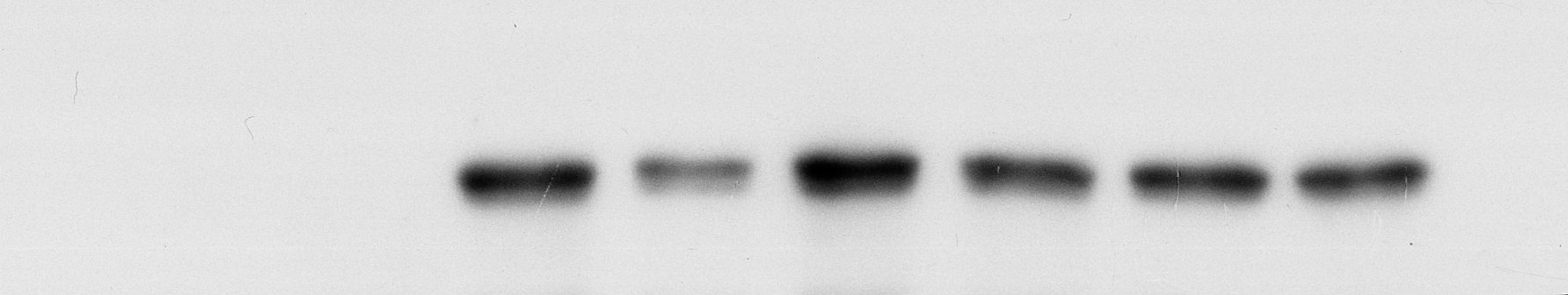

Supplement: Supplementary file 7 [file DataSheet_7.zip › fig 7/7D/fig7D p-IRF3.jpg]

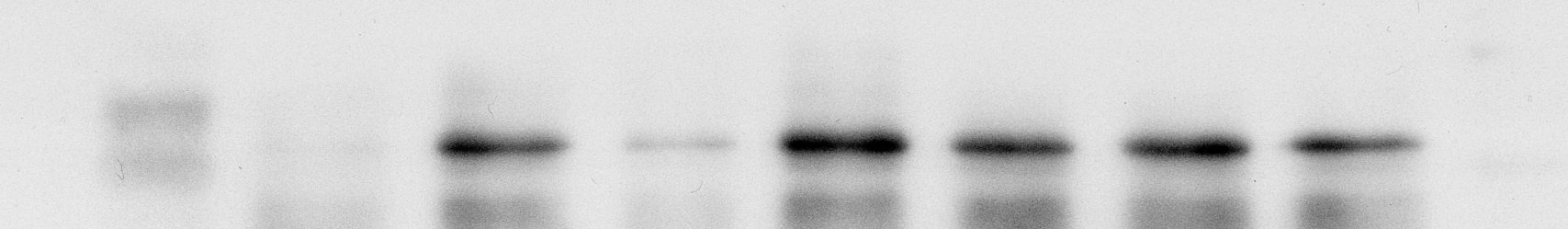

Supplement: Supplementary file 7 [file DataSheet_7.zip › fig 7/7D/fig7D p-P65.jpg]

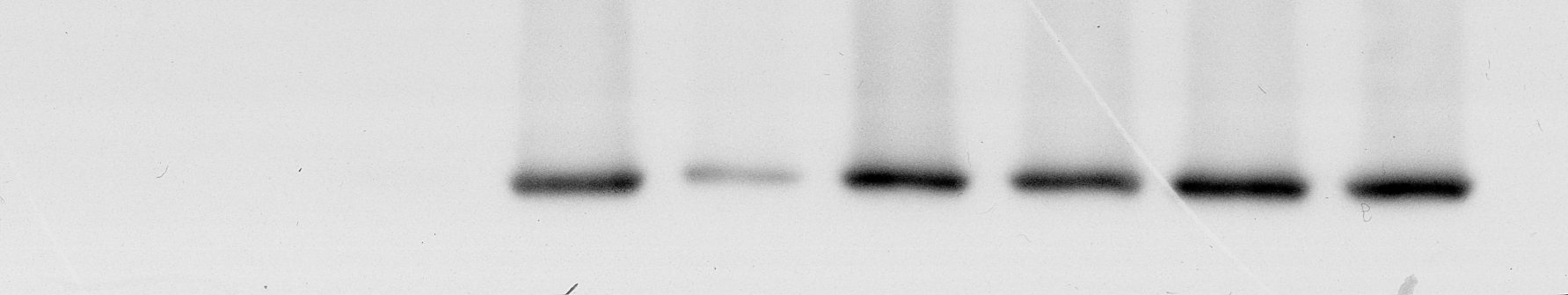

Supplement: Supplementary file 7 [file DataSheet_7.zip › fig 7/7D/fig7D p-TBK1.jpg]

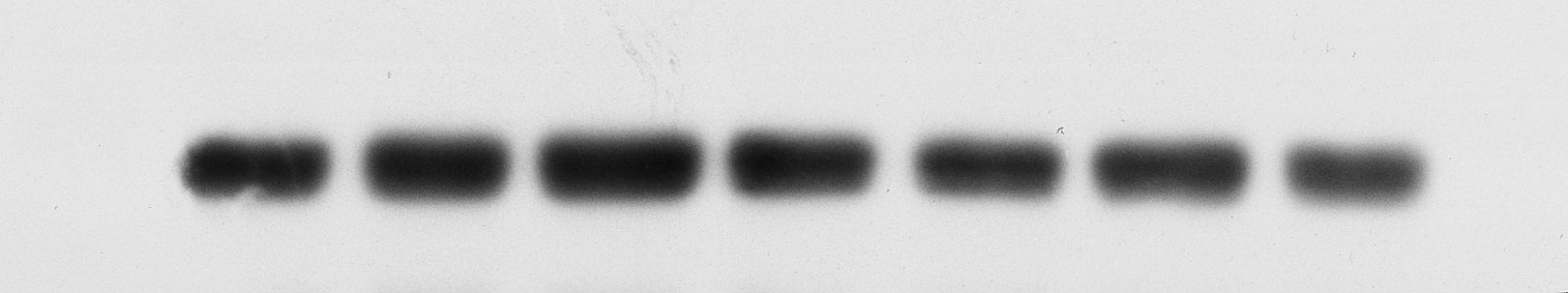

Supplement: Supplementary file 7 [file DataSheet_7.zip › fig 7/7D/fig7D P65.jpg]

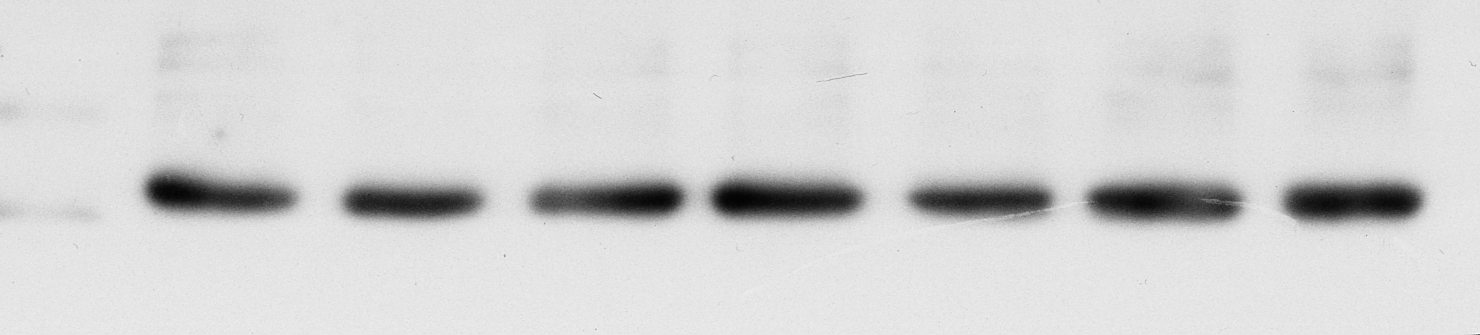

Supplement: Supplementary file 7 [file DataSheet_7.zip › fig 7/7D/fig7D TBK1.jpg]

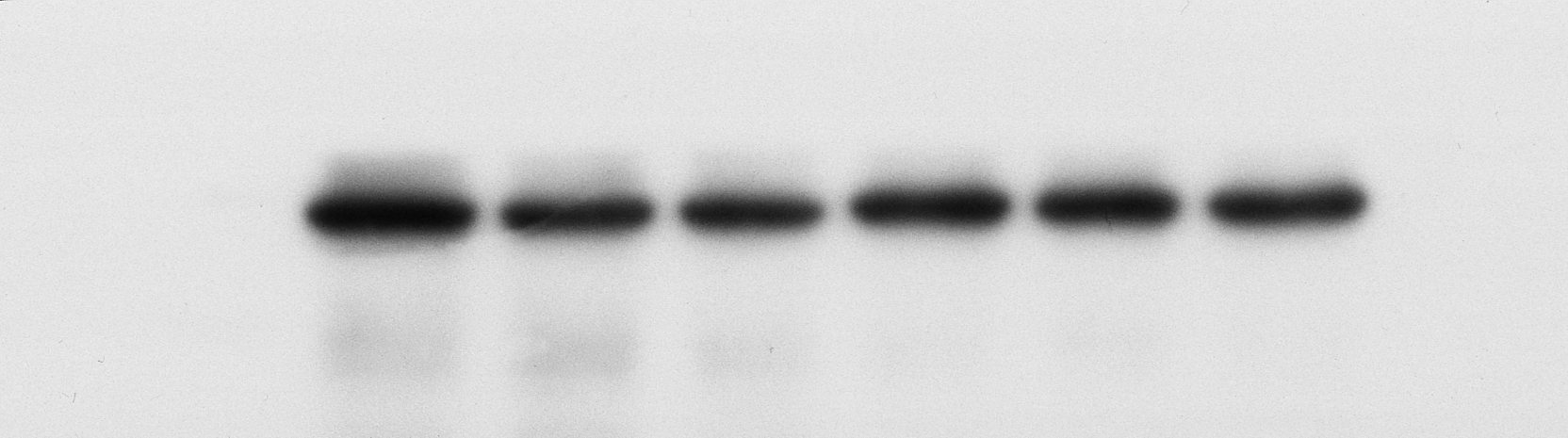

Supplement: Supplementary file 7 [file DataSheet_7.zip › fig 7/7F/fig7F INPUT-ACTIN.jpg]

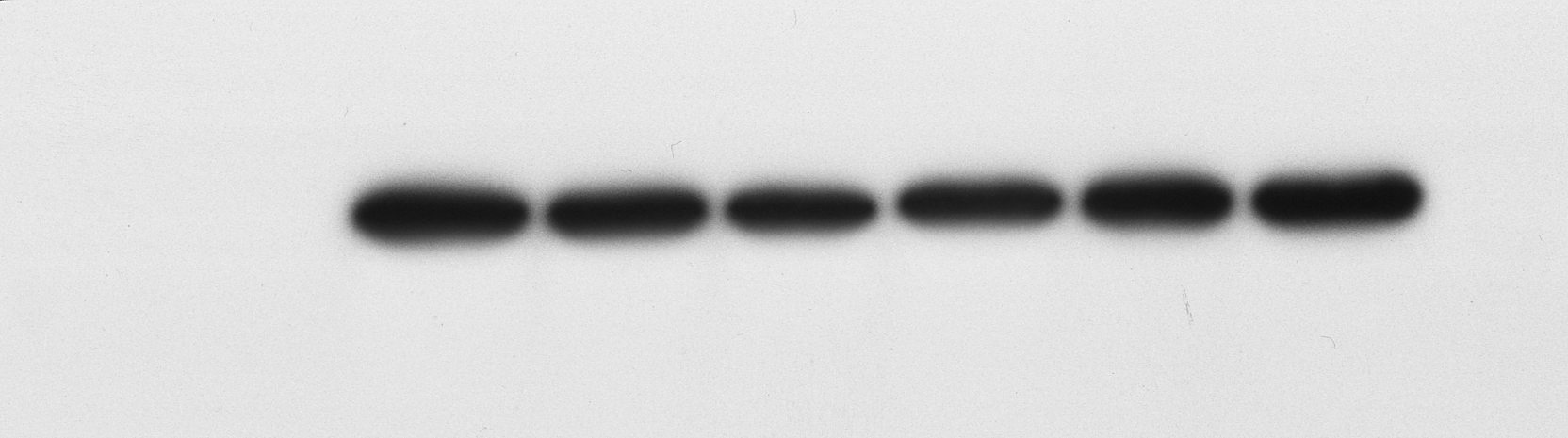

Supplement: Supplementary file 7 [file DataSheet_7.zip › fig 7/7F/fig7F INPUT-FLAG-TRAF6.jpg]

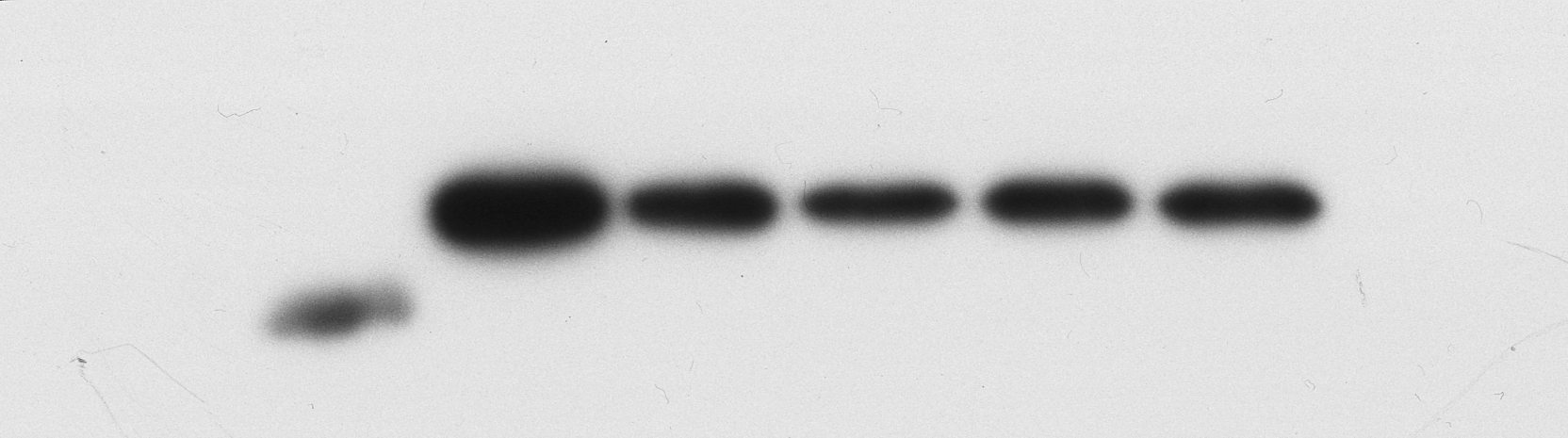

Supplement: Supplementary file 7 [file DataSheet_7.zip › fig 7/7F/fig7F INPUT-HA-A151R.jpg]

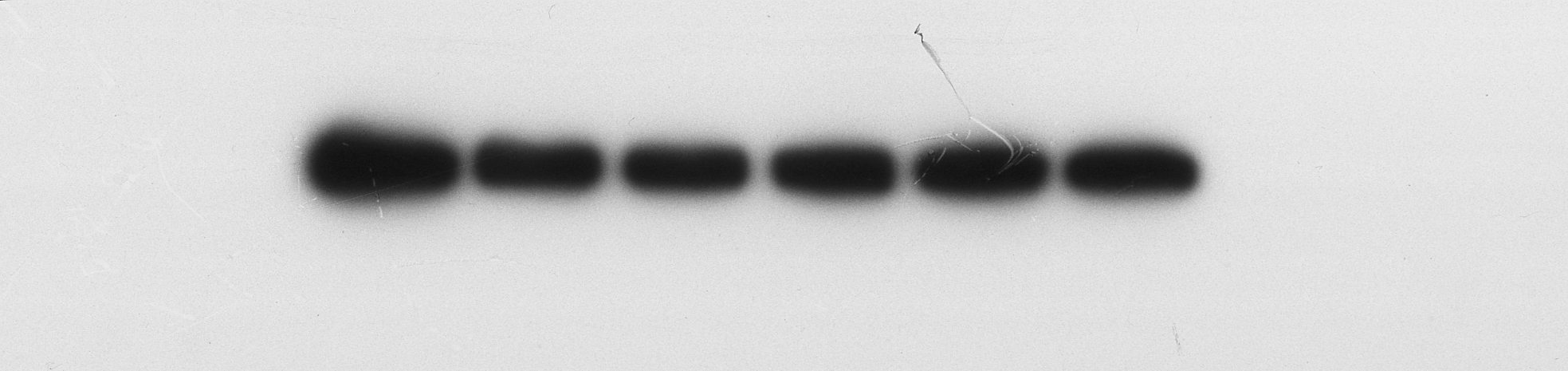

Supplement: Supplementary file 7 [file DataSheet_7.zip › fig 7/7F/fig7F IP-Flag-TRAF6.jpg]

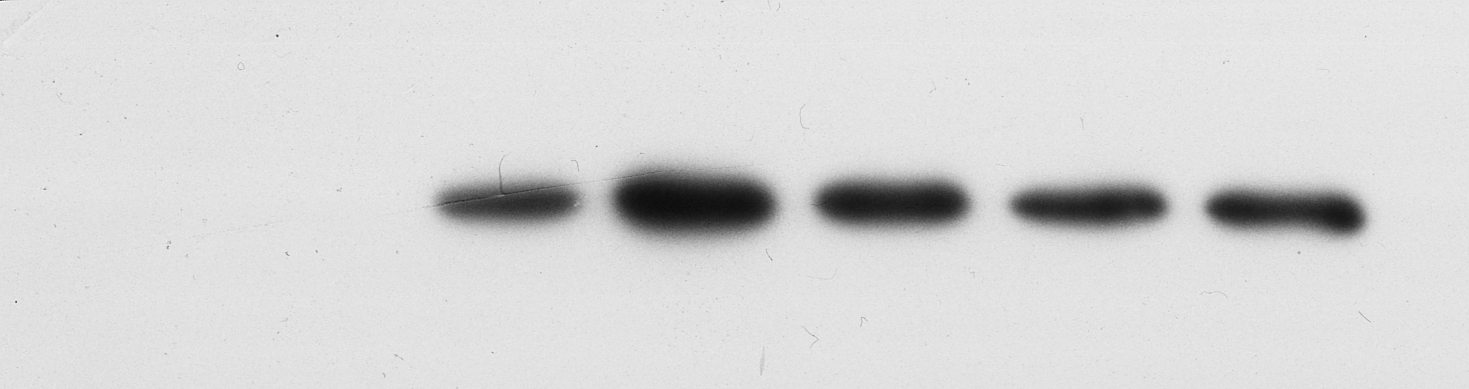

Supplement: Supplementary file 7 [file DataSheet_7.zip › fig 7/7F/fig7F ip-HA-MA151R.jpg]

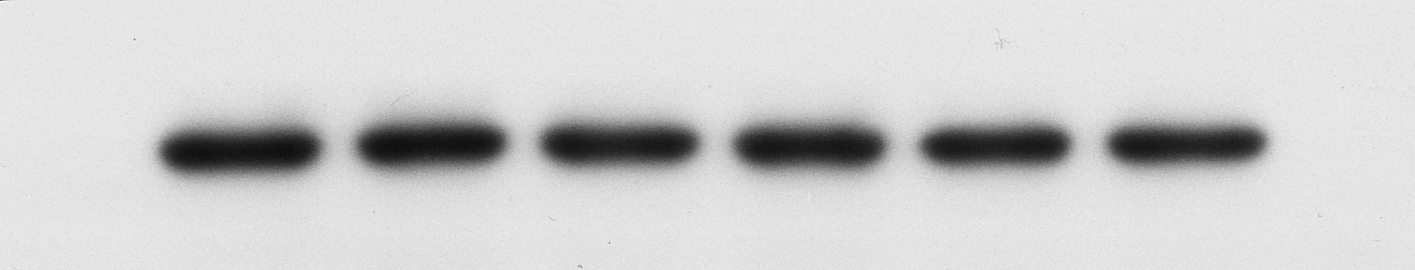

Supplement: Supplementary file 7 [file DataSheet_7.zip › fig 7/7G/fig7G ACTIN.jpg]

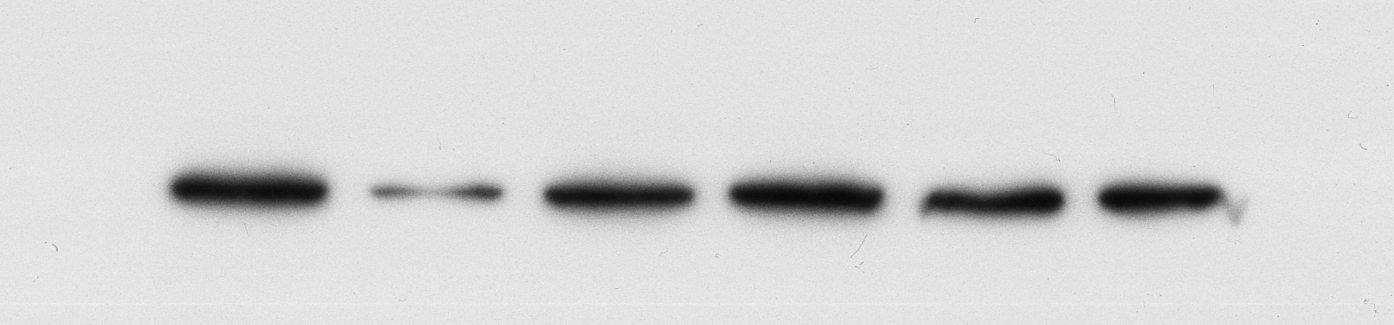

Supplement: Supplementary file 7 [file DataSheet_7.zip › fig 7/7G/fig7G Flag-TRAF6.jpg]

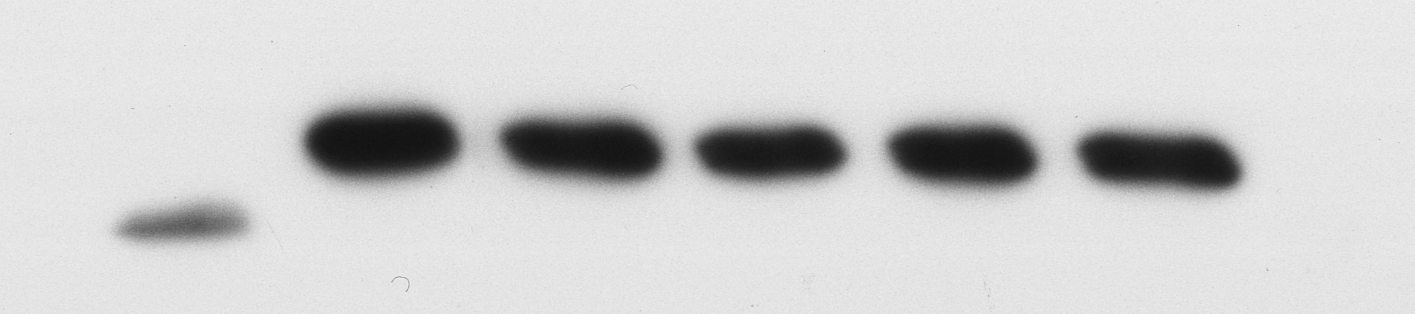

Supplement: Supplementary file 7 [file DataSheet_7.zip › fig 7/7G/fig7G HA-M A151R.jpg]

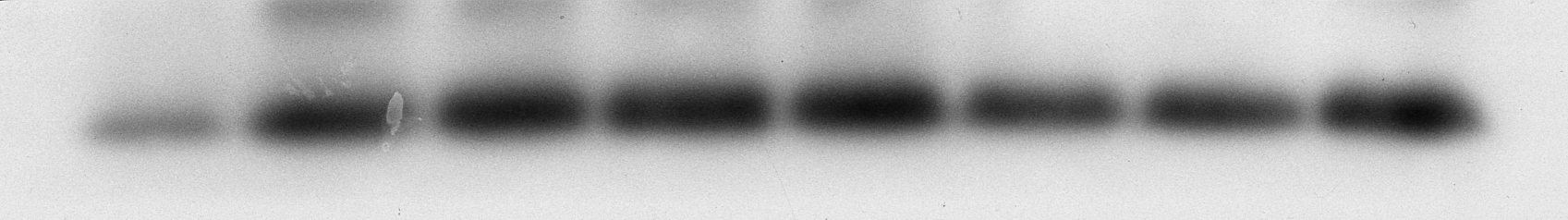

Supplement: Supplementary file 8 [file DataSheet_8.zip › fig 8/8B/fig8B actin lane2-8 for this figure.jpg]

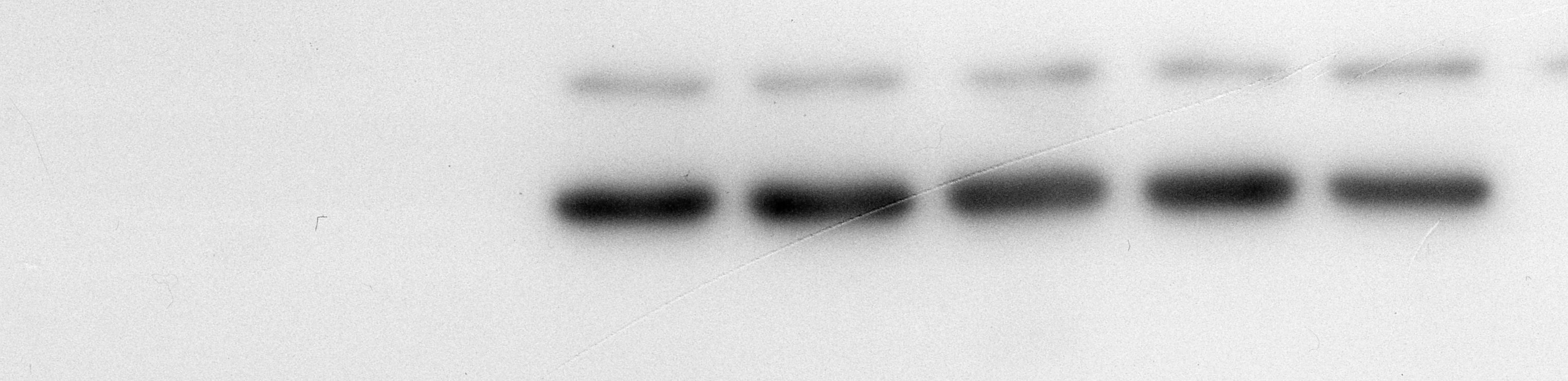

Supplement: Supplementary file 8 [file DataSheet_8.zip › fig 8/8B/fig8B anti HA wt H C C C lane1-5.jpg]

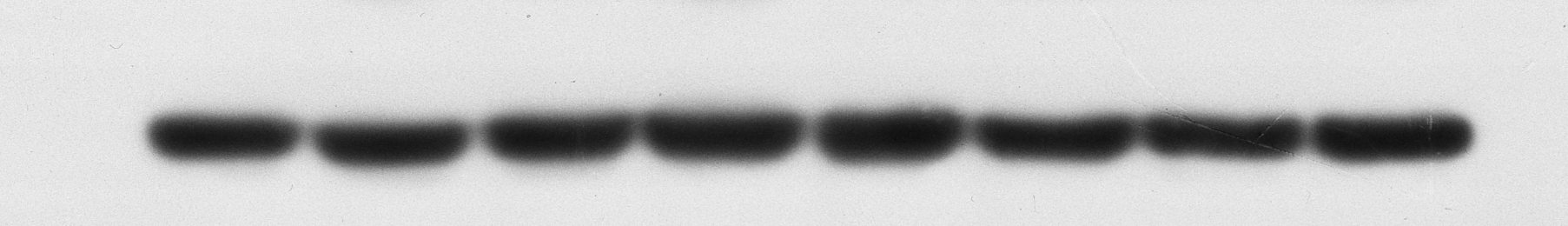

Supplement: Supplementary file 8 [file DataSheet_8.zip › fig 8/8B/fig8B IRF3 lane2-8 for this figure.jpg]

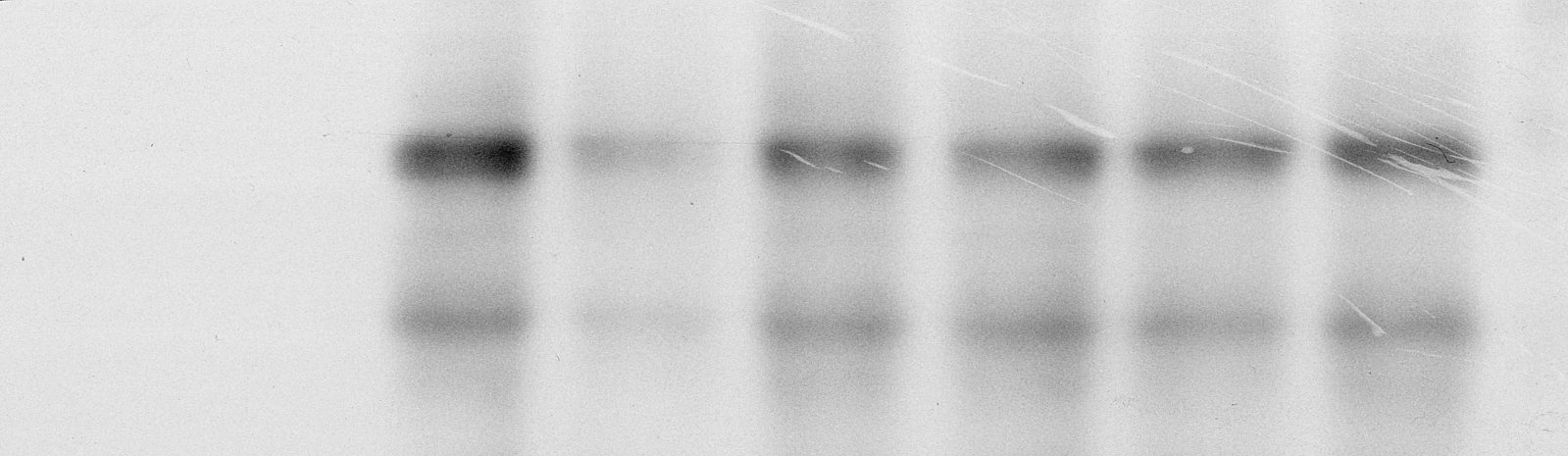

Supplement: Supplementary file 8 [file DataSheet_8.zip › fig 8/8B/fig8B p-P65 UP p-IRF3 down.jpg]

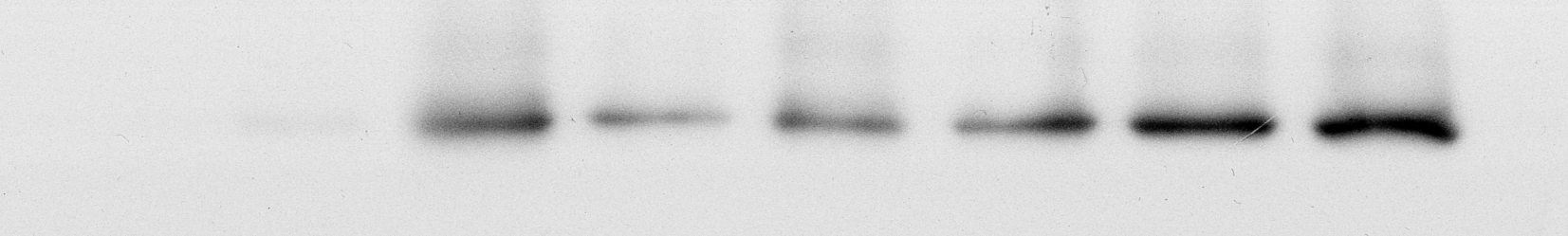

Supplement: Supplementary file 8 [file DataSheet_8.zip › fig 8/8B/fig8B p-TBK1015.jpg]

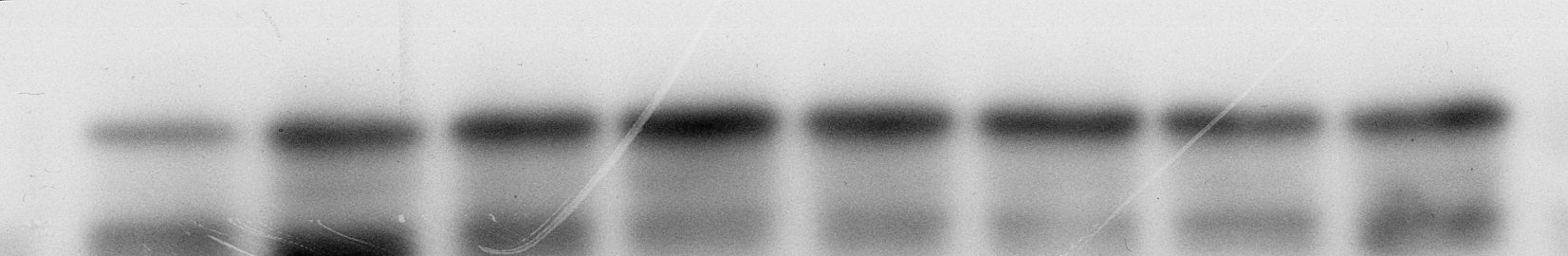

Supplement: Supplementary file 8 [file DataSheet_8.zip › fig 8/8B/fig8B P65 lane2-8 for this figure.jpg]

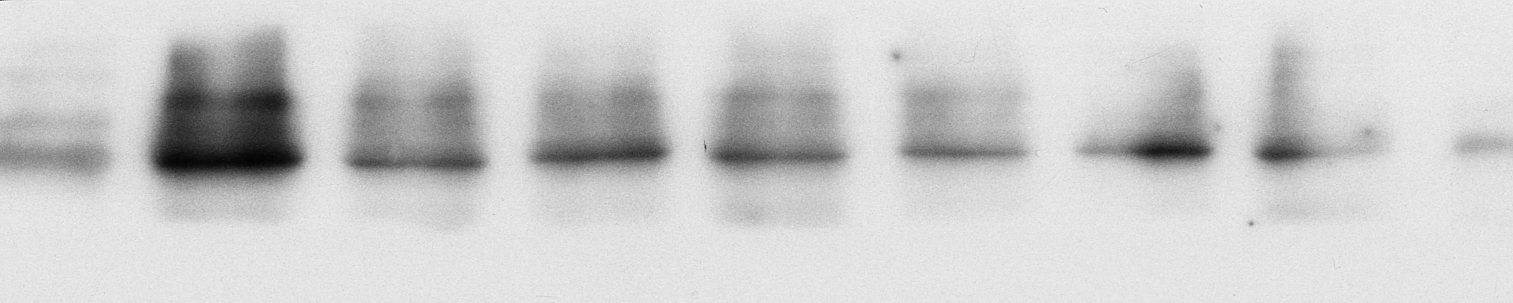

Supplement: Supplementary file 8 [file DataSheet_8.zip › fig 8/8B/fig8B TBK1 lane2-8 for this figure.jpg]

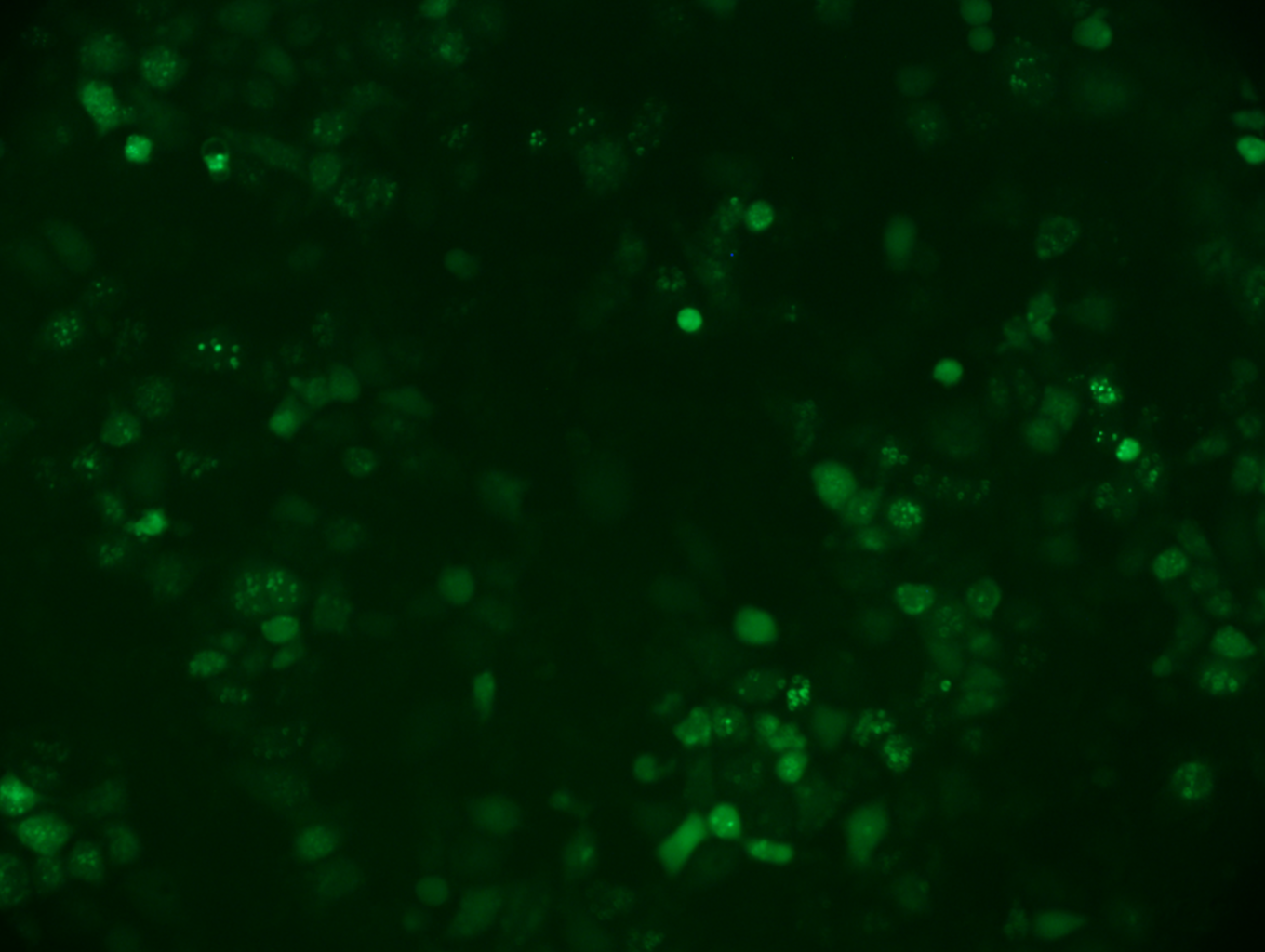

Supplement: Supplementary file 8 [file DataSheet_8.zip › fig 8/8D/HSV-1 C109 1.tif]

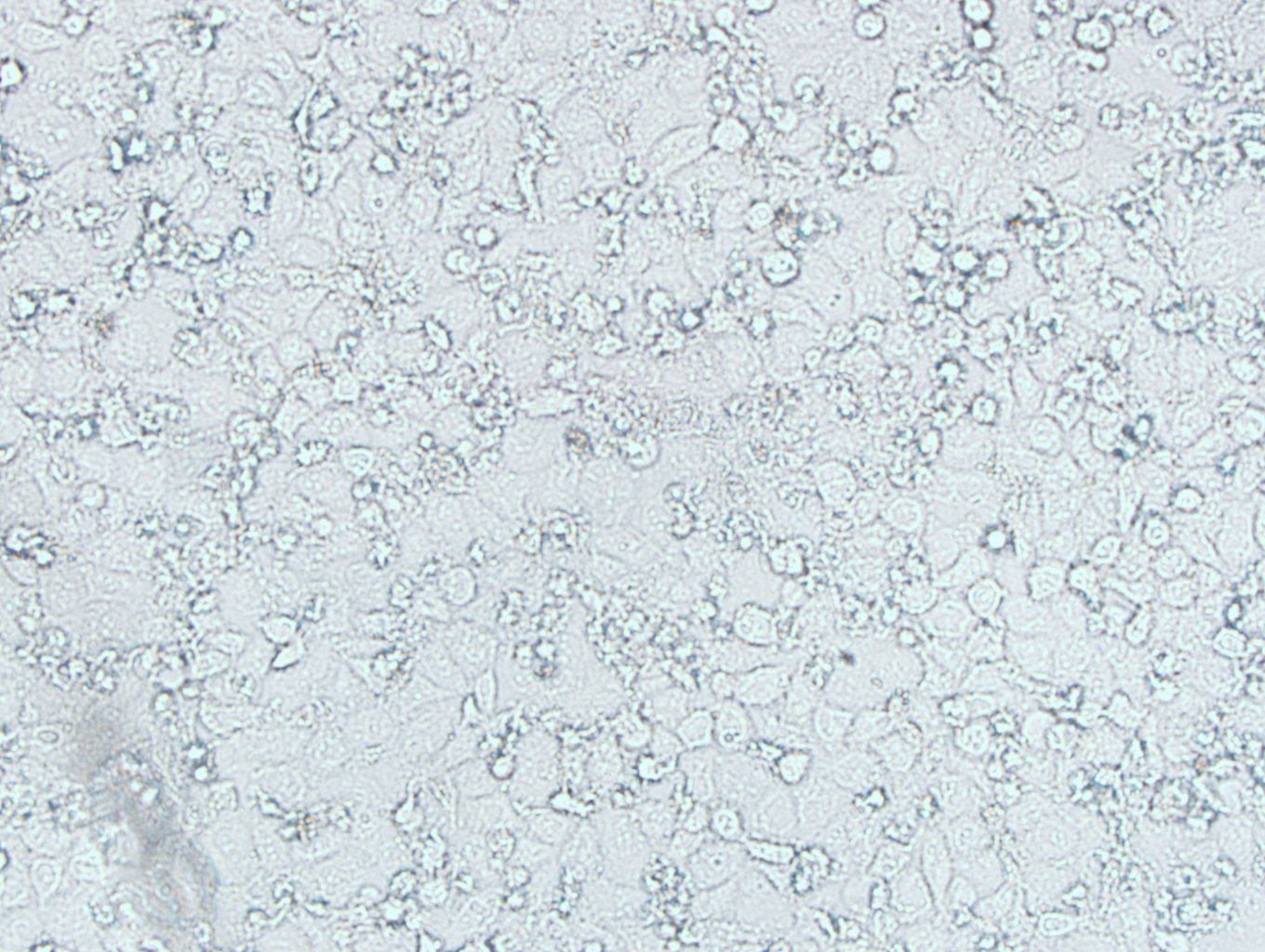

Supplement: Supplementary file 8 [file DataSheet_8.zip › fig 8/8D/HSV-1 C109 2.tif]

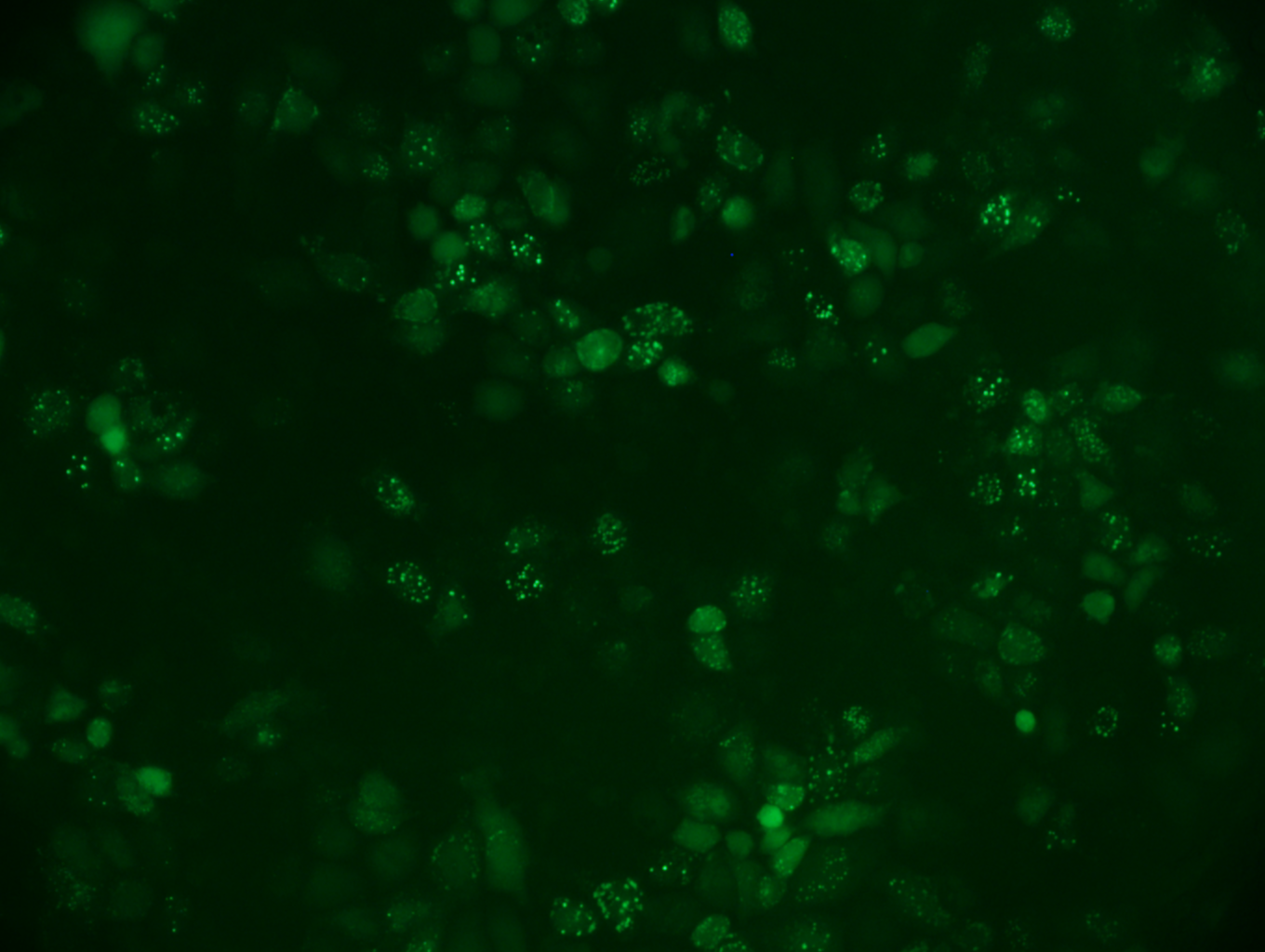

Supplement: Supplementary file 8 [file DataSheet_8.zip › fig 8/8D/HSV-1 C132 1.tif]

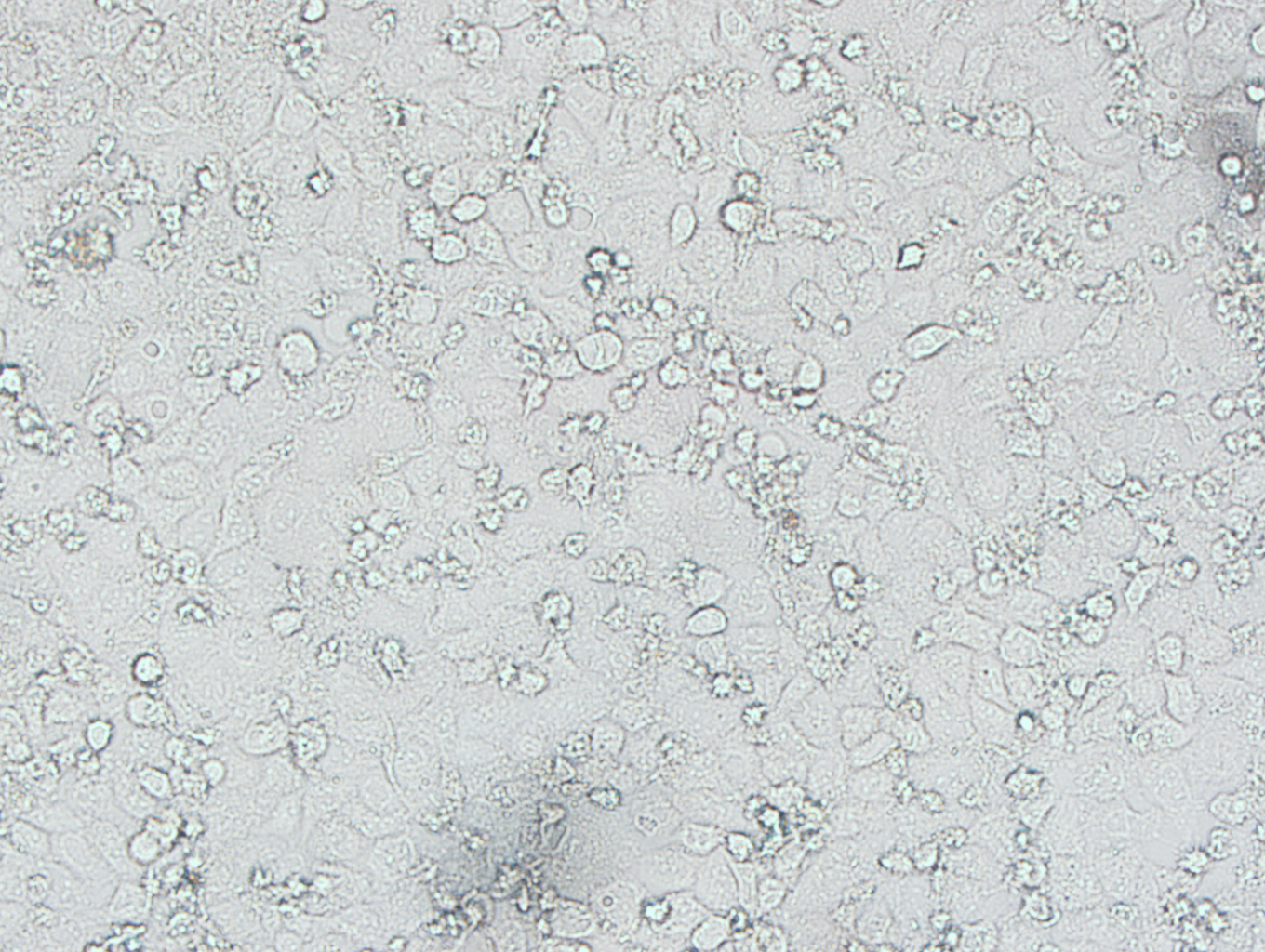

Supplement: Supplementary file 8 [file DataSheet_8.zip › fig 8/8D/HSV-1 C132 2.tif]

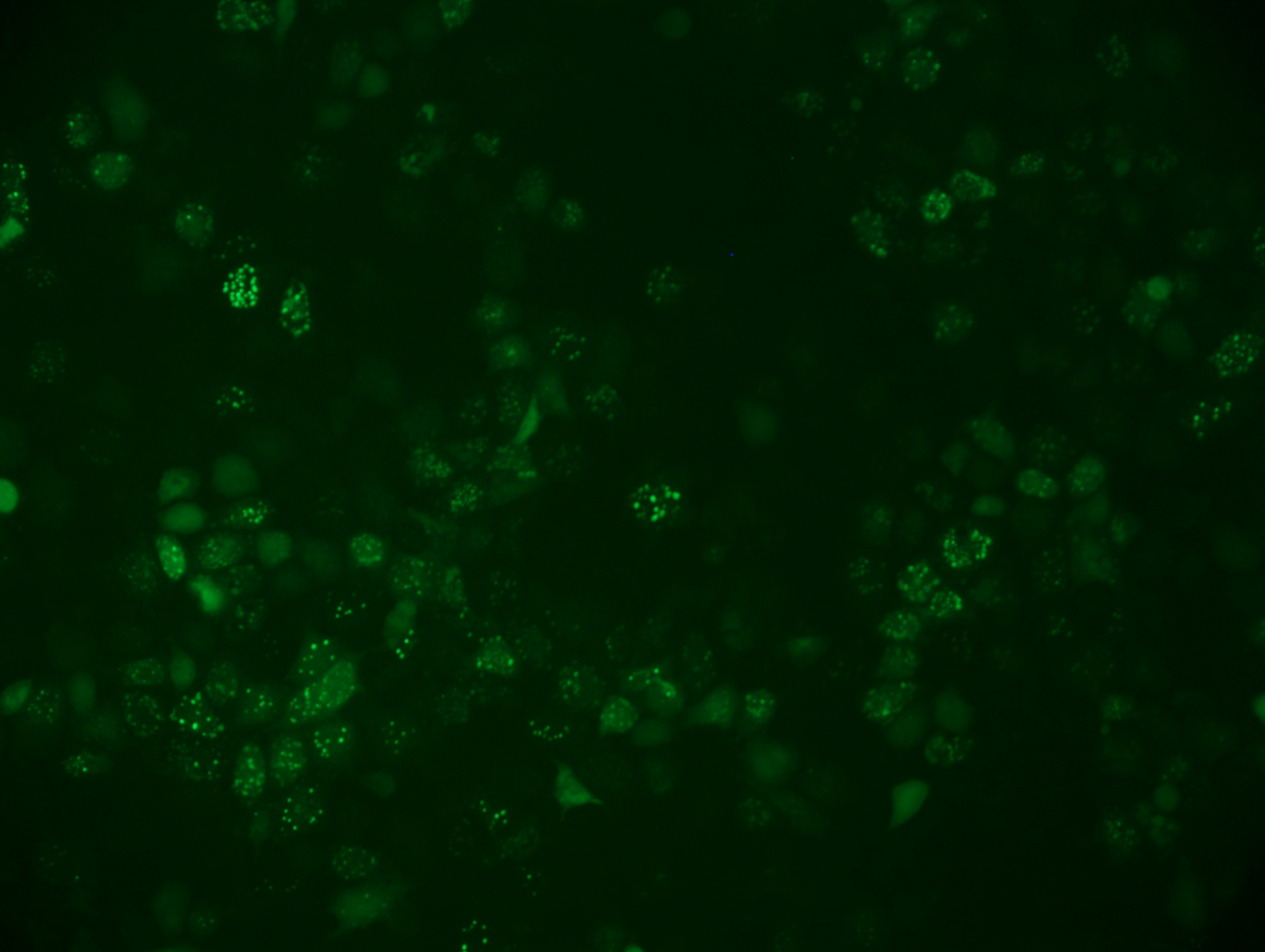

Supplement: Supplementary file 8 [file DataSheet_8.zip › fig 8/8D/HSV-1 C135 1.tif]

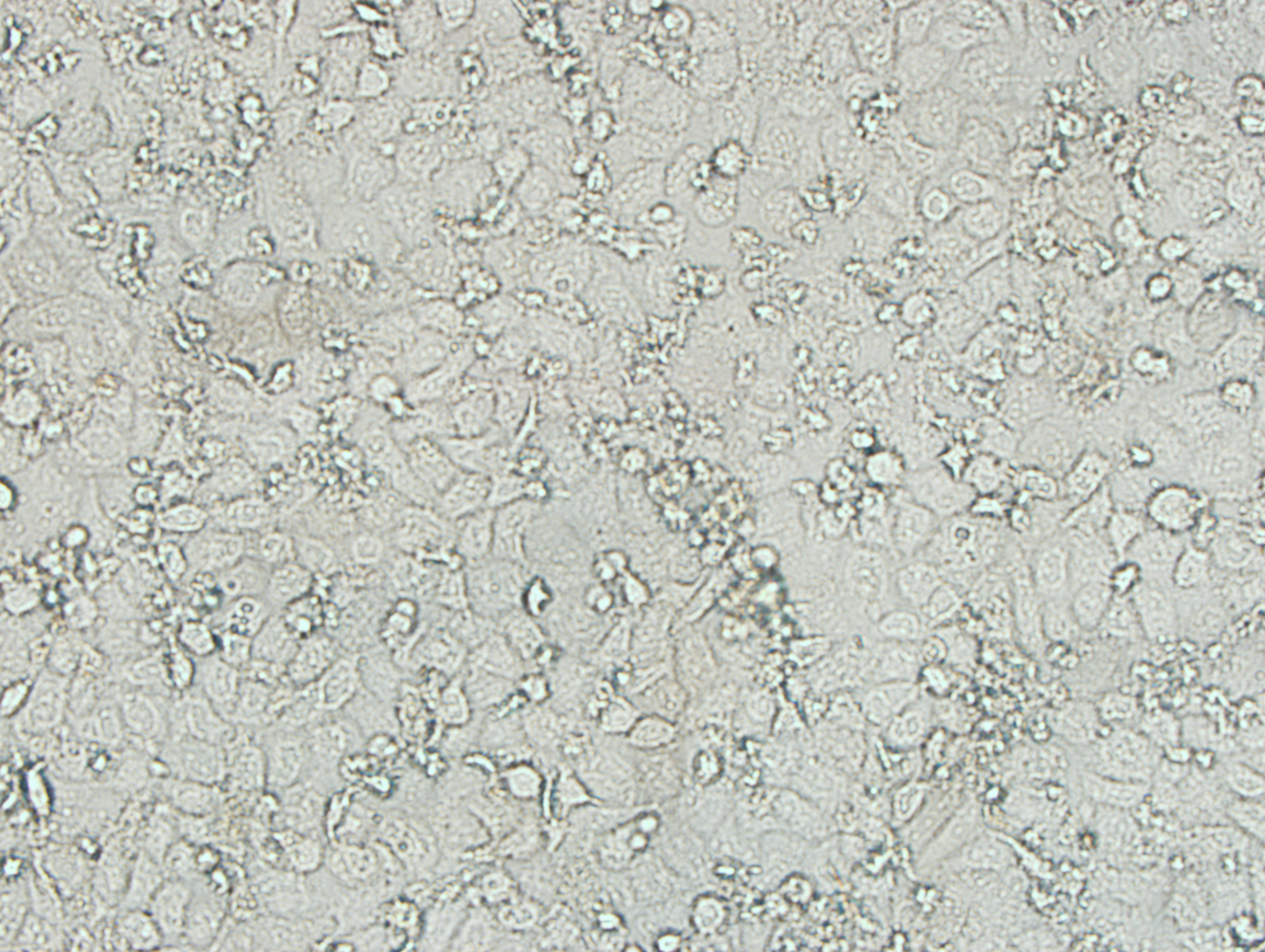

Supplement: Supplementary file 8 [file DataSheet_8.zip › fig 8/8D/HSV-1 C135 2.tif]

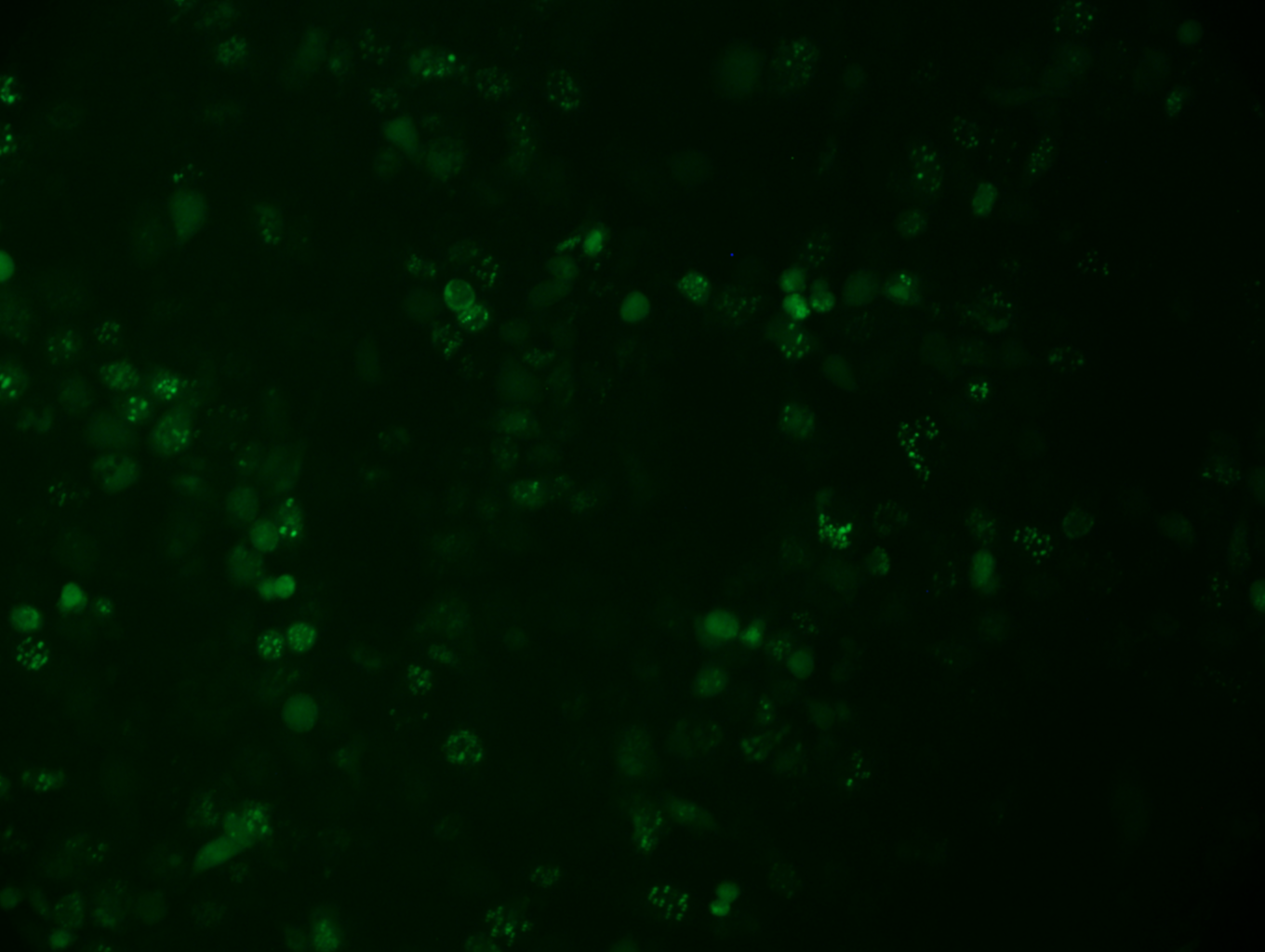

Supplement: Supplementary file 8 [file DataSheet_8.zip › fig 8/8D/HSV-1 EV 1.tif]

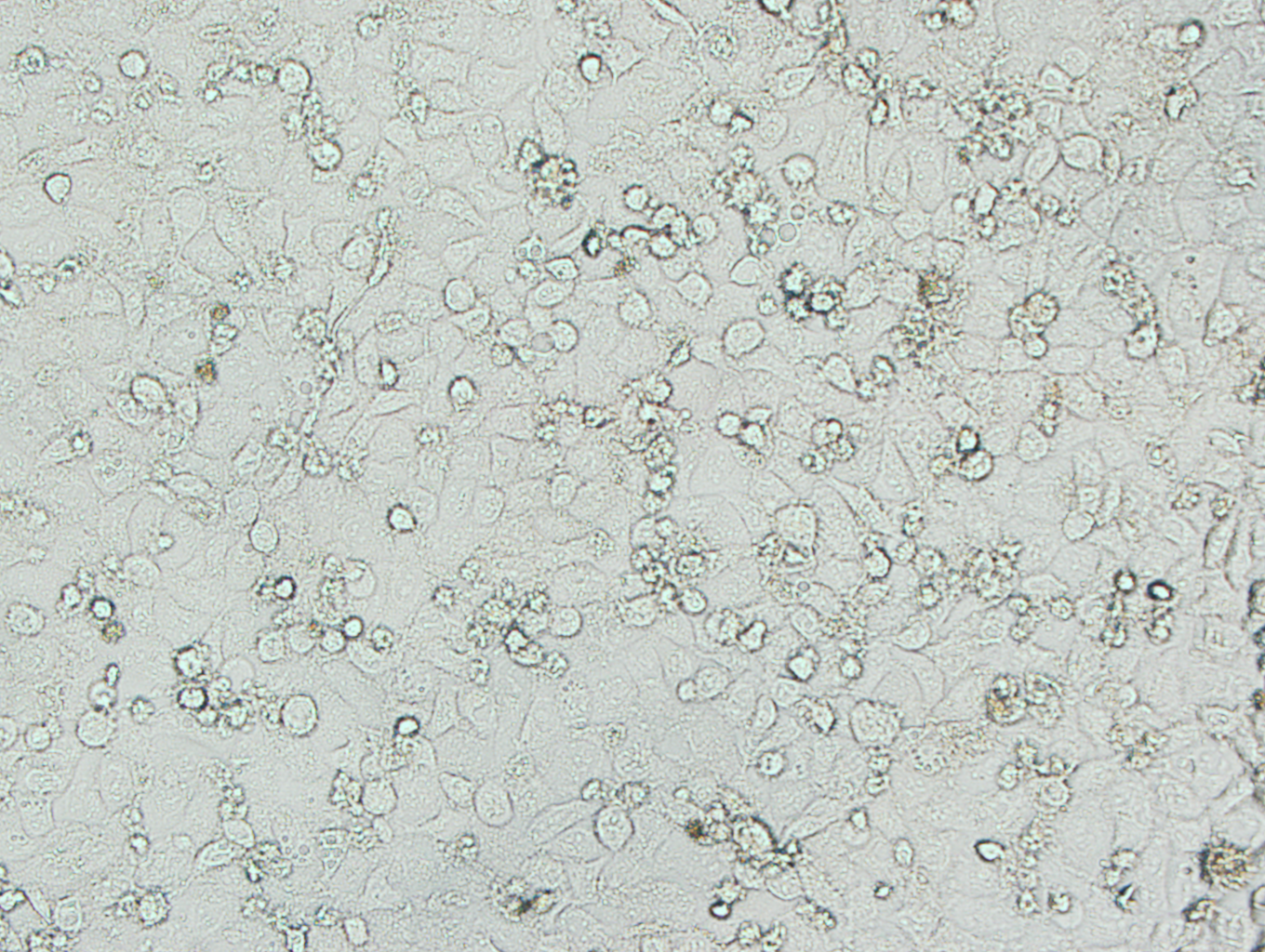

Supplement: Supplementary file 8 [file DataSheet_8.zip › fig 8/8D/HSV-1 EV 2.tif]

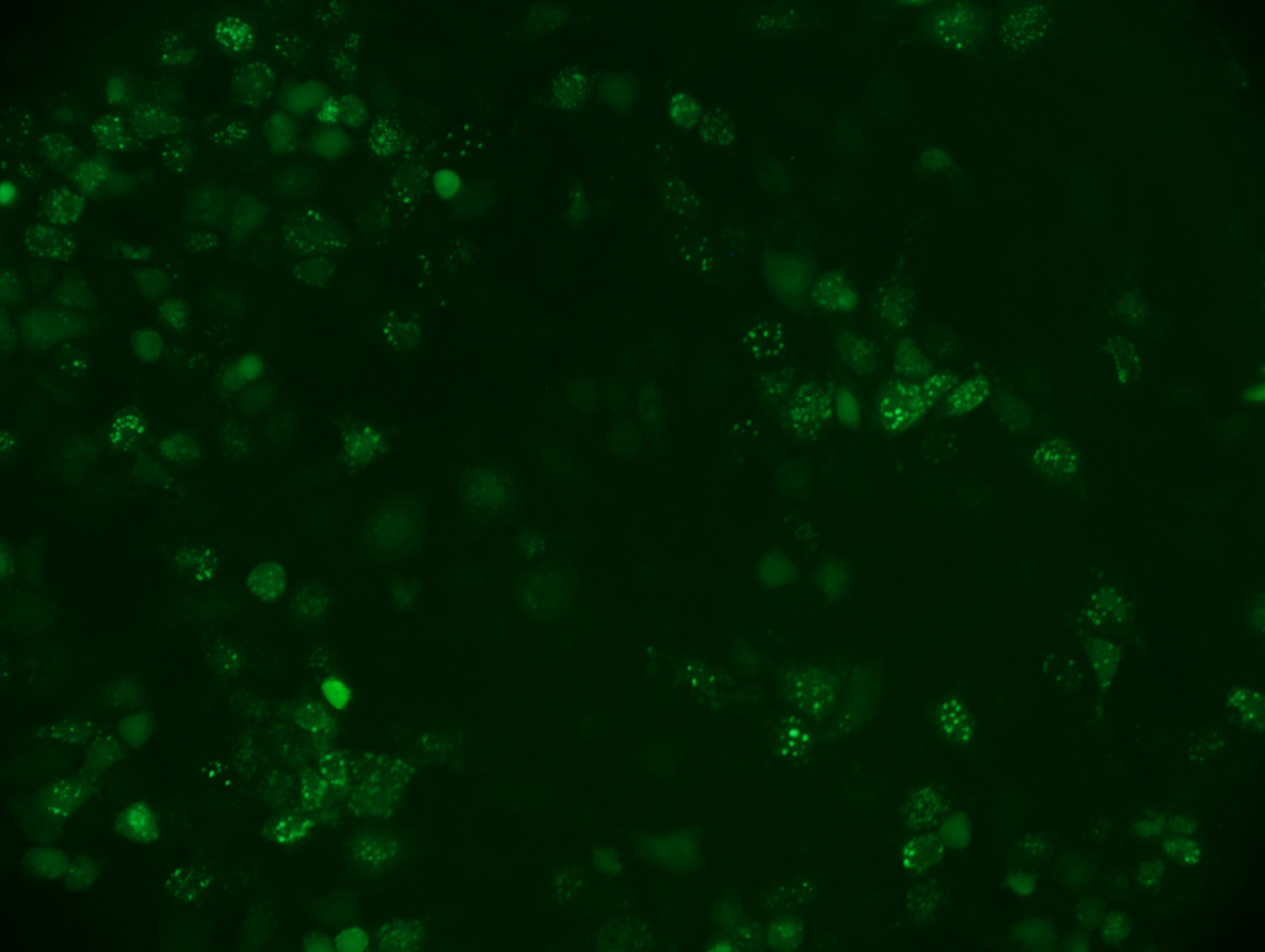

Supplement: Supplementary file 8 [file DataSheet_8.zip › fig 8/8D/HSV-1 H102 1.tif]

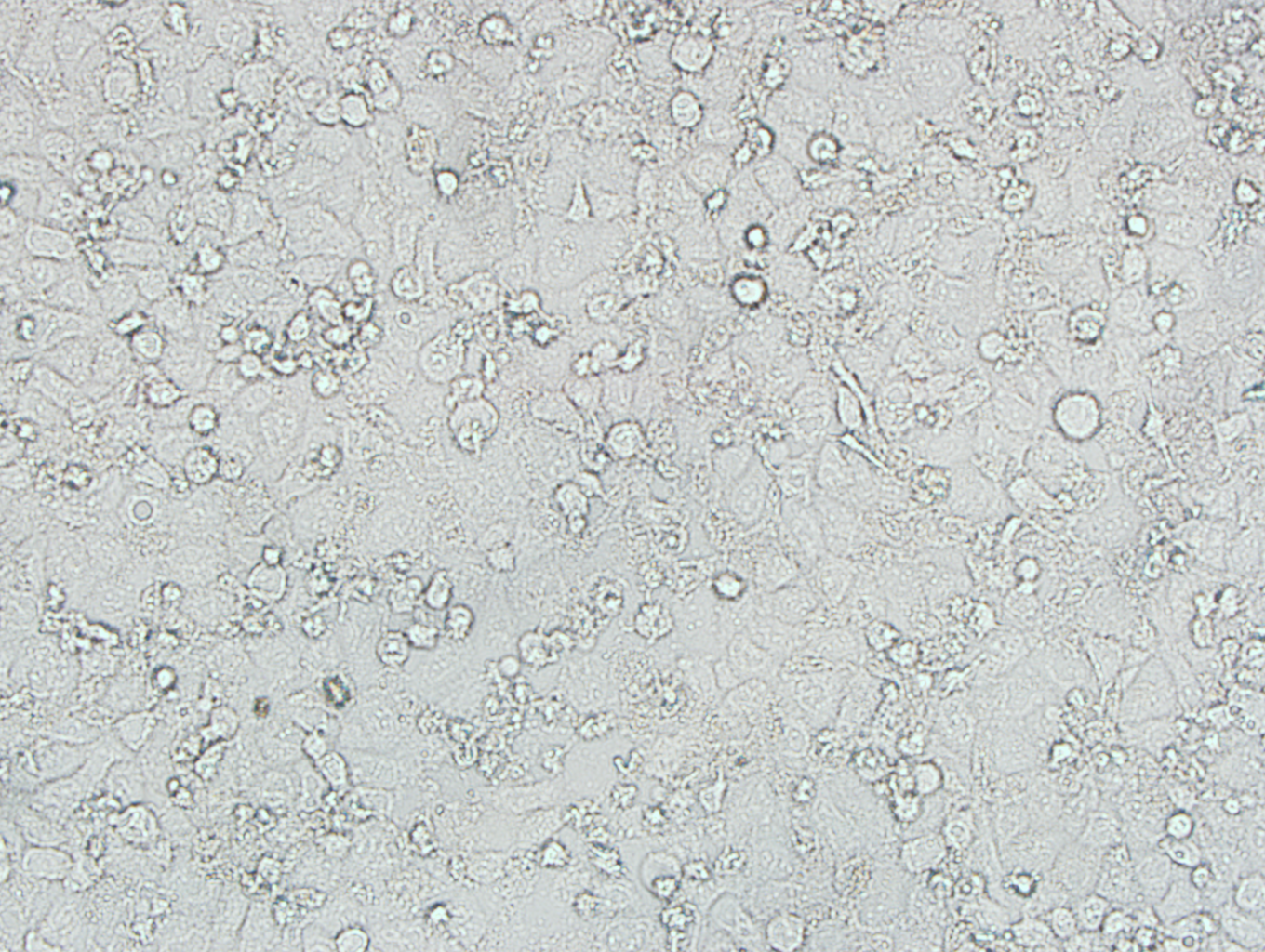

Supplement: Supplementary file 8 [file DataSheet_8.zip › fig 8/8D/HSV-1 H102 2.tif]

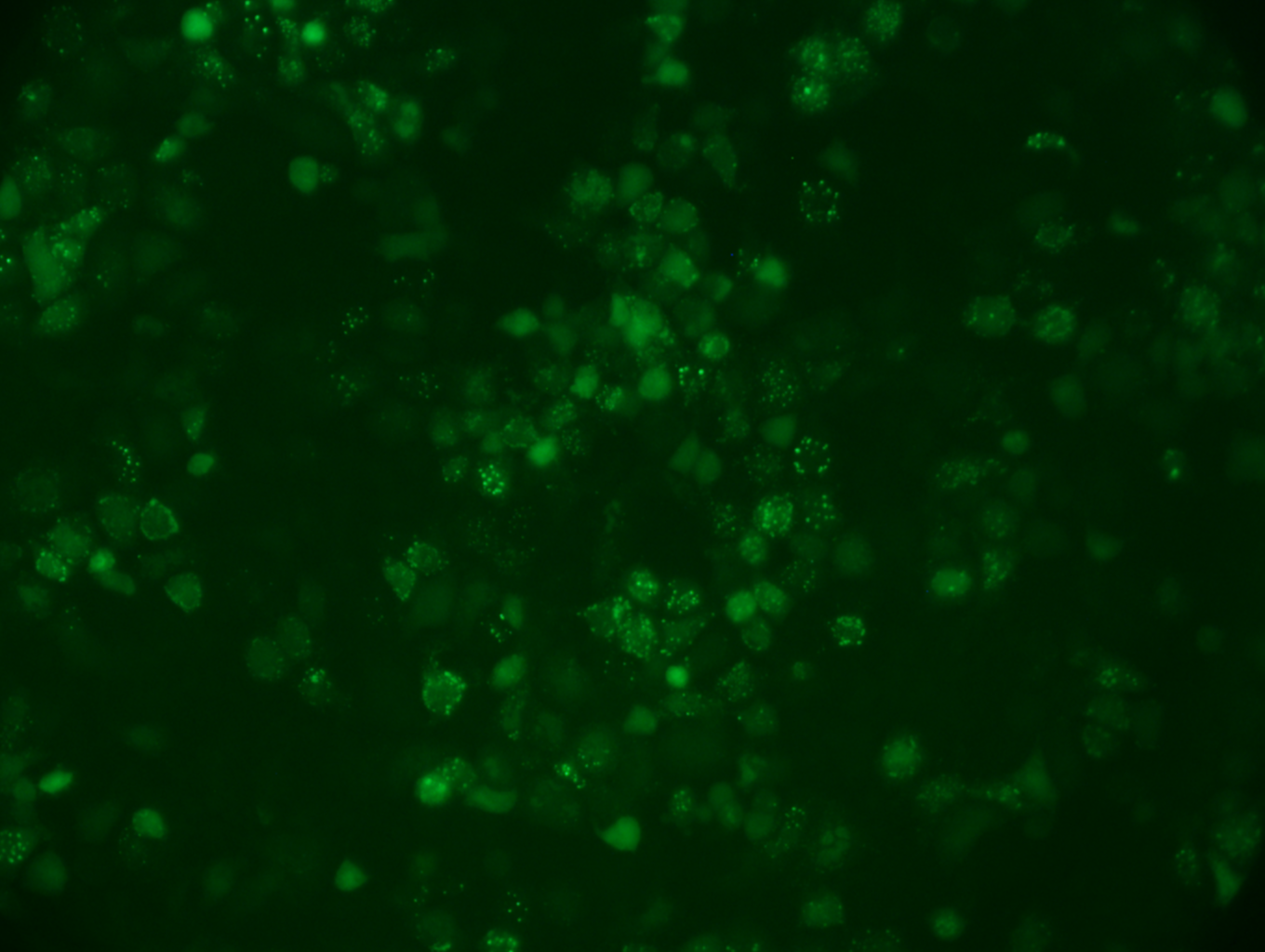

Supplement: Supplementary file 8 [file DataSheet_8.zip › fig 8/8D/HSV-1 WT 1.tif]

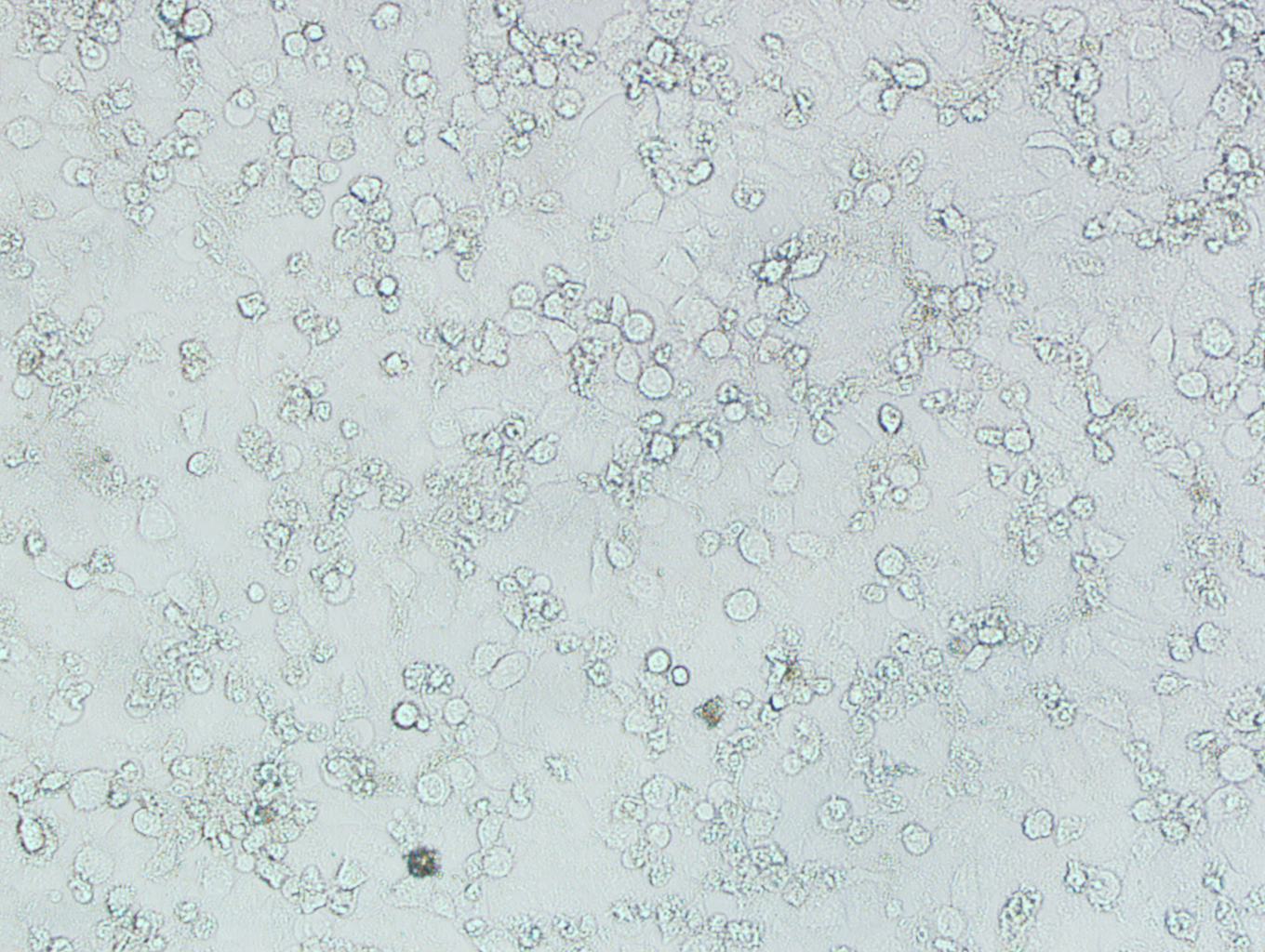

Supplement: Supplementary file 8 [file DataSheet_8.zip › fig 8/8D/HSV-1 WT 2.tif]

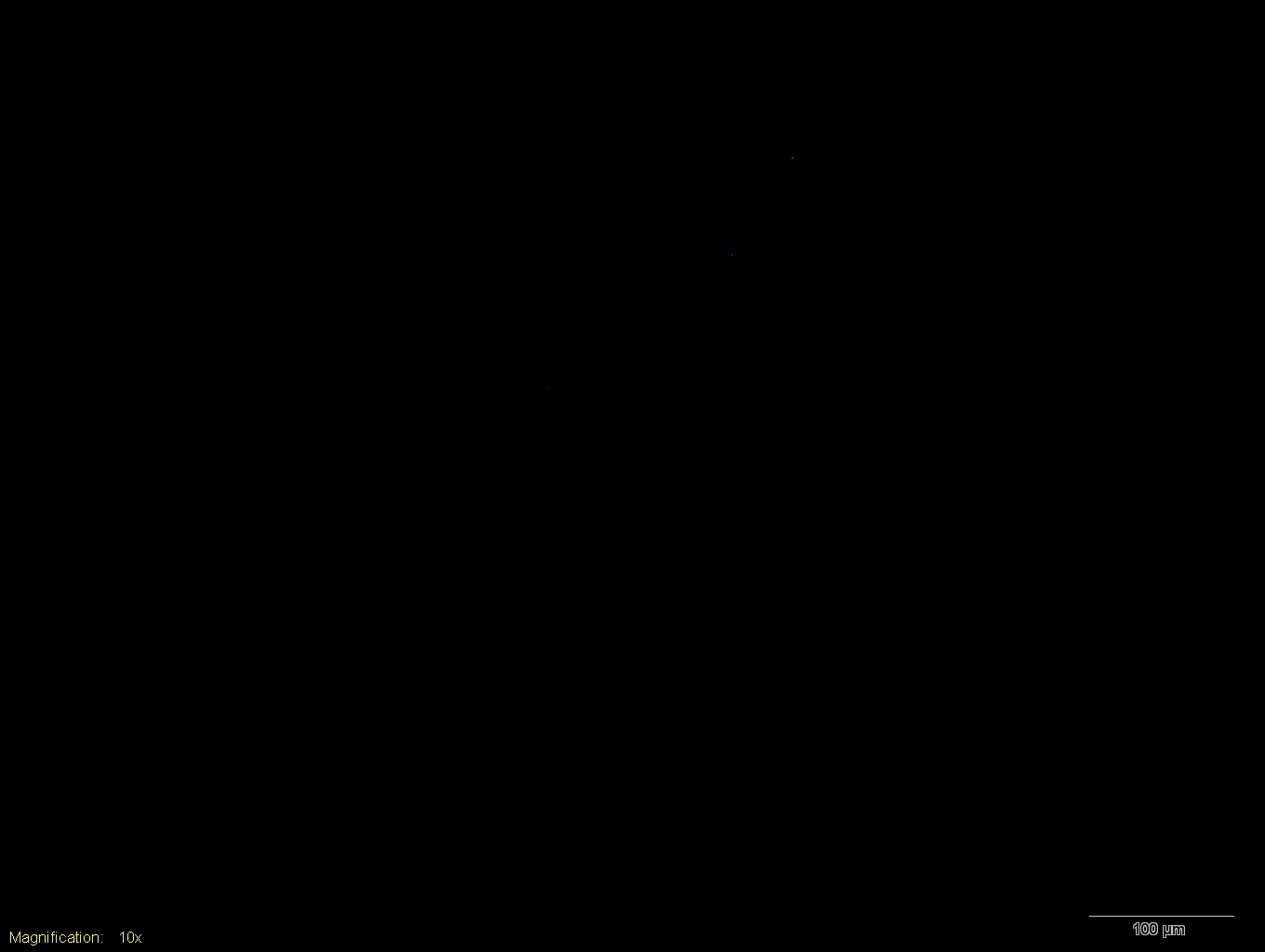

Supplement: Supplementary file 8 [file DataSheet_8.zip › fig 8/8D/mock 1.jpg]

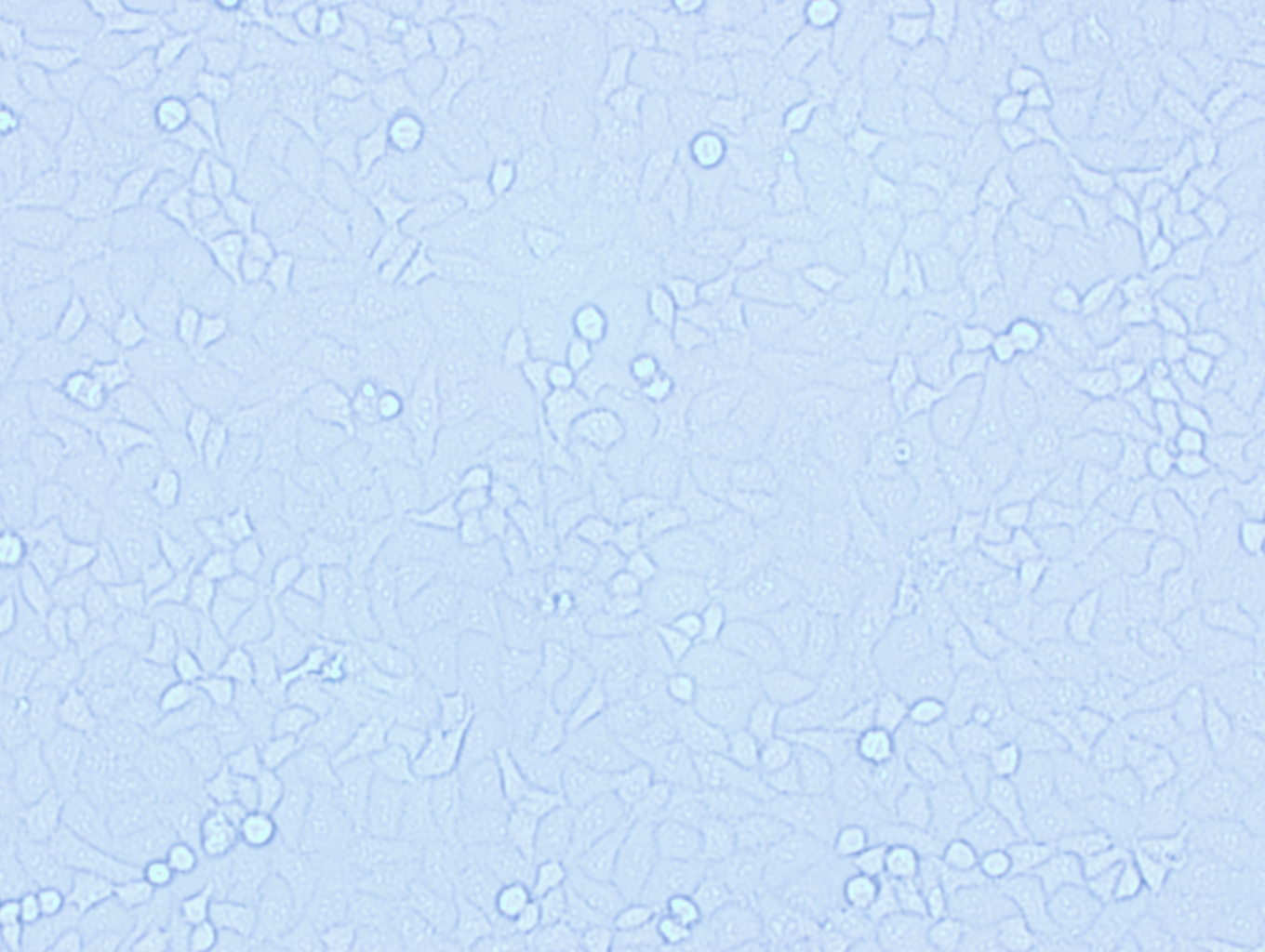

Supplement: Supplementary file 8 [file DataSheet_8.zip › fig 8/8D/mock 2.tif]

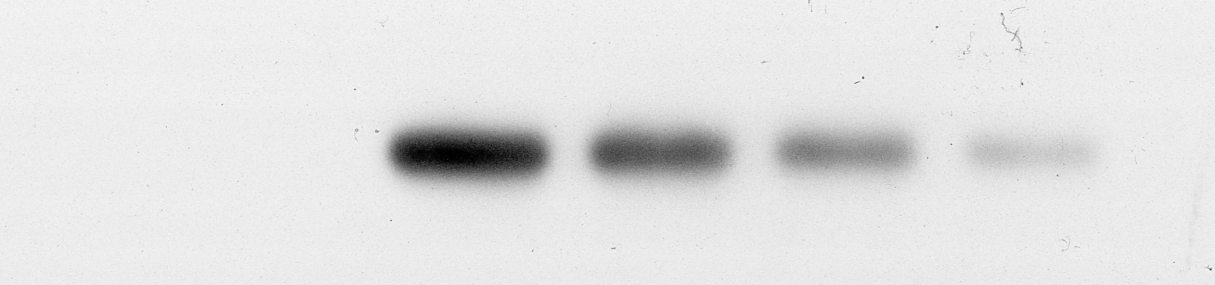

Supplement: Supplementary file 8 [file DataSheet_8.zip › fig 8/8F/A151155.jpg]

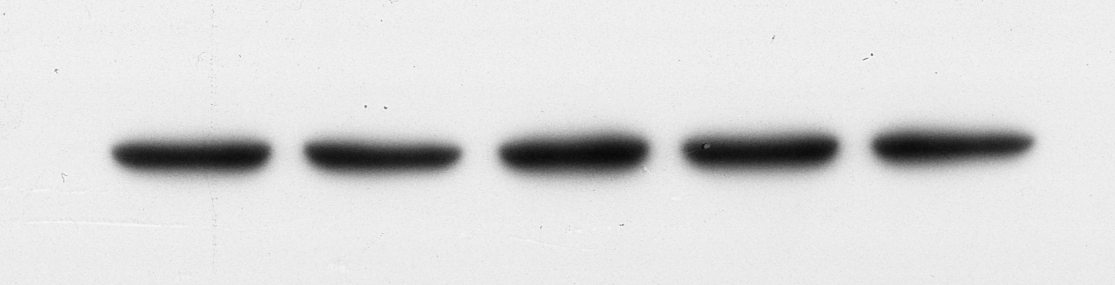

Supplement: Supplementary file 8 [file DataSheet_8.zip › fig 8/8F/GAPDH.jpg]

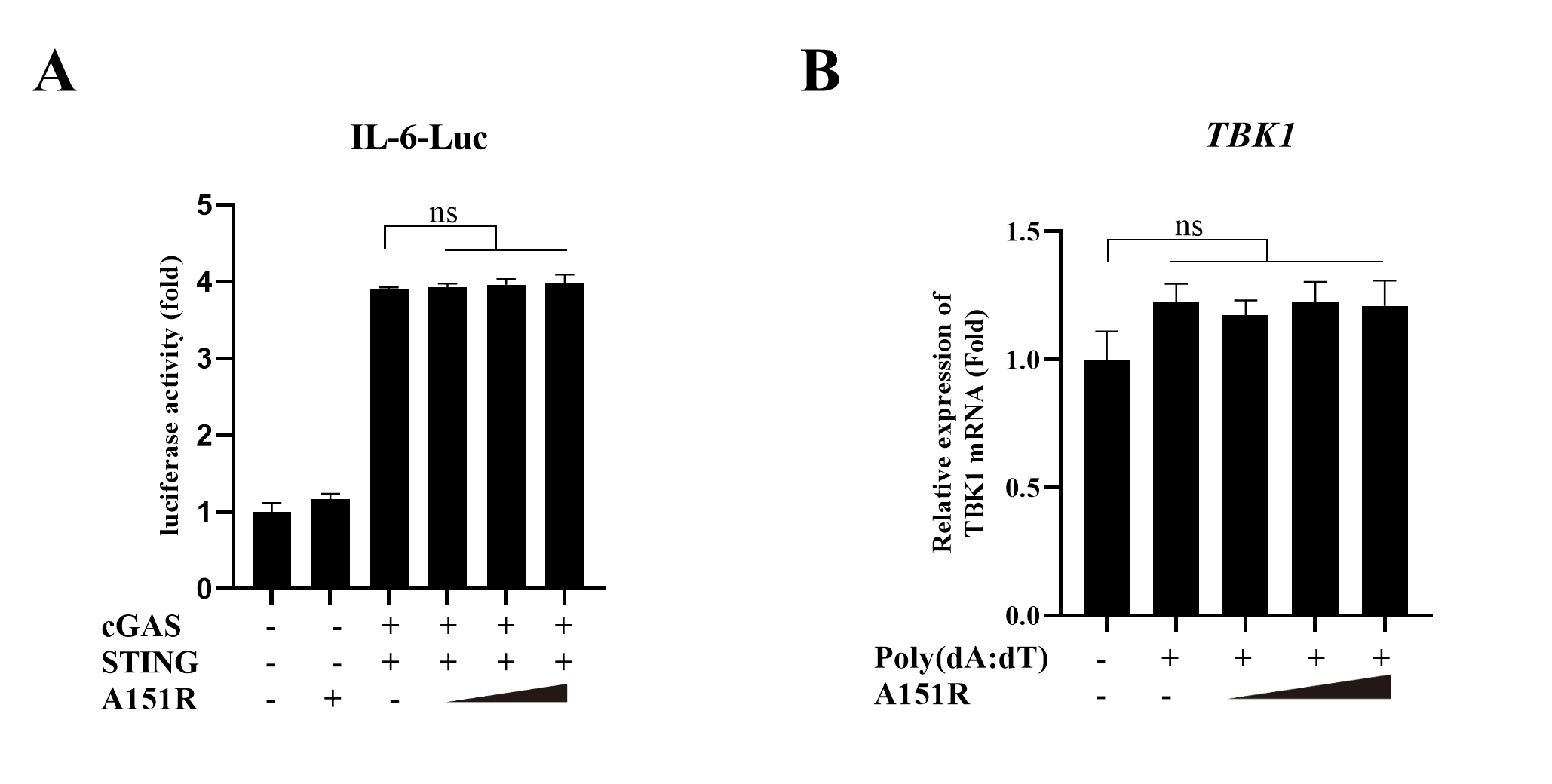

Supplement: Supplementary Figure 1 — ASFV pA151R negatively regulates type I IFN production induced by cGAS-STING. (A) HEK-293T cells cultured in 24-well plates were transfected with IL-6 luciferase (Luc) reporter (40 ng) and pRL-TK (4 ng), Flag-cGAS (20 ng) and Flag-STING (80 ng), and different doses of HA-A151R (0, 100, 200, or 400 ng) for 24 h followed by Dual-Luciferase assay. (B) CRL-2843 cells cultured in 24-well plates were transfected with different doses of HA-A151R (0, 100, 200, or 400 ng). At 24 hpt, cells were treated with poly(dA:dT) (1 μg/ml) for 12 h, and the mRNA levels of TBK1 was then analyzed by qRT-PCR assay. The data are representative of three independent experiments (means ± the standard errors of the mean [SEM]). ns, not significant (Student t test). [file Image_1.tif]

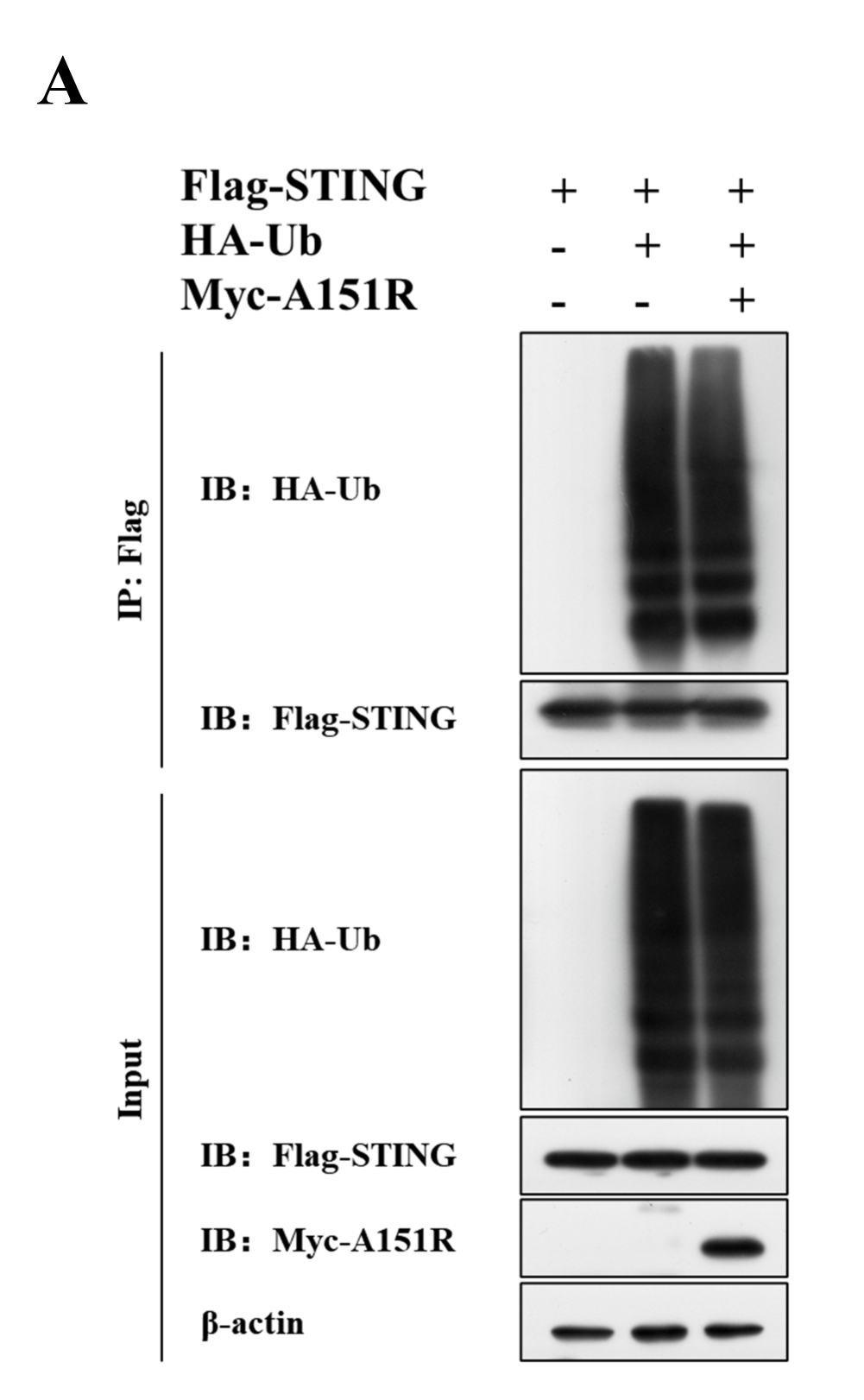

Supplement: Supplementary Figure 2 — ASFV pA151R inhibits TBK1 K63-linked polyubiquitination and phosphorylation. (A) Flag-STING and HA-ubiquitin (Ub) were co-transfected with or without Myc-A151R into HEK-293T cells cultured in 6-well plates for 24 h. The cell lysates were immunoprecipitated with anti-Flag (M2) beads for the detection of STING ubiquitination by using the indicated antibodies. [file Image_2.tif]
